# Cytarabine-induced differentiation of AML cells depends on Chk1 activation and shares the mechanism with inhibitors of DHODH and pyrimidine synthesis

**DOI:** 10.1038/s41598-022-15520-z

**Published:** 2022-07-05

**Authors:** Barbara Tomic, Tomislav Smoljo, Hrvoje Lalic, Vilma Dembitz, Josip Batinic, Drago Batinic, Antonio Bedalov, Dora Visnjic

**Affiliations:** 1 Croatian Institute for Brain Research, University of Zagreb School of Medicine, Salata 3, 10 000 Zagreb, Croatia; 2 Department of Physiology, University of Zagreb School of Medicine, Zagreb, Croatia; 3 Division of Hematology, Department of Internal Medicine, University Hospital Centre Zagreb, Zagreb, Croatia; 4 Department of Laboratory Immunology, University Hospital Centre Zagreb, Zagreb, Croatia; 5 Clinical Research Division, Fred Hutchinson Cancer Research Centre, Seattle, WA USA

**Keywords:** Acute myeloid leukaemia, Cell-cycle exit

## Abstract

Acute myeloid leukemia (AML) is characterized by arrested differentiation making differentiation therapy a promising treatment strategy. Recent success of inhibitors of mutated isocitrate dehydrogenase (IDH) invigorated interest in differentiation therapy of AML so that several new drugs have been proposed, including inhibitors of dihydroorotate dehydrogenase (DHODH), an enzyme in pyrimidine synthesis. Cytarabine, a backbone of standard AML therapy, is known to induce differentiation at low doses, but the mechanism is not completely elucidated. We have previously reported that 5-aminoimidazole-4-carboxamide ribonucleoside (AICAr) and brequinar, a DHODH inhibitor, induced differentiation of myeloid leukemia by activating the ataxia telangiectasia and Rad3-related (ATR)/checkpoint kinase 1 (Chk1) via pyrimidine depletion. In this study, using immunoblotting, flow cytometry analyses, pharmacologic inhibitors and genetic inactivation of Chk1 in myeloid leukemia cell lines, we show that low dose cytarabine induces differentiation by activating Chk1. In addition, cytarabine induces differentiation ex vivo in a subset of primary AML samples that are sensitive to AICAr and DHODH inhibitor. The results of our study suggest that leukemic cell differentiation stimulated by low doses of cytarabine depends on the activation of Chk1 and thus shares the same pathway as pyrimidine synthesis inhibitors.

## Introduction

Acute myeloid leukemia (AML) is a severe hematological malignancy characterized by clonal expansion of blasts and arrested differentiation, making therapy aimed at differentiation a promising treatment strategy^1,2^. The most successful example of differentiation therapy is all-trans retinoic acid (ATRA) based treatment of acute promyelocytic leukemia (APL), a subtype of AML carrying *t(15;17)* translocation and PML-RARA transcript. The combination of ATRA and arsenic trioxide can achieve a complete remission rate of nearly 100% and long-term survival rates of 98% in APL, turning once fatal disease into the most curable subtype of AML^3^. However, ATRA has not been clinically proved generally effective in non-APL AML, suggesting that mechanisms of differentiation arrest are not uniform for all AML subtypes. Since 2017, novel drugs that induce differentiation of AML cells by inhibiting mutated isocitrate dehydrogenase (IDH)1/2 and FLT3 have been approved for AML therapy. Enasidenib, an inhibitor of mutated IDH2, restored normal enzyme activity and induced differentiation and durable remissions even in elderly patients with multiple comorbidities^4–6^, ivosidenib, an inhibitor of mutated IDH1, induced myeloid differentiation of AML blasts and durable remissions in patients with newly diagnosed IDH1-mutant AML ineligible for standard chemotherapy^7^, and gilteritinib, a type I FLT3 inhibitor, induced differentiation in relapsed and refractory *FLT3*-mutated AML^8^. In addition, several new targets for AML differentiation therapy have emerged through mutational and gene expression analysis, and some of them, like inhibitors of dihydroorotate dehydrogenase (DHODH), are being actively explored^2,9^.

Cytarabine (1-β-D-arabinofuranosylcytosine, cytosine arabinoside or AraC) has been the backbone of the standard first-line induction therapy in adults with AML for the last five decades. Most patients with newly diagnosed AML who are suitable candidates for intensive chemotherapy are offered the combination of standard-dose cytarabine with an anthracycline, the so-called “7 + 3” regimen. However, the median age of AML patients is 68 years, and the majority of older patients have multiple comorbidities making them unfit for intensive chemotherapy so that an acceptable low-intensity therapeutic strategy for this population includes hypomethylating agents, like decitabine or azacitidine, or low-dose cytarabine (LDAC)^1^. The proposed mechanism of cytarabine action includes its rapid conversion into cytosine arabinoside triphosphate (AraCTP), which interferes with DNA replication by multiple mechanisms, primarily by incorporation into DNA molecule. Cytotoxic effect of high doses of cytarabine on AML cells is ascribed to apoptosis, but low doses of cytarabine triggered AML remissions without toxicity and induced maturation of leukemic cells in vivo suggesting that cytarabine is capable of differentiation induction^10,11^. Although granulocytic or monocytic differentiation in response to cytarabine have been confirmed in several AML cell lines, the mechanism responsible for differentiation remained incompletely understood^12–14^. In recent years, cytarabine has been used in differentiation therapy mostly to enhance the effects of other differentiation-inducing agents such as inhibitors of Aurora kinase or FLT3^2^.

Our recent studies demonstrated that pyrimidine synthesis inhibitors induce differentiation of myeloid leukemia by activating the ataxia telangiectasia and Rad3-related (ATR)/checkpoint kinase 1 (Chk1) DNA damage signaling pathway via pyrimidine depletion. 5-aminoimidazole-4-carboxamide ribonucleoside (AICAr), a precursor in purine biosynthesis and a well-established activator of AMP-kinase, inhibited uridine monophosphate (UMP) synthesis, a step downstream of DHODH in pyrimidine synthesis, in an AMPK-independent manner. AICAr and the DHODH inhibitor brequinar had similar effects on differentiation markers and S-phase arrest, while genetic and pharmacological Chk1 inactivation abrogated both of these effects^15,16^. Cytarabine incorporation into DNA during the process of DNA synthesis eventually causes DNA damage by staling replication forks, and ATR/Chk1 pathway is known to promote survival by signaling replication stress^17–19^. In this study, we tested the role of Chk1 in differentiation of AML cells induced by cytarabine and compared the effects of cytarabine with the effects of AICAr and DHODH inhibitor brequinar.

## Results

### Cytarabine dose-dependently decreases proliferation, induces differentiation and activates Chk1

**Figure 1:**
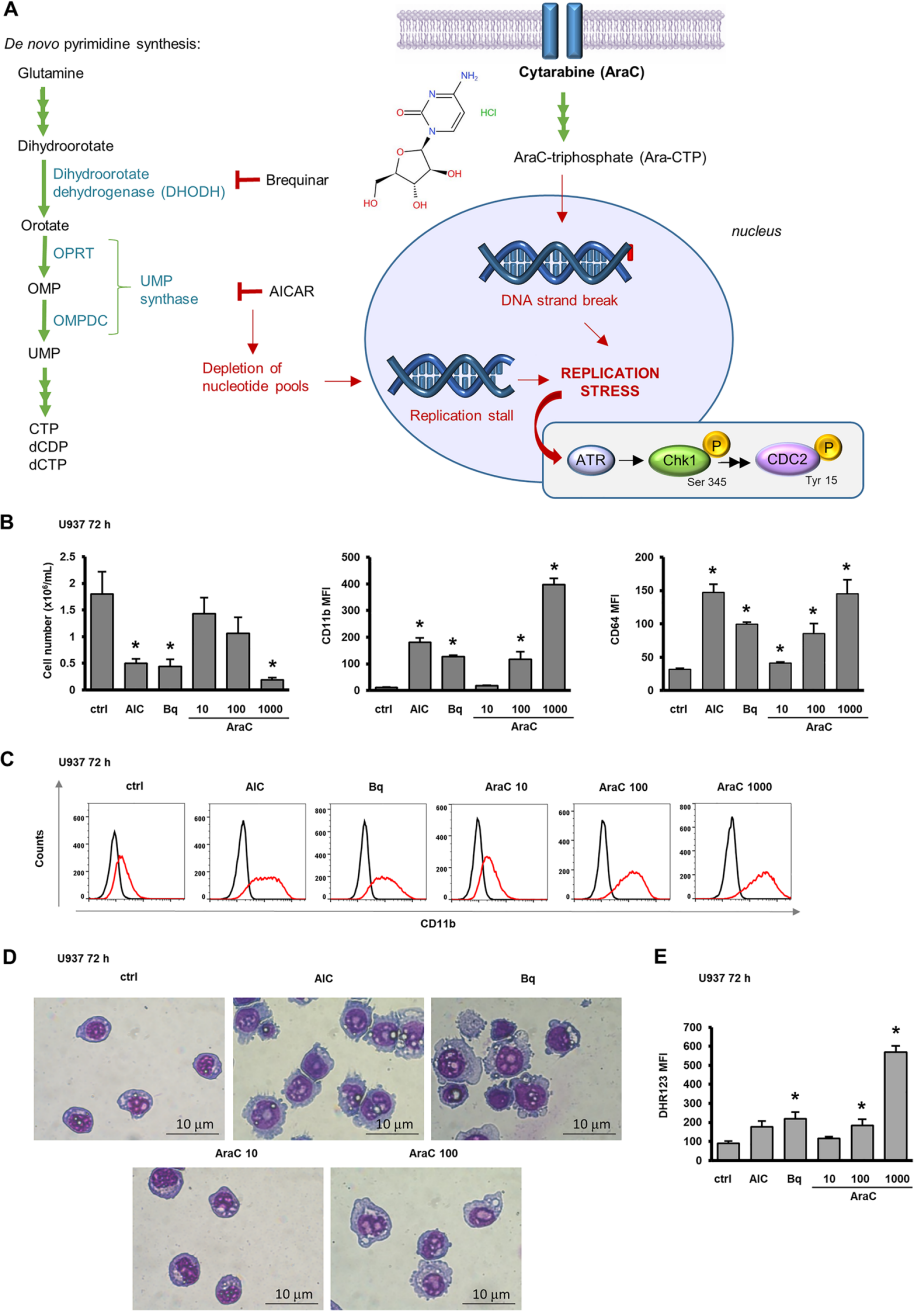
Cytarabine dose-dependently decreases cell number and induces differentiation. (**A**) DNA damage pathway activation by pyrimidine synthesis inhibitors and cytarabine. (**B**) U937 cells were incubated with AICAr (AIC) (0.2 mM), brequinar (Bq) (0.5 µM) and AraC (10, 100, 1000 nM). The number of viable cells and the expression of differentiation markers were determined after 72 h. Mean fluorescence intensity (MFI) of CD11b and CD64 was calculated as described under “Methods” section. Results are mean ± S.E. (error bars) of at least three independent experiments. *, *p* < 0.05 (Student's *t*-test) compared with control (ctrl). (**C**) Representative histograms (out of three independent flow cytometric analyses shown in B) with black line representing isotypic control and red line representing the expression of CD11b. (**D**) Morphological analysis of U937 cells treated with AICAr (AIC) (0.2 mM), brequinar (Bq) (0.5 µM) and AraC (10 and 100 nM). May-Grünwald-Giemsa stained cytospin preparations (100 × magnification). **(E)** Respiratory burst in U937 cells treated with AICAr (AIC) (0.2 mM), brequinar (Bq) (0.5 µM) and AraC (10, 100, 1000 nM) for 72 h. Results are mean ± S.E. (error bars) of at least three independent experiments. *, *p* < 0.05 (Student's t-test) compared with control (ctrl).

Results of our previous study showed that two drugs that inhibit de novo pyrimidine synthesis induce differentiation of AML cells and activate Chk1 pathway^15^. As shown in Fig. 1A, AICAr and brequinar cause replication stress by the depletion of nucleotide pools available for DNA synthesis. Cytarabine (AraC) is phosphorylated into AraCTP and competes directly with dCTP for incorporation into newly synthesized DNA causing a delay in replication fork progression and generating double strand DNA breaks. ATR/Chk1 is activated in response to stalled replication forks and inhibits the activity of the CDC2/cyclin-dependent kinase 1 (CDK1) necessary for the G_2_/M transition^17,18^.

To test for the effects of AraC on differentiation and Chk1 activation, exponentially growing U937 cells were exposed to various concentrations of AraC (0–1000 nM) for 72 h and the effects on the number of viable cells and the expression of differentiation markers were compared with the effects of AICAr (0.2 mM) and brequinar (500 nM). As shown in Fig. 1B, AraC dose-dependently reduced the number of viable cells, and the reduced proliferation was paralleled by an increase in the expression of differentiation markers CD11b and CD64 (Supplementary Fig. 1). Histograms were strictly unimodal in control cells and 100 nM AraC increased the expression of CD11b to the level similar to the one observed in response to AICAr and brequinar (Fig. 1C). These results were supported by morphological analysis that revealed an increase in cell size and vacuolization with a decrease in nucleo/cytoplasmic ratio (Fig. 1D) and by an assay that revealed a significant increase in respiratory burst in U937 cells treated with AraC (100 and 1000 nM) for 72 h (Fig. 1E).

**Figure 2:**
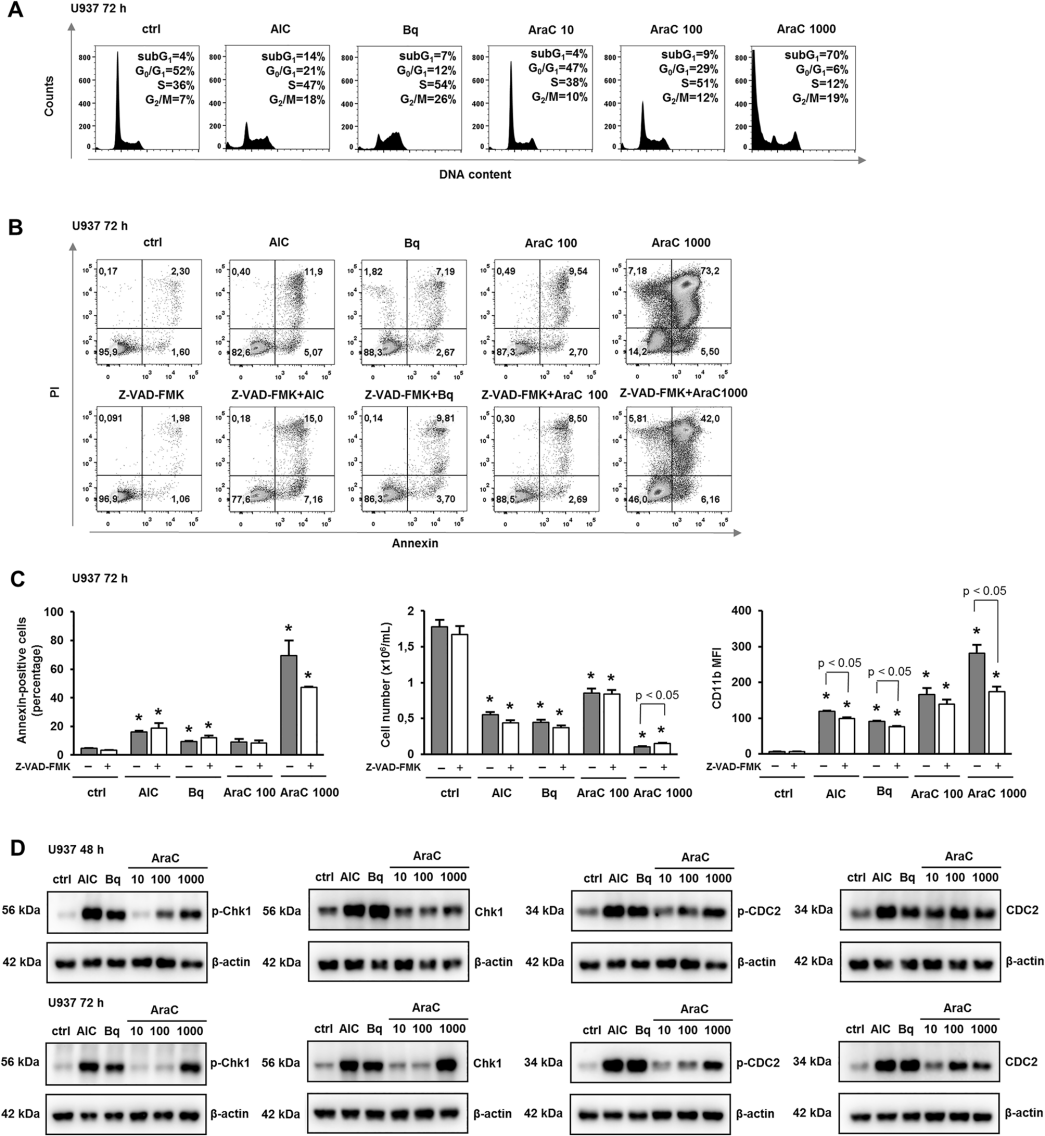
Cytarabine induces cell cycle arrest and activates Chk1. U937 cells were incubated with AICAr (AIC) (0.2 mM), brequinar (Bq) (0.5 µM) and AraC (10, 100, 1000 nM). (**A**) Representative histograms of propidium-labelled cells from three independent experiments analyzed by flow cytometry. (**B, C**) Pan-caspase inhibitor Z-VAD-FMK (10 μM) was added 30 min before the addition of agents. (**B**) The representative dot plots of cells stained with annexin V-FITC/PI and analyzed by flow cytometry. (**C**) The percentage of annexin V-FITC-positive cells, the number of viable cells and the expression of differentiation markers were determined after 72 h. Results are mean ± S.E. (error bars) of at least three independent experiments. *, *p* < 0.05 (Student's t-test) compared with control (ctrl). (**D**) U937 cells were incubated with AICAr (AIC) (0.2 mM), brequinar (Bq) (0.5 µM) and AraC (10, 100, 1000 nM) for 48 h (upper panels) or 72 h (lower panels). Total cell lysates were isolated after 48 and 72 h and analyzed by Western blotting for the level of Ser-345-phosphorylated Chk1, total Chk1, Tyr-15-phosphorylated CDC2 and total CDC2. Representative immunoblots from three independent experiments are shown.

Flow cytometric analyses of the progression through the cell cycle revealed that both AICAr and brequinar induced marked increases in the proportion of cells in S-phase, as previously described^15^. AraC dose-dependently decreased the proportion of cells in G_1_-phase and increased the proportion of S-phase in cells treated with 10 and 100 nM. When applied at the highest dose (1000 nM), AraC caused a marked increase in the proportion of subG_1_ and G_2_/M-phase (Fig. 2A). To further investigate the role of apoptosis in AraC-mediated effects, the percentage of annexin-positive cells was determined after 72 h. The percentage of annexin-FITC-positive cells upon treatment with low dose AraC (100 nm) was similar to the one observed in response to AICAr and brequinar and much lower than the one measured in cells treated with 1000 nM Ara-C (Fig. 2B). As shown in Fig. 2C, the presence of pan-caspase inhibitor Z-VAD-FMK at a dose (10 μM) that had no effects on U937 cells alone, had significant effects on cells treated with high dose AraC (1000 nM) by reducing the percentage of annexin-positive cells, increasing the number of viable cells, and decreasing the expression of CD11b. However, the effects of low dose AraC (100 nM) on the number of viable cells and the expression of CD11b were not prevented by the presence of pan-caspase inhibitor, suggesting that cell cycle arrest in response to low dose AraC was due to differentiation and not to the loss of viability due to cell death.

To test whether cell cycle arrest is associated with Chk1 activation, the level of Ser-345-phosphorylated Chk1 was determined in cells treated with agents for 48 and 72 h. Again, AraC mimicked the effects of AICAr and brequinar and dose-dependently increased the level of phosphorylated Chk1 at both time points and increased the level of total protein after longer incubation (Fig. 2D). In addition, the level of phosphorylated Chk1 was mirrored by an increase in CDC2 (CDK1) phosphorylation on Tyr-15, which is a marker of the level of CDC2 inhibition. Therefore, we concluded that cytarabine induced cell differentiation with a parallel block in cell cycle progression, Chk1 activation and CDC2 inhibition, and that the effects of AraC mimicked the effects of pyrimidine synthesis inhibitors.

Our previous study^15^ revealed that AICAr and brequinar inhibited pyrimidine synthesis and that addition of nucleosides or uridine alone abolished their effects on Chk1 activation and leukemia cell differentiation. According to Fig. 1A, the activation of Chk1 in response to AraC should be due to the incorporation into DNA and not to the lack of nucleotides. To further distinguish between the two, we performed rescue experiments by addition of mixture of nucleosides to U937 cells treated with AraC. As shown in Supplementary Fig. 2, the addition of nucleosides abolished differentiation induced by either AICAr or brequinar, but had no effects on AraC-induced increase in the expression of CD11b and CD64. Therefore, we concluded that, although both AraC and pyrimidine synthesis inhibitors activated Chk1 and induced differentiation, AraC-mediated effects are not due to the nucleotide depletion.

### Pharmacological inhibition of ATR/Chk1 pathway prevents differentiation and cell cycle arrest

**Figure 3:**
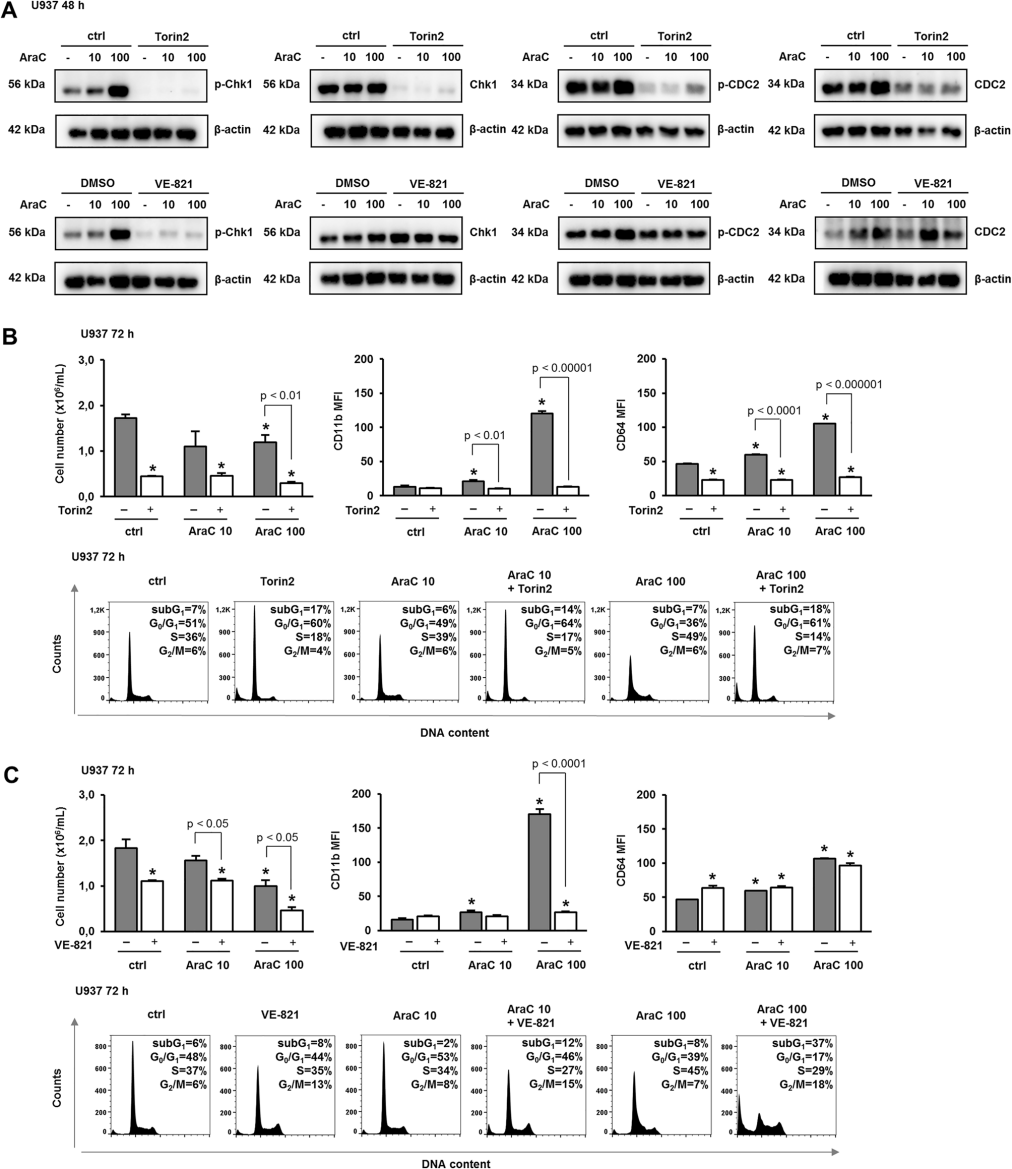
Pharmacological inhibition of ATR/Chk1 pathway prevents differentiation and cell cycle arrest. U937 cells were grown in the presence of increasing concentrations of AraC (10, 100 nM). Torin2 (100 nM), VE-821 (10 µM) or vehicle (DMSO) were added 30 min before the addition of AraC. **(A)** Total cell lysates were isolated after 48 h and analyzed by Western blotting for the level of Ser-345-phosphorylated Chk1, total Chk1, Tyr-15-phosphorylated CDC2 and total CDC2. Representative immunoblots from three independent experiments are shown. (**B**–**C**) The number of viable cells, the expression of differentiation markers and the cell cycle progression were determined for Torin2 **(B)** and VE-821 **(C)** pre-treated cells as described under “Methods” section. Results are mean ± S.E. (error bars) of at least three independent experiments. *, *p* < 0.05 (Student's *t*-test) compared with control (ctrl).

To test for the role of Chk1 in cytarabine-mediated cell differentiation, we first incubated cells with pharmacological ATR inhibitors, Torin2 and VE-821, since we have previously shown that both compounds inhibited the effects of AICAr and brequinar^15^. We first tested for the doses that efficiently inhibit Chk1 activation in U937 cells. As shown in Fig. 3A, both Torin2 and VE-821 abolished AraC-mediated increase in the level of Ser-345-phosphorylated Chk1, but VE-821 was less potent in preventing CDC2 phosphorylation on Tyr-15. When applied at the concentration of 100 nM, Torin2 significantly decreased the number of viable cells, completely prevented the increase in the expression of differentiation markers and arrested both control and AraC-treated cells in G_0_/G_1_-phase of the cell cycle (Fig. 3B and Supplementary Fig. 3). As shown in Fig. 3C, VE-821 (10 µM) reduced the number of viable cells and inhibited the expression of differentiation markers in response to AraC. As expected from the effects on CDC2, the effects of VE-821 alone on the progression through the cell cycle differed from the effects of Torin2. However, since both inhibitors were capable of inhibiting the activity of Chk1, these results suggest that Chk1 may have a role in AraC-mediated differentiation of U937 cells.

### Down-regulation of Chk1 reduced the effects of cytarabine on the expression of differentiation markers and S-phase arrest with a reciprocal increase in AraC-related cell death

**Figure 4:**
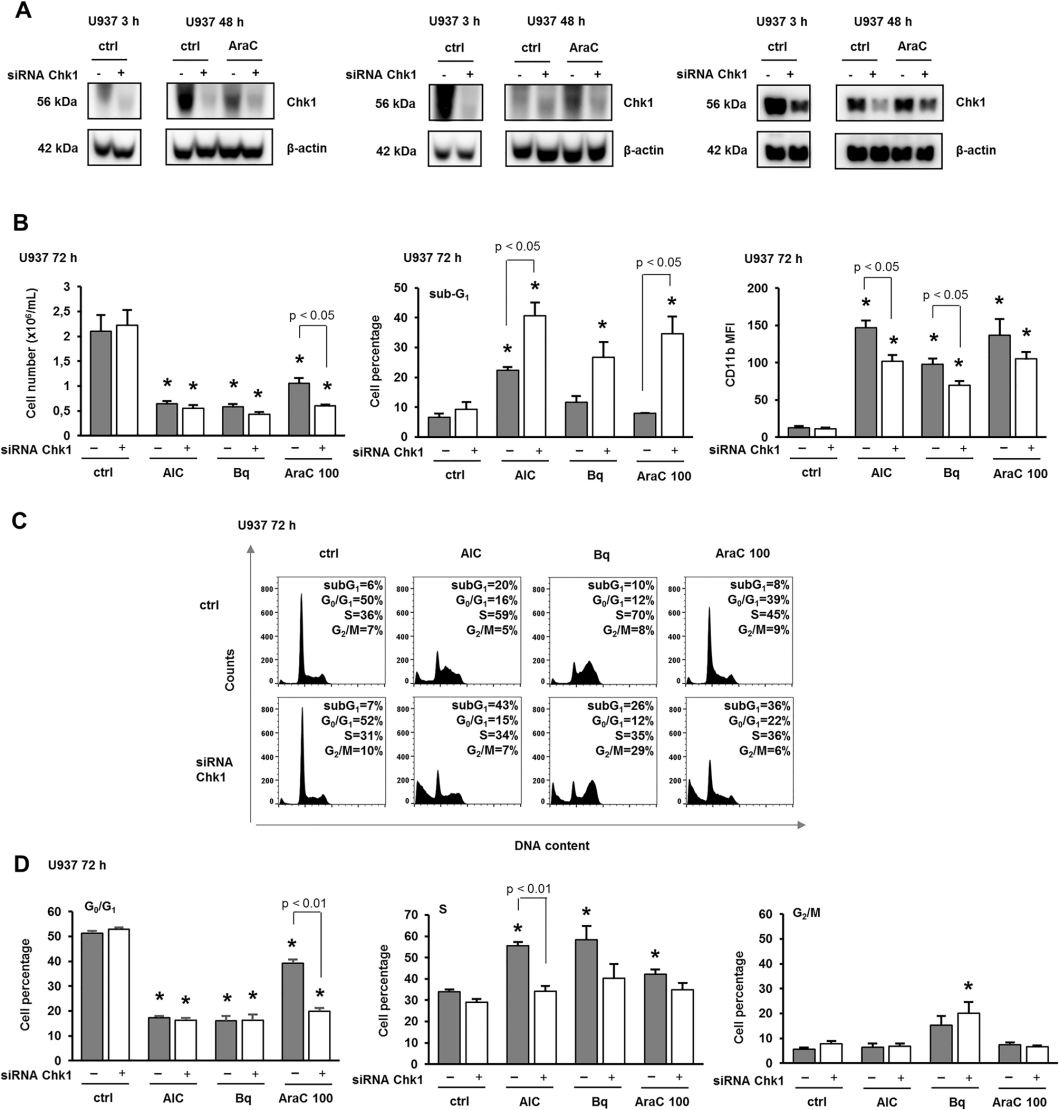
Down-regulation of Chk1 reduces the effects of cytarabine on the expression of differentiation markers and S-phase arrest. U937 cells were transfected with siRNA against CHK1, and respective nontargeting siRNA was used as a negative control. AICAr (AIC) (0.2 mM), brequinar (Bq) (0.5 µM) and AraC (100 nM) were added 24 h after transfection. (**A**) Total cell lysates were isolated 3 or 48 h after the addition of agents and analyzed by Western blotting for the level of Chk1. Western blot analyses are shown for each of the three independent experiments. (**B**) The number of viable cells, the expression of differentiation markers and the cell cycle progression were analyzed by flow cytometry 72 h after addition of agents. (**C**) Representative histograms of propidium-labeled cells analyzed by flow cytometry. (**D**) Percentage of cells in G_0_/G_1_, S, and G_2_/M-phases of the cell cycle. Results are mean ± S.E. (error bars) of at least three independent experiments. *, p < 0.05 (Student's t-test) compared with control (ctrl).

Different effects of Torin2 and VE-821 alone on the progression of the cell cycle indicate that these inhibitors, although widely used as ATR/Chk1 inhibitors, have many off-target effects. Therefore, the effects of 100 nM AraC were tested in U937 cells in which the level of Chk1 was down-regulated by transfection of siRNA targeting Chk1 (Fig. 4A). The results of three independent experiments show that the number of viable cells and the level of CD11b were decreased in AraC-treated cells with down-regulated Chk1 in comparison to cells transfected with control siRNA. The effects were paralleled by a corresponding increase in the percentage of cells exhibiting sub-diploid quantities of DNA (Fig. 4B and Supplementary Fig. 4). The analysis of the cell cycle revealed that the percentage of cells in S-phase was decreased in Chk1-deficient cells treated with all agents. In addition, the percentage of cells in G_0_/G_1_-phase was decreased in Ara-C treated cells transfected with siRNA targeting Chk1 (Fig. 4C and D). These results suggest that the normal level of Chk1 is necessary for AraC-induced differentiation, and that down-regulation of Chk1 reduces differentiation, while promoting a reciprocal increase in AraC-related cell death.

### Cytarabine and pharmacological inhibitors of ATR/Chk1 exert similar effects on proliferation and differentiation in another monocytic cell line

**Figure 5:**
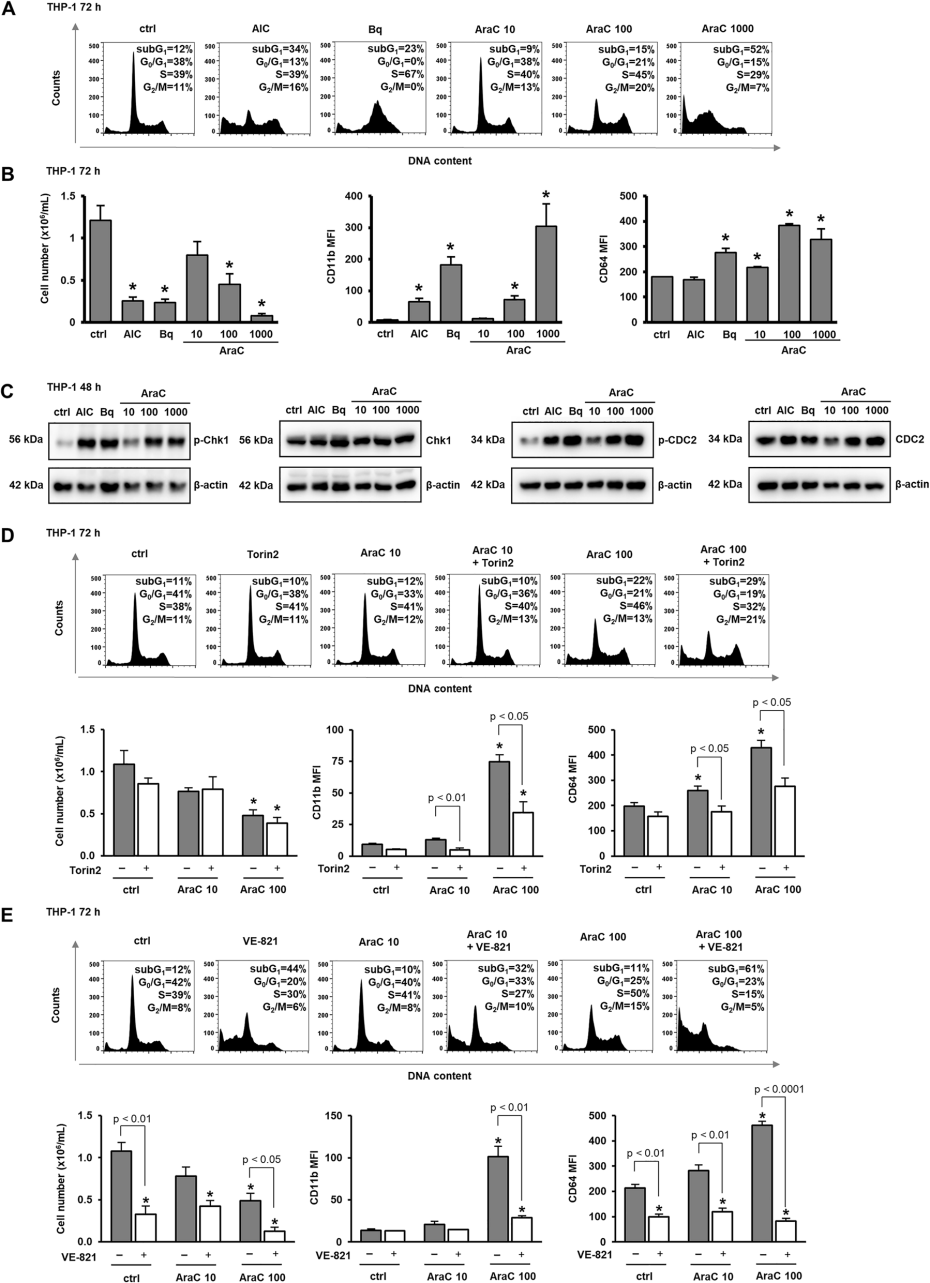
Cytarabine exerts similar effects on proliferation and differentiation in another monocytic cell line. THP-1 cells were incubated with AICAr (AIC) (0.2 mM), brequinar (Bq) (0.5 µM) and AraC (10, 100, 1000 nM) for 72 h. (**A**) Representative histograms of propidium-labelled cells. (**B**) The number of viable cells and the expression of differentiation markers. (**C**) Total cell lysates were isolated after 48 h and analyzed by Western blotting for the level of Ser-345-phosphorylated, total Chk1, Tyr-15-phosphorylated and total CDC2. (**D**) Torin2 (10 nM), (**E**) VE-821 (2 µM) or vehicle (DMSO) were added 30 min before the addition of cytarabine. Histograms and immunoblots are representatives from three independent experiments. The number of viable cells and the expression of differentiation markers were determined as described under “Methods” section. Results are mean ± S.E. (error bars) of at least three independent experiments. *, *p* < 0.05 (Student's t-test) compared with control (ctrl).

We next tested whether key conclusions are applicable for other cell lines. THP-1, a human leukemia monocytic cell line, was treated with increasing doses of AraC and pyrimidine synthesis inhibitors. The analysis of cell cycle distribution revealed that all agents increased the proportion of cells in S-phase, and the highest dose of AraC markedly increased the population of sub-G_1_ (Fig. 5A). An increase in sub-G_1_ correlated with a reduction in number of viable cells, as assessed by trypan blue staining, and was paralleled with an increase in the expression of both CD11b and CD64 (Fig. 5B and Supplementary Fig. 5A). Western blot analysis revealed again an increase in the level of Ser-345-phosphorylated Chk1 and Tyr-15-phosphorylated CDC2 (Fig. 5C), and both Torin2 (Fig. 5D and Supplementary Fig. 5B) and VE-821 (Fig. 5E and Supplementary Fig. 5C) inhibited AraC-mediated increase in the expression of differentiation markers. Therefore, we concluded that AraC-mediated effects are not restricted to U937 cells and we initiated experiments to test whether similar effects would be observed in primary clinical isolates from patients with de novo myeloid malignancies.

### Cytarabine dose-dependently increases expression of differentiation markers in primary samples from AML-M4 and AML-M2 patients that were responsive to pyrimidine synthesis inhibition

Primary blasts isolated from bone marrow samples of five patients with AML were cultured and treated with agents ex vivo, and patients’ characteristics are presented in Supplementary Table 1. These AML samples were previously shown to proliferate in vitro after 72 h. When analyzed for the responsiveness to pyrimidine synthesis inhibitors, two (Pt 07 and Pt 14) out of them have shown a response as measured by an increase in differentiation markers^20^. Therefore, we first tested the effects of increasing doses of AraC on non-APL AML samples that were responsive to AICAr or brequinar. Samples of mononuclear cells isolated from bone marrow after gradient centrifugation and overnight adherence were thawed and plated at a density of 0.4 × 10^6^/mL in a medium supplemented with interleukin-3 (IL-3), interleukin-6 (IL-6), FLT3 ligand (FLT3L) and stem cell factor (SCF) for 72 h.

**Figure 6:**
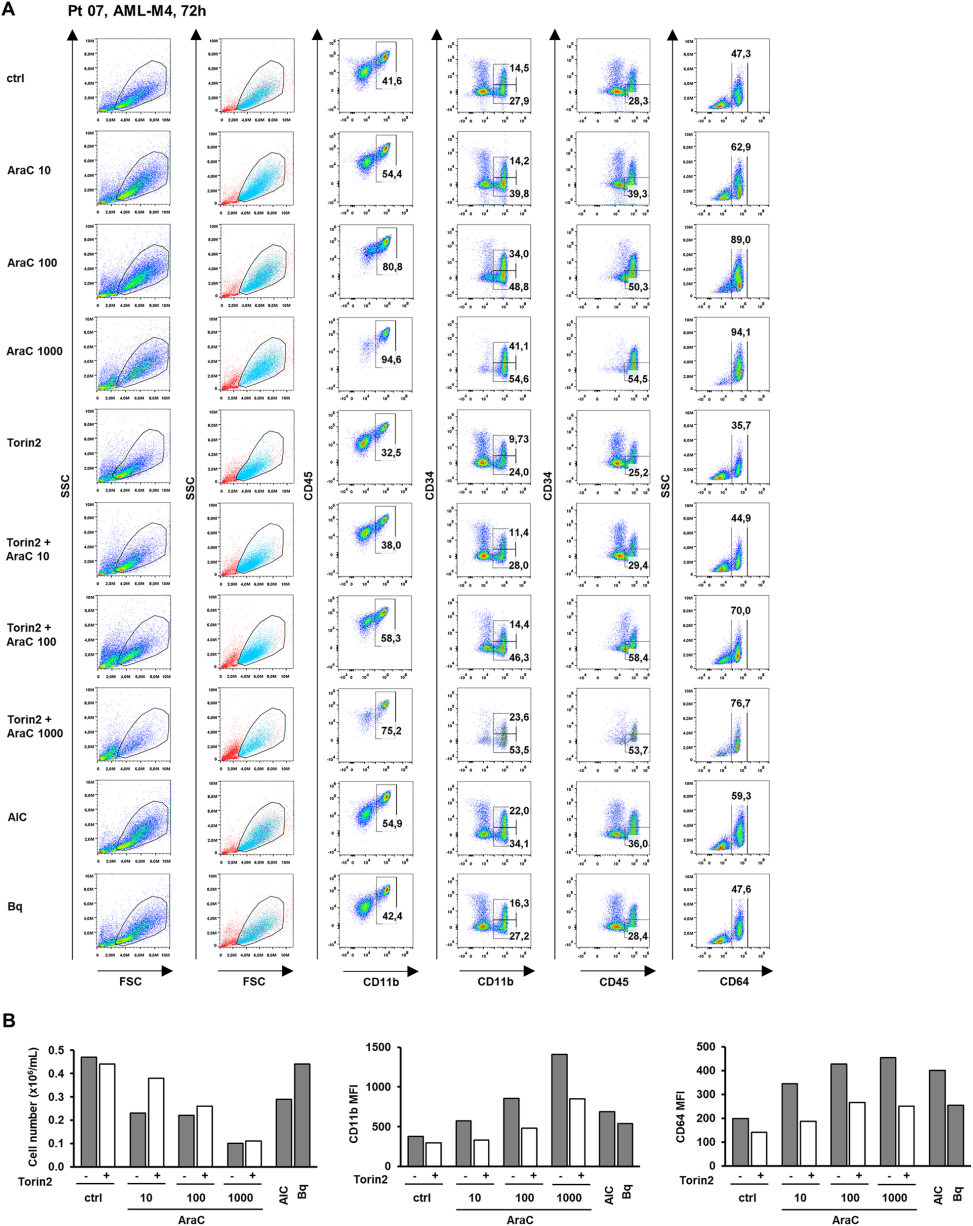
Cytarabine dose-dependently increases expression of differentiation markers in a primary sample from AML-M4 patient that was responsive to pyrimidine synthesis inhibition. Non-adherent mononuclear cells from bone marrow sample (Pt 07, normal karyotype, *FLT3*-ITD, *NPM*/mut) were plated at concentration 0.4 × 10^6^/mL in medium supplemented with 50 ng/mL IL-3, IL-6, SCF and FLT3L and incubated with AraC (10, 100, 1000 nM) with or without Torin2 (10 nM) for 72 h. AICAr (AIC) (0.2 mM) and brequinar (Bq) (0.5 µM) were used as a positive control. Data shown are from a single experiment. (**A**) Flow cytometric analysis of CD11b^+^CD45^+^, CD11b^+^CD34^+^ and CD11b^+^CD34^-^, CD45^high^CD34^−^, and CD64^+^ populations. Percentage of cells in population of interest is indicated in respective gates. Cells within the gate that stained negative for 7-AAD are shown in blue. (**B**) The number of viable cells was determined by trypan blue exclusion. Mean fluorescence intensity (MFI) of CD11b and CD64 was calculated as described under “Methods” section.

As shown in Fig. 6, in a sample of myelomonocytic leukemia cells (Pt 07; FAB-M4, normal karyotype, *FLT3*-ITD, *NPM1*mut), AraC dose-dependently decreased the number of viable cells and increased the expression of CD11b and CD64. In addition, the population of CD45^high^/CD34^-^ cells increased, indicating maturation of blasts. The effect of the lowest dose of AraC (10 nM) was comparable to the effect of AICAr (0.2 mM) and the addition of Torin2 (10 nM) prevented growth arrest and reduced AraC-mediated increase in the expression of markers.

**Figure 7:**
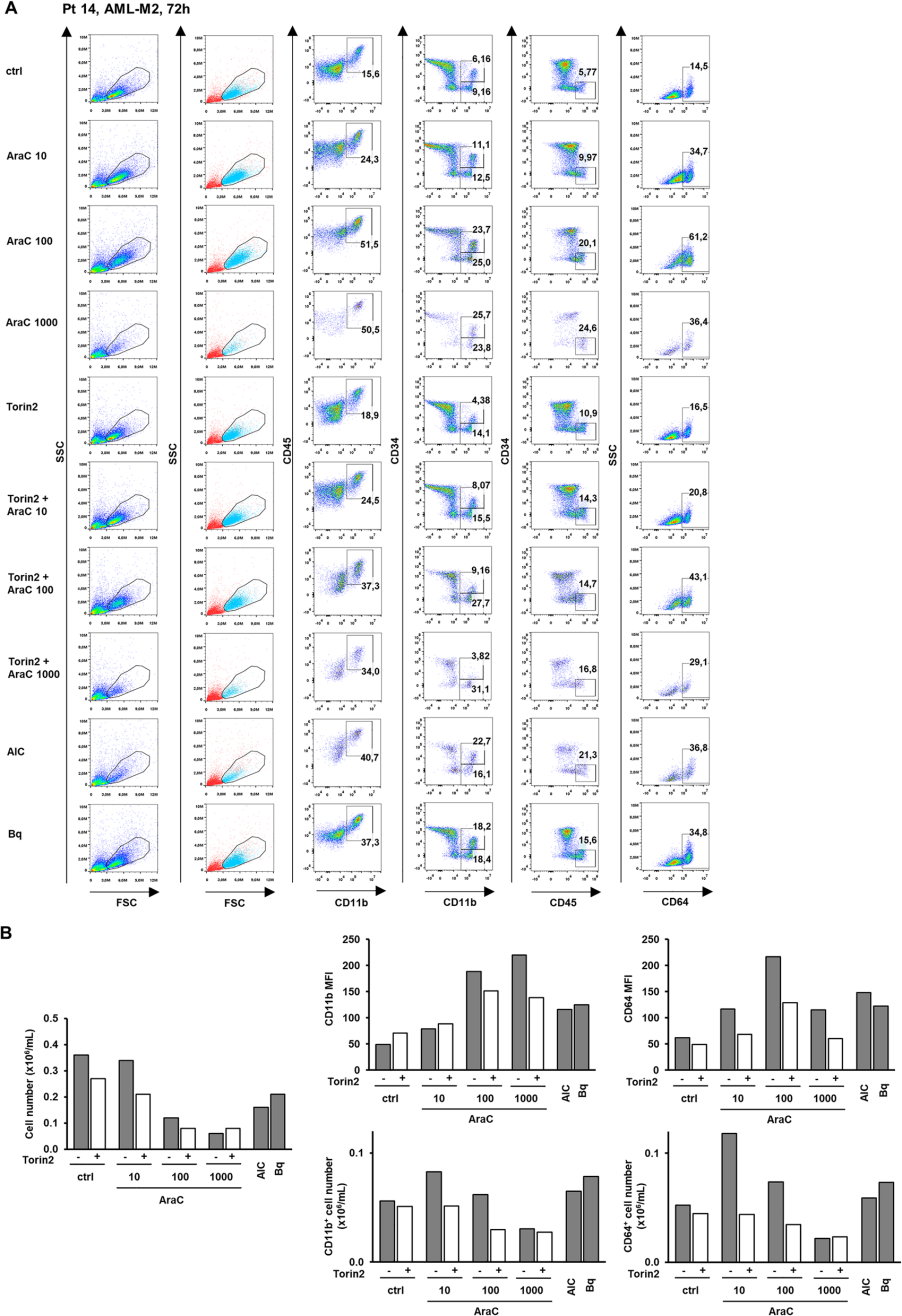
Cytarabine dose-dependently increases expression of differentiation markers in a primary sample from AML-M2 patient that was responsive to pyrimidine synthesis inhibition. Non-adherent mononuclear cells from bone marrow sample (Pt 14, normal karyotype, *FLT3*wt, *NPM*wt) were plated at concentration 0.4 × 10^6^/mL in medium supplemented with 50 ng/mL IL-3, IL-6, SCF and FLT3L and incubated with AraC (10, 100, 1000 nM) with or without Torin2 (10 nM) for 72 h. AICAr (AIC) (0.2 mM) and brequinar (Bq) (0.5 µM) were used as positive controls. **(A)** Flow cytometric analysis of CD11b^+^CD45^+^, CD11b^+^CD34^+^ and CD11b^+^CD34^-^, CD45^high^CD34^-^, and CD64^+^ populations. Percentage of cells in population of interest is indicated in respective gates. Cells within the gate that stained negative for 7-AAD are shown in blue. **(B)** The number of viable cells was determined by trypan blue exclusion. Mean fluorescence intensity (MFI) of CD11b and CD64 was calculated as described under “Methods” section. Absolute number of CD11b^+^ and CD64^+^ cells was calculated from the number of viable cells and the percentage of CD11b^+^CD45^+^ and CD64^+^ cells, respectively.

Similar effects were observed in another sample (Pt 14; FAB-M2, normal karyotype) that responded to both AICAr and brequinar (Fig. 7). The sample was more sensitive to cytotoxic effects of high dose of AraC (1000 nM), but a dose-dependent increase in the percentage of CD11b^+^ and CD64^+^ cells was observed with a parallel decrease in a number of viable cells. Torin2 (10 nM) alone reduced the number of viable cells, but still reduced the expression of differentiation markers, particularly CD64 in response to AraC.

AML cell lines contain homogenous populations and unimodal histograms revealed an increase in differentiation. However, bone marrow biopsy sample contains heterogeneous cell populations, some that are relatively resistant to cytarabine, so that the observed increase in CD11b and CD64 does not necessarily represent differentiation. An alternative explanation to the observed pattern is that more differentiated cells in G_0_ already present in the culture are not killed by cytarabine and therefore increase in frequency but not in absolute number. To rule out the possibility of selection of pre-existing CD11b and CD64 expressing cells, we calculated the absolute number of CD11b and CD64-positive cells in both samples. In a sample of myelomonocytic leukemia cells (Pt 07), no increase in the absolute number of cells was observed (data not shown). However, in FAB-M2 sample (Pt 14) in which low dose cytarabine exerted mild cytotoxicity, an increase in the absolute number of CD11b and CD64-positive cells was observed in samples treated with 10 and 100 nM AraC, AICAr and brequinar, while the number was decreased in the sample treated with the highest dose of AraC (1000 nM) (Fig. 7). These data further corroborated hypothesis that low doses of cytarabine are capable of inducing differentiation of some AML primary samples that are sensitive to pyrimidine synthesis inhibitors.

The effects of increasing doses of AraC were then tested on three samples that have been previously found to be unresponsive to differentiation effects of pyrimidine synthesis inhibition. As shown in Supplementary Fig. 6, the growth of control cells in medium containing cytokines differed between the samples, but no marked increase in differentiation has been observed in any of the samples tested.

### Transcriptional signature in primary AML samples treated with cytarabine resembles transcriptional changes observed after treatment of primary blasts with AICAr or AML cell lines with a DHODH inhibitor

**Figure 8:**
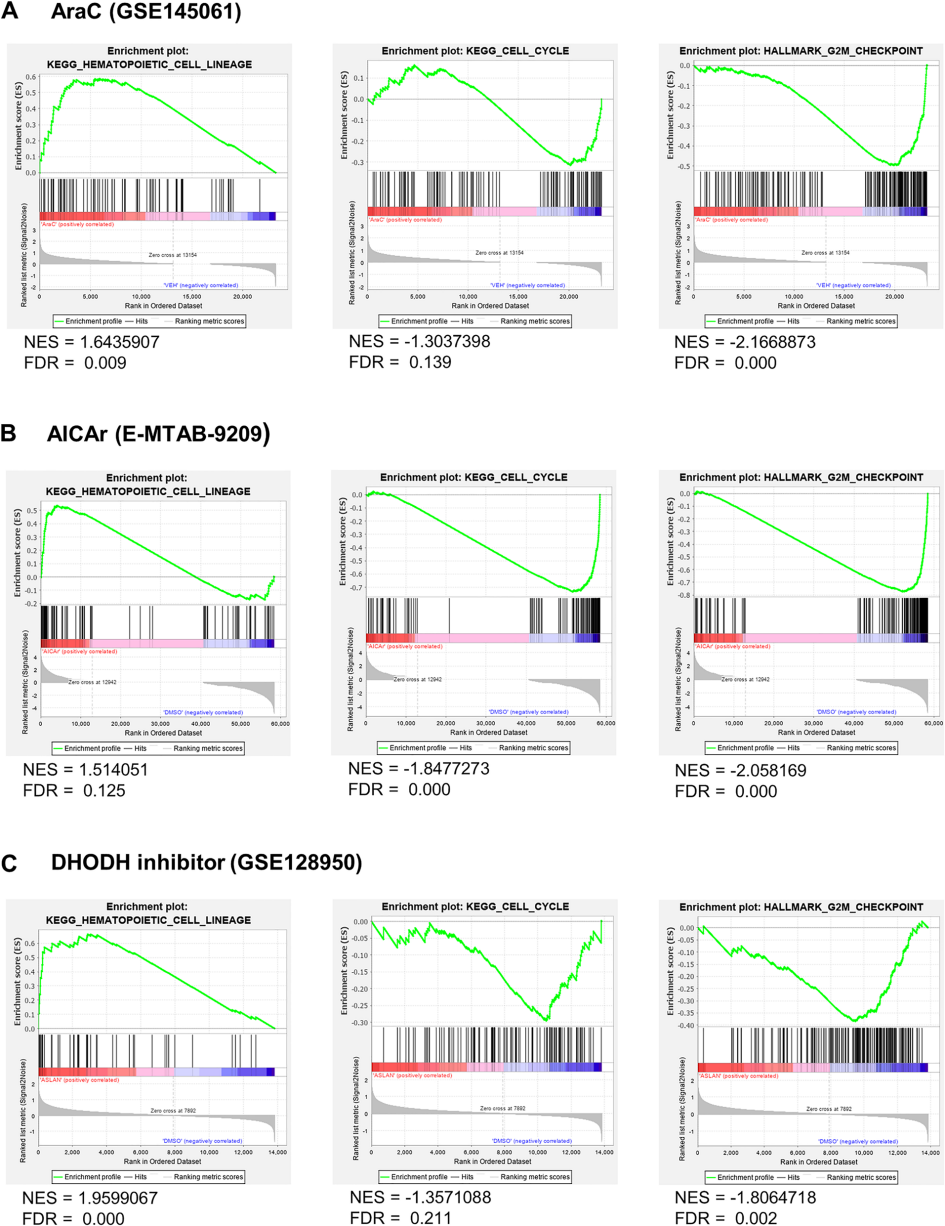
Cytarabine and pyrimidine synthesis inhibitors have similar effects on transcriptional signatures. (**A**) Gene set enrichment analysis of RNASeq read counts from two patient samples treated with 1000 nM AraC in vitro for 24 h. (**B**) Gene set enrichment analysis of RNASeq read counts from AML-M2 patient that was responsive to pyrimidine synthesis inhibition (Pt 14, normal karyotype, *FLT3*wt, *NPM*wt) treated with 0.4 mM AICAr for 24 h. (**C**) Gene set enrichment analysis of RNASeq read counts from MOLM-14 and KG-1 cells treated with a novel DHODH inhibitor ASLAN003 (0.5 and 1 µM) for 24 h (GSE128950). Enrichment plots are shown for KEGG pathways Hematopoietic Cell Lineage and Cell Cycle, and MSigDB G_2_/M Checkpoint pathway.

To compare the changes in gene expression after treatment with AraC and pyrimidine synthesis inhibitors, we analyzed publicly available RNA sequencing dataset (GSE145061) on two patient samples incubated with high dose AraC (1000 nM) for 24 h^21^ and re-analyzed our RNA sequencing data (E-MTAB-9209) on patient sample Pt14 that was responsive to both AICAr and brequinar and incubated with high dose AICAr (0.4 mM) for 24 h^20^. Datasets on primary samples treated with DHODH inhibitors are not currently available so we analyzed a dataset (GSE128950) in which MOLM-14 and KG-1 AML cell lines were treated with a novel DHODH inhibitor ASLAN003 (0.5 and 1 µM respectively) for 96 h^22^. As shown in Fig. 8, gene set enrichment analysis (GSEA) demonstrated similar changes in pathway enrichments in samples treated with AraC (Fig. 8A) and AICAr (Fig. 8B) with both drugs leading to a marked upregulation of the hematopoietic cell lineage genes and down-regulation of the genes in cell cycle pathway and, more narrowly, G_2_/M checkpoint pathway. A similar signature could be observed in AML cell lines MOLM-14 and KG-1 treated with ASLAN003 (Fig. 8C). Although both the dose of AraC used in this dataset is at the high end of the doses we tested, as no datasets with a gradient of AraC concentrations are available, and the DHODH inhibition is performed with a different compound at a different timepoint to the one we used, the GSEA analysis demonstrates strong correlation between biological pathways activated in response to AraC and pyrimidine synthesis inhibitors in primary AML blasts and AML cell lines other than the ones used in this study. These data suggest that the effect we observe in our study is not model-specific and indicate that inhibition of pyrimidine synthesis, irrespective of the pharmacological agent used, differentiates AML cells via the shared pathway involving DNA damage response.

## Discussion

The results of our study suggest that leukemic cell differentiation stimulated by low doses of cytotoxic agents such as AraC depends on the activation of Chk1 and thus shares the same pathway as the inhibitors of pyrimidine synthesis de novo. The activation of DNA damage ATR/Chk1 pathway in response to cytarabine treatment has been previously described in several models of AML, but none of these studies tested the effects on AML differentiation. Results of our study show a dose-dependent increase in Chk1 phosphorylation in response to cytarabine that is similar to the ones previously detected in HL-60 and ML-1 cells^17^, as well as in U937 cells and bone marrow blasts during drug treatment in patients^18^. The role of the pathway has been mostly considered as essential for the maintenance of cell viability by controlling DNA repair and preventing apoptosis and mitotic entry. Therefore, Chk1 activity in response to cytarabine was associated with resistance to apoptosis and several Chk1 inhibitors have been added to cytarabine in order to increase cytotoxicity and overcome resistance. Heat shock protein inhibitors that reduced the level of Chk1^17^, as well as selective Chk1 inhibitors like SCH 900776^18^, GDC-0575^19^, or MK8776^23^ were found to enhance cytarabine-mediated cell killing both in vitro and in vivo. These results are in agreement with the results of our study showing that pharmacological inhibitors and siRNA-mediated down-regulation of Chk1 increased the proportion of cells with subdiploid DNA content when applied to higher doses of cytarabine in vitro. However, results of our study demonstrated that inactivation of Chk1 abrogated cytarabine-mediated differentiation, thus further demonstrating importance of ATR/Chk1 pathway in promoting cellular differentiation in leukemia cells.

There are many studies indicating that DNA damage signaling pathway can lead to other end points besides cell death, including differentiation, growth arrest and senescence. Differentiation induced by activation of DNA damage has been described in retinoic acid-induced differentiation of neuroblastoma cells^24^, melanocytic stem cells differentiation caused by ionizing radiation^25^, and doxorubicin-treated embryonic stem cells^26^. The activation of ATR/Chk1 has been described as important for erythroid maturation since the down-regulation of Chk1 was reported to prevent maturation of K562 cells in response to cytarabine^27^ and pharmacological inhibition of ATR/Chk1 by VE-821 reduced the proportion of mature erythroblasts in response to p53 signaling after ribosome biogenesis arrest^28^. The recent study, which provided RNA sequencing data set on AraC-treated patient samples that we used in our analysis, suggests that the activity of ATR/Chk1 pathway is necessary to induce reversible senescence like-phenotype in AML cells treated with high doses of AraC just before reaching a critical toxic dose that killed these cells^21^**.** More general role of Chk1 in normal hematopoiesis has been recently proposed in a study that confirmed the role of Chk1 in establishing and maintaining fetal and adult hematopoiesis, thus raising concern that treatment strategy aimed to inhibit Chk1 in order to enhance killing by cytotoxic drugs may cause severe myeloablation and even more toxicity^29^. On the other hand, the positive role of activated DNA damage pathway in promoting differentiation of leukemic cells has been described in AML cells in response to ATP depletion^30^ as well as in KMT2A-MLLT3 transformed cells^31^.

Our previous study established the role of ATR/Chk1 pathway in promoting differentiation of several AML cells lines in response to inhibitors of de novo pyrimidine synthesis pathway^15^. In this study, brequinar, a potent and well-known DHODH inhibitor, and AICAr, an inhibitor of UMP synthase, induced S-phase arrest, Chk1 activation and differentiation similar to the one observed in the presence of 100 nM cytarabine, and all of these effects were inhibited by down-regulation of Chk1. Our recent study demonstrated differentiation properties of AICAr and brequinar in primary AML blasts^20^, and the present study shows similar effects of cytarabine in primary samples that were responsive to brequinar and AICAr, further corroborating hypothesis that cytarabine-mediated differentiation shares the same mechanism of action. DHODH inhibitors are currently considered a promising option for differentiation therapy of AML^2^ and, although clinical application of brequinar is limited due to severe adverse reactions, several novel inhibitors of DHODH are developed that exerted the similar effects on AML differentiation, including BAY 240234^32^ and ASLAN003^22^. AICAr has been known to exert antiproliferative effects in hematological malignancies in vitro, and two clinical trials tested the effects of higher doses of AICAr in patients with B cell chronic lymphocytic leukemia^33^, and refractory MDS/AML patients^34^, but the effects of low doses of AICAr on differentiation of AML cells have not been tested in vivo^35^. The results of the present study show that no matter whether replication stall is induced by lack of nucleotides or addition of AraC, the activity of ATR/Chk1 increases to promote growth arrest and differentiation, thus suggesting that low doses of cytarabine may have the same effect as pyrimidine synthesis inhibitors.

The precise mechanism linking the activation of Chk1 and leukemia differentiation is not clear. Recent study described an increase in autophagy in AML cells treated with low doses of AraC^14^, which is similar to an increase in autophagy we observed in cells treated with AICAr and ATRA, but our study demonstrated that down-regulation of key proteins of classical autophagy pathway had no effects on AML cells differentiation^36^. In the present study, activation of Chk1 is followed by inhibitory phosphorylation of CDK1 and it has been reported that pharmacologic and genetic inhibition of CDK1^37^ or knock-down of upstream *CDC25A*^38^ induces differentiation through C/EBPα in AML cells with activated FLT3. However, no such effects were observed in U937 cells^37^, and we observed differentiation in AML cell lines and primary samples lacking *FLT3-*ITD. THP-1 cells used in our study are *KMT2A-MLLT3*^pos^ cells, and CDK6 was demonstrated to be important for differentiation in *KMT2A*-rearranged AML cells, but genetic down-regulation of CDK6 in study by Placke et al.^39^ induced mild G_0_/G_1_ arrest associated with differentiation, while we observed an arrest of THP-1 cells in S and G_2_/M-phase. Both U937 and THP-1 cells lack functional p53^40^, and p53-mediated response to DNA damage is known to affect chemotherapy response in AML^41,42^. Down-regulation of several other cell cycle regulators, like CDK2^43^ or AURKA^44^ were found to induce differentiation, as well as upregulation of p21^13,31^ or accumulation of PU.1 transcription factors^45^.

The results of our study on two AML cell lines and primary samples are in line with previous studies demonstrating that doses up to 100 nM AraC provide an effective approach for inducing differentiation of leukemia cells in vitro, while higher concentrations exert an increasing cytotoxicity^13,14^. The tested cytarabine concentrations compare to concentrations observed in plasma of patients receiving 20 mg/m^2^/d by continuous IV infusion in which similar plasma AraC levels (10–100 nM) were measured at week 1 and week 2^46^. Further investigations would be necessary to determine non-APL AML patients who could benefit from AraC-mediated differentiation therapy, but results of our study suggest that the same group may be equally sensitive to therapy aimed to differentiate AML cells by blocking pyrimidine synthesis de novo. Our recent study identified non-APL AML blasts having an inversion of chromosome 16 and the presence of *CBFB-MYH11* fusion transcripts as sensitive to ATRA in vitro^40^, but no such conventional diagnostic markers can be used to identify low dose AraC sensitive patients.

In summary, this study revealed that LDAC induces differentiation through activation of Chk1 signaling pathway and thus shares the same mechanism as pyrimidine synthesis inhibitors. Since the effects of DHODH inhibitors are currently under investigation in several clinical trials, we think that the results of our study may help to define relapsed/refractory patients who may benefit from inhibitors of pyrimidine synthesis. In addition, understanding the mechanism of differentiation effects of LDAC may help to instruct more rationale-based therapeutic approaches in older patients who are ineligible for intensive chemotherapy.

## Methods

### Reagents

Reagents used are listed in Supplementary Table 2.

### Cell lines and primary AML samples cell culture

Human AML cell lines U937 (ECACC Cat# 85,011,440) and THP-1 (DSMZ Cat# ACC-16) were purchased from European Collection of Animal Cell Cultures (Porton, Salisbury, UK) or Leibniz Institute-Deutsche Sammlung von Mikroorganismen und Zellkulturen GmbH (Braunschweig, Germany), respectively. The cell lines were maintained in RPMI 1640 medium containing 10% FBS, 2 mM L-glutamine, 50 U/mL penicillin and 50 μg/mL streptomycin at 37 °C in a humidified atmosphere containing 5% CO_2_. Exponentially growing cells were collected, the viability was determined by trypan blue exclusion, and viable cells were seeded at starting concentration of 0.2 × 10^6^/mL in 6-well plates or 0.3 × 10^6^/mL in 25-cm^2^ flasks. The agents tested were added at concentrations indicated in the figure legends.

Bone marrow samples were obtained upon written informed consent from five patients with non-APL AML. The study was performed according to the Declaration of Helsinki and approved by the Institutional Review Board of the University of Zagreb School of Medicine (380-59-10,106-17-100/94) and University Hospital Centre Zagreb (02/21 AG). In routine diagnostic procedures, the samples were evaluated for the presence of *FLT3* mutations and cytogenetic abnormalities. Samples with normal karyotypes were additionally tested for *NPM1* mutations. Further analyses of *BCR-ABL, RUNX1/RUNX1T1, PML-RARA, CBFB/MYH11* and *KMT2A-AFF1/MLLT3* were performed depending on the French-American-British (FAB) subtype. AML patients’ characteristics are presented in Supplementary Table 1.

Diagnostic bone marrow aspirates were separated using NycoPrep™ 1.077. Mononuclear cells from bone marrow samples 03 and 14 were immediately frozen, while cells from samples 07, 13 and 15 were further purified by overnight adherence to plastic and then cryopreserved in liquid nitrogen. For the experiments, samples were thawed, samples 03 and 14 were allowed to adhere to plastic overnight, and non-adherent cells in all samples were resuspended in RPMI 1640 containing 10% FBS, 2 mM L-glutamine, 50 U/mL penicillin and 50 μg/mL streptomycin. Total cell number and viability were assessed by counting on hemocytometer using trypan blue exclusion. Viable cells were seeded at the concentration of 0.4 × 10^6^/mL in 12-well plates, in medium supplemented with 50 ng/mL interleukin-3 (IL-3), interleukin-6 (IL-6), FLT3 ligand (FLT3L) and stem cell factor (SCF), as previously described^20^, and treated with agents tested.

Total number of viable cells was assessed by counting on hemocytometer using trypan blue exclusion.

### Expression of surface markers and cell cycle analysis

Cultured cell lines and primary blasts were stained with appropriate antibodies as previously described^15,20^. Non-viable cells were excluded by 7-AAD staining and forward vs side scatter gating, and doublet exclusion was performed by plotting the height against the area for forward scatter. For cell cycle analysis, an aliquot of cells was stained directly with propidium iodide (PI) solution as previously described^15^. Flow cytometry analyses were performed using Attune acoustic focusing cytometer (Life Technologies, ABI, Carlsbad, CA, USA) and the obtained data were analyzed by FlowJo v.10 platform (TreeStar, Ashland, OR, USA). Mean fluorescence intensity (MFI) of the sample was calculated by subtracting MFI levels of isotypic controls from MFI levels of the cells stained with CD-specific antibodies. Percentage of positive cells was determined by measuring the fluorescence shift of distinct cluster of leukemic events. For cell cycle analysis, data were processed using doublet discrimination and automatic assignment of cell cycle boundaries according to the propidium iodide intensity using FlowJo cell cycle platform.

### Respiratory burst

U937 cells were first incubated with 100 μM dihydrorhodamine 123 (DHR123) for 5 min at 37 ^0^C in shaking water bath and then activated with 0.4 μg/ml PMA for 15 min. Flow cytometry analyses were performed using the FACSLyric (BD Biosciences, San Jose, CA, USA) and MFI of the sample was determined by FlowJo v.10 platform.

### Annexin V-FITC staining

Apoptosis was measured as the percentage of annexin V-FITC- and PI- positive cells, according to manufacturer's instructions. Samples were analyzed using the FACSLyric system (BD Biosciences, San Jose, CA, USA) and FlowJo v.10 platform.

### Western blot

Total cell lysates were isolated as previously described^15^. Equal amounts of protein extracts (50 μg/well) were run on SDS–polyacrylamide gel and transferred to nitrocellulose membrane using Mini-PROTEAN Tetra electrophoresis system (Bio-Rad Laboratories, Hercules, CA, USA). Anti-Chk1, anti-pChk1, anti-cell-division cycle 2 (CDC2), anti-pCDC2 and anti-actin primary antibodies were employed at the recommended dilutions. Protein bands were visualized using SuperSignal™ West Pico PLUS chemiluminescent substrate, ChemiDoc™ MP Imaging System (Bio-Rad Laboratories, Hercules, CA, USA) and Image Lab™ software (Bio-Rad, California, USA).

### siRNA transfection

Cells were transfected with siRNA against CHK1 and corresponding control using Neon transfection system (Life Technologies, Carlsbad, CA, USA) as previously described^15,36^.

### Morphological analysis

Samples were cytospun on microscopic slides (1000 rpm, 2 min) using StatSpin Cytofuge 2 (BeckmanCoulter, Marseille, France) and left to dry overnight. Slides were stained with May-Grünwald stain (50% working solution, 5 min) and, subsequently, Giemsa stain (10% working solution, 20 min). Morphology was examined using AxioVert 200 microscope and images were obtained using AxioCam MRc 5 camera and ZEN software, blue edition (Carl Zeiss AG, Oberkochen, Germany).

### Gene expression in AML cells and GSEA analysis

Gene expression data on two primary AML samples treated with 1000 nM AraC in vitro for 24 h were downloaded from GEO database, series GSE145061. Patient characteristics^21^ are described in Supplementary Table 3. Gene expression data on patient sample from our biobank (Pt 14) treated with 0.4 mM AICAr for 24 h are accessible from ArrayExpress database, series E-MTAB-9209. Additionally, gene expression data on AML cell lines treated with a DHODH inhibitor ASLAN003^22^ correspond to GEO database series GSE128950. Gene set enrichment analysis was performed using GSEA software (http://www.broadinstitute.org/gsea/) and R software and it was based on KEGG Pathway Database and curated gene sets from the Broad Institute’s molecular signatures database MSigDB^47^. FDR < 0.25 was considered significant.

#### Statistical analysis

Data are presented as mean ± standard error of the mean (S.E.M). Statistical analysis was performed using Microsoft Excel to calculate the p values for Student’s t test. The results were considered to be statistically significant if *p* was < 0.05.

## Supplementary Information

Supplementary Information 1.

Supplementary Information 2.

## Data Availability

Gene expression data are accessible from: GEO database, series GSE145061; ArrayExpress database, series E-MTAB-9209; GEO database series GSE128950. All other datasets generated and/or analyzed during the current study are available from authors on request.
