# Supplementary material for: Cytarabine-induced differentiation of AML cells depends on Chk1 activation and shares the mechanism with inhibitors of DHODH and pyrimidine synthesis
Source: Sci Rep. 2022 Jul 5;12:11344. doi: 10.1038/s41598-022-15520-z (PMC9256737; doi:10.1038/s41598-022-15520-z)
Supplement: Supplementary file 1 — Supplementary Information 1. [file 41598_2022_15520_MOESM1_ESM.pdf]

Western blot original files

REVISED

For Figure 2.

U937 48 h

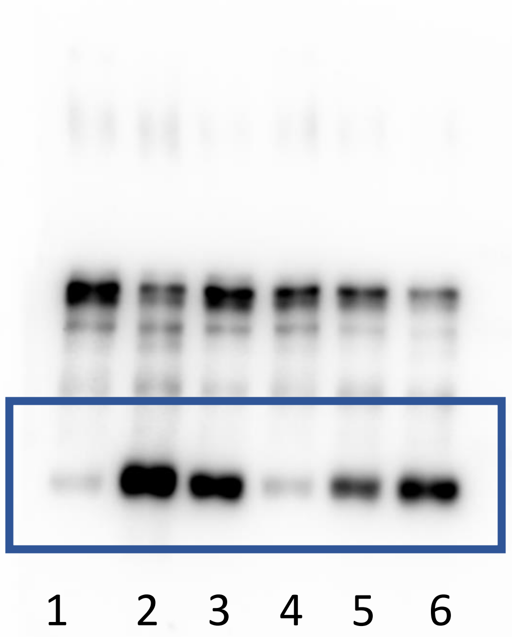

Fig.2.D WB1 p-Chk1

Lane no.	1	2	3	4	5	6
Sample (U937 48 h)	ctrl	AIC	Bq	AraC 10	AraC 100	AraC 1000

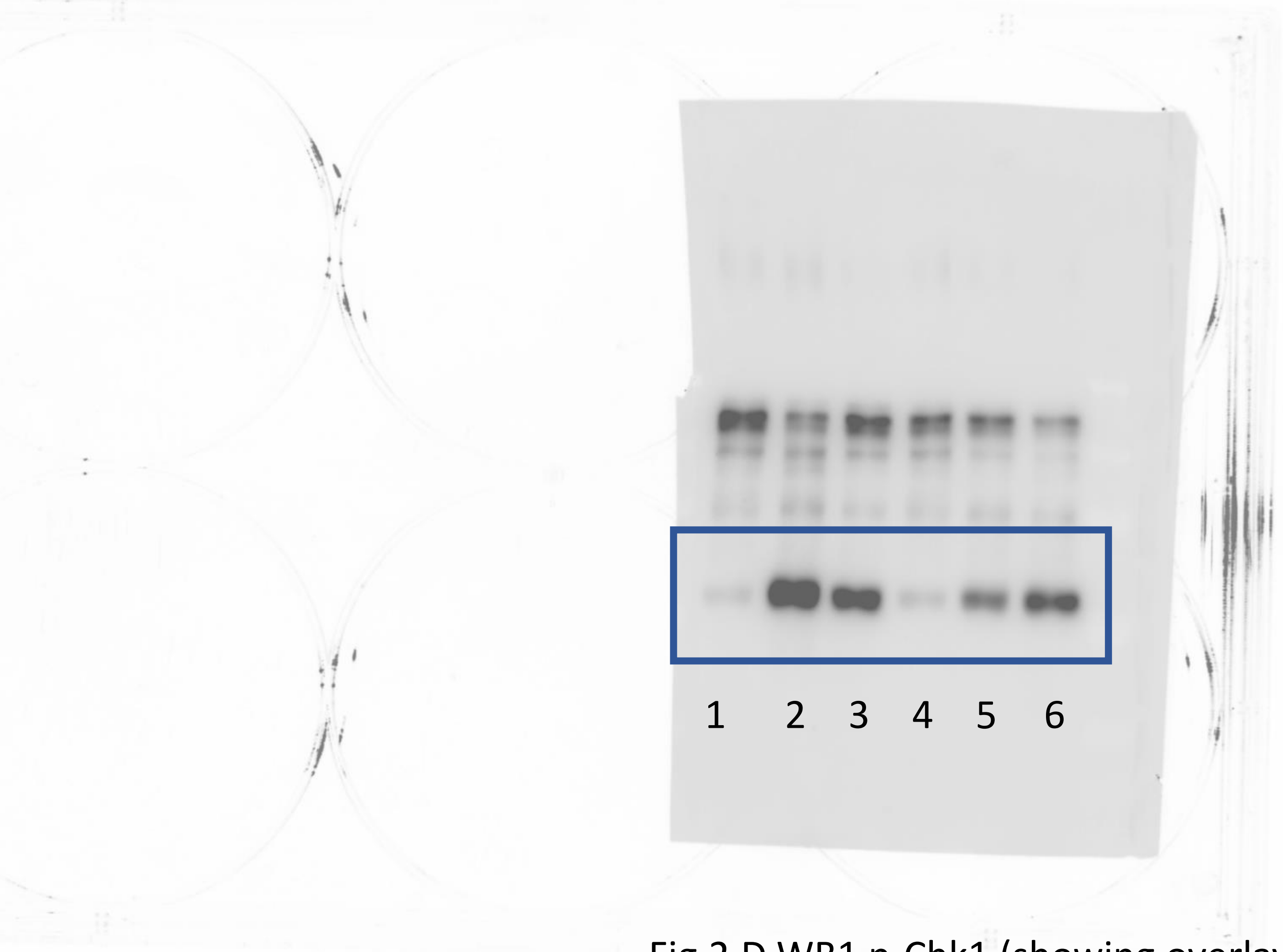

Fig.2.D WB1 p-Chk1 (showing overlay)

Lane no.	1	2	3	4	5	6
Sample (U937 48 h)	ctrl	AIC	Bq	AraC 10	AraC 100	AraC 1000

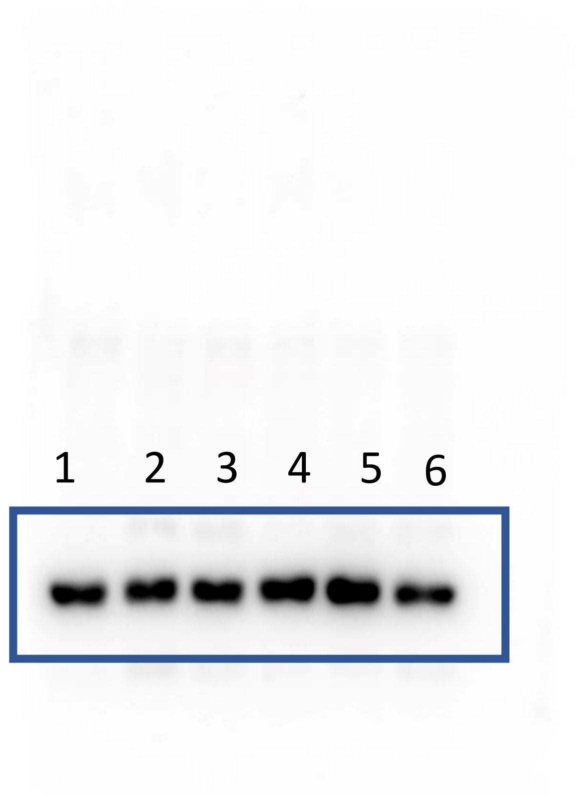

Fig.2.D WB1 beta-actin

Lane no.	1	2	3	4	5	6
Sample (U937 48 h)	ctrl	AIC	Bq	AraC 10	AraC 100	AraC 1000

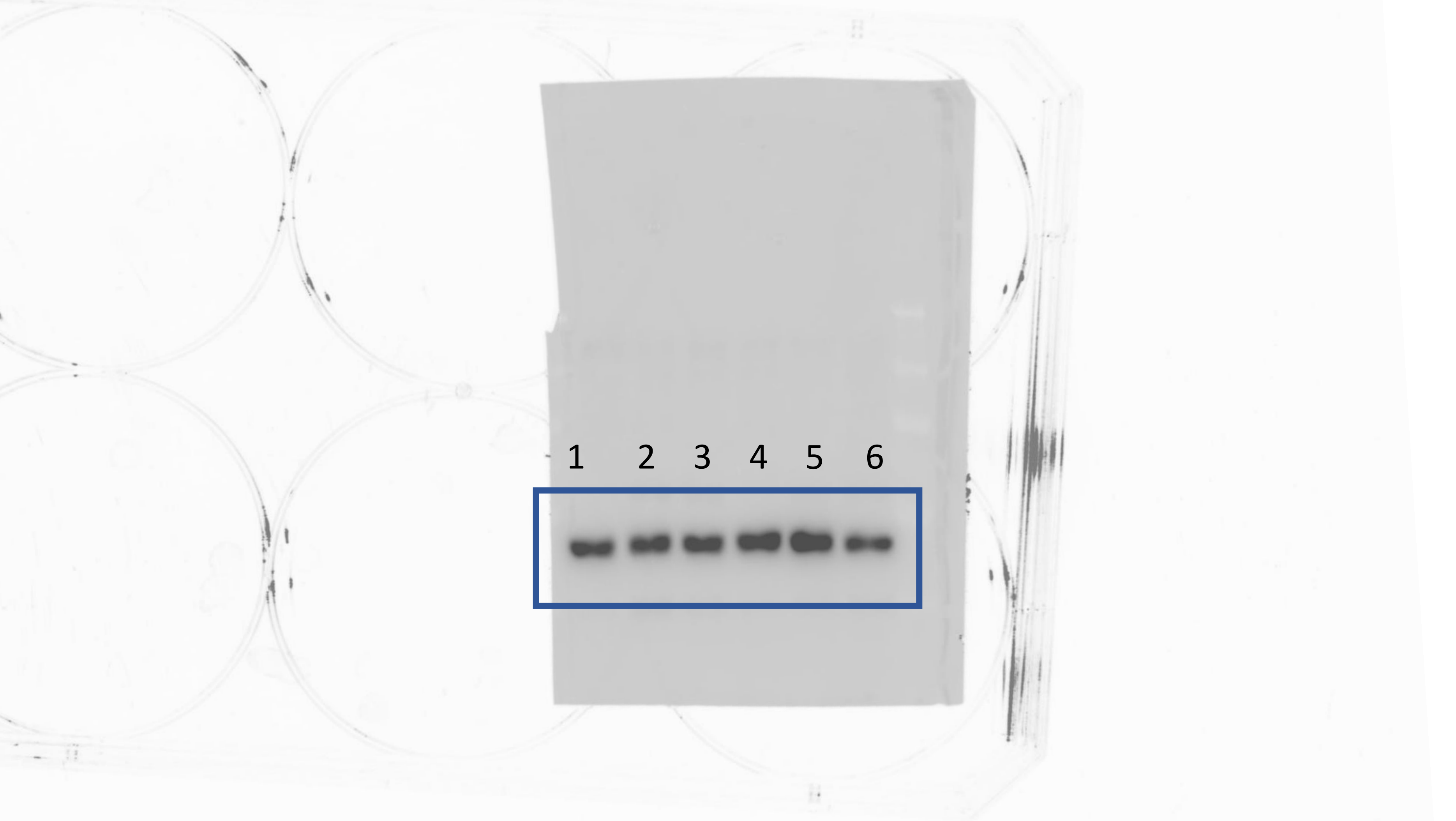

Fig.2.D WB1 beta-actin (showing overlay)

Lane no.	1	2	3	4	5	6
Sample (U937 48 h)	ctrl	AIC	Bq	AraC 10	AraC 100	AraC 1000

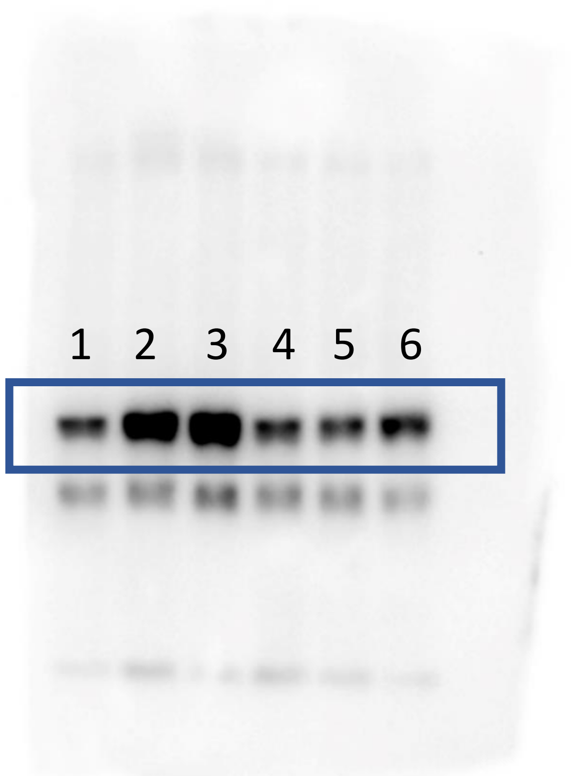

Fig.2.D WB2 Chk1

Lane no.	1	2	3	4	5	6
Sample (U937 48 h)	ctrl	AIC	Bq	AraC 10	AraC 100	AraC 1000

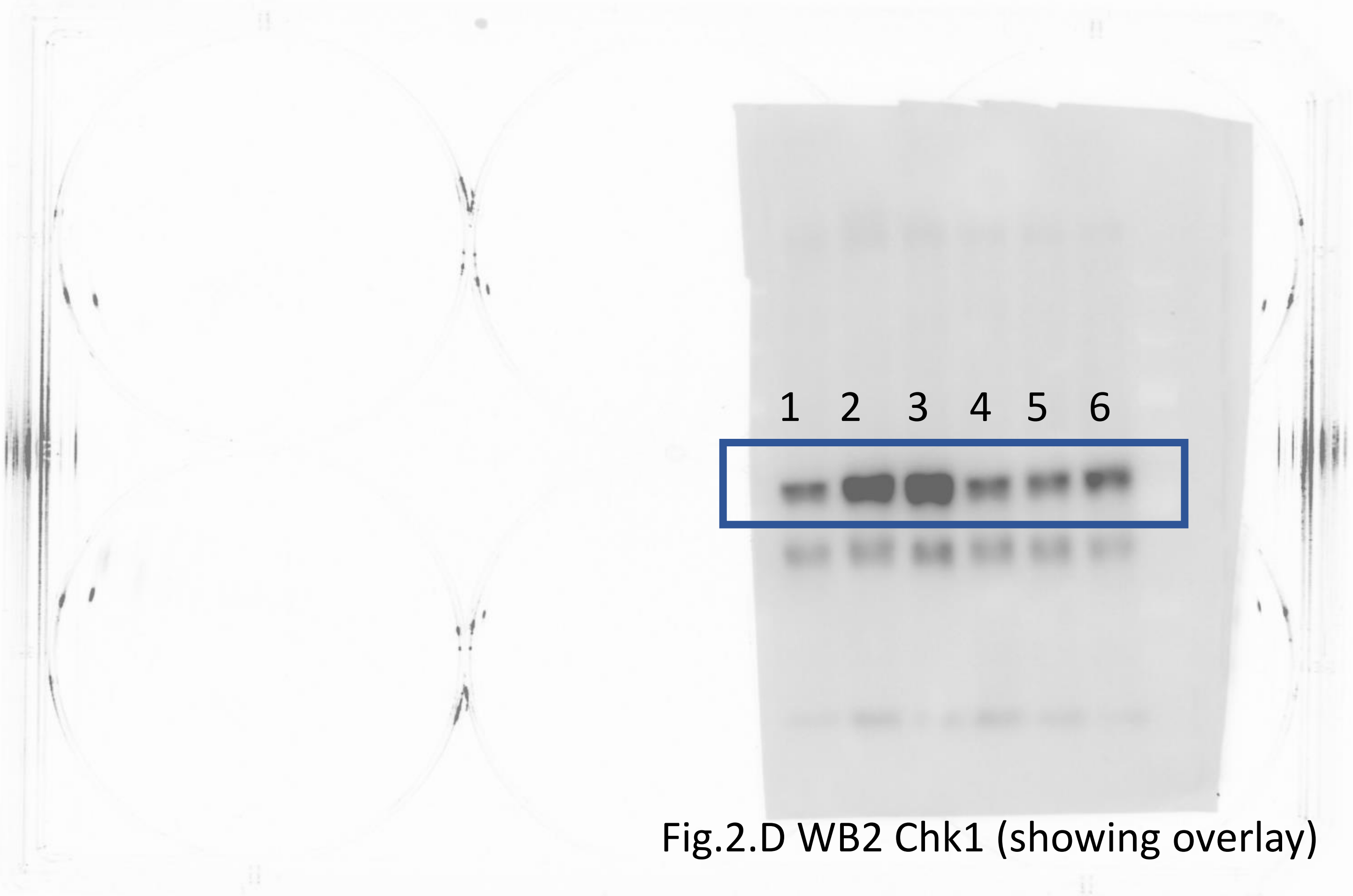

Fig.2.D WB2 Chk1 (showing overlay)

Lane no.	1	2	3	4	5	6
Sample (U937 48 h)	ctrl	AIC	Bq	AraC 10	AraC 100	AraC 1000

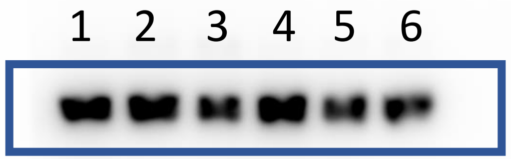

Fig.2.D WB2 beta-actin

Lane no.	1	2	3	4	5	6
Sample (U937 48 h)	ctrl	AIC	Bq	AraC 10	AraC 100	AraC 1000

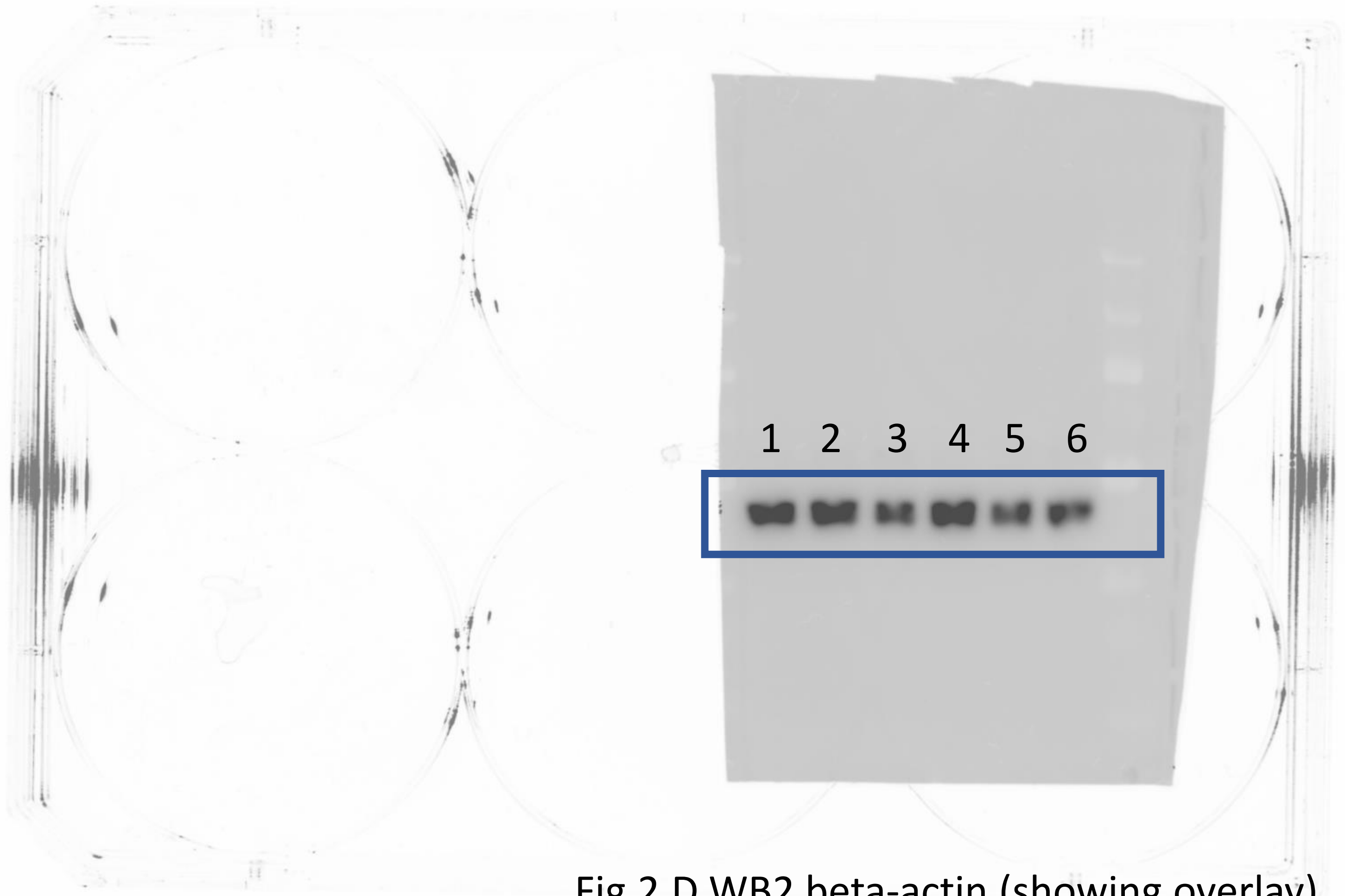

Fig.2.D WB2 beta-actin (showing overlay)

Lane no.	1	2	3	4	5	6
Sample (U937 48 h)	ctrl	AIC	Bq	AraC 10	AraC 100	AraC 1000

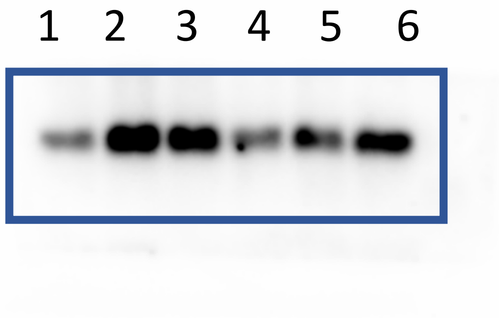

Fig.2.D WB3 p-CDC2

Lane no.	1	2	3	4	5	6
Sample (U937 48 h)	ctrl	AIC	Bq	AraC 10	AraC 100	AraC 1000

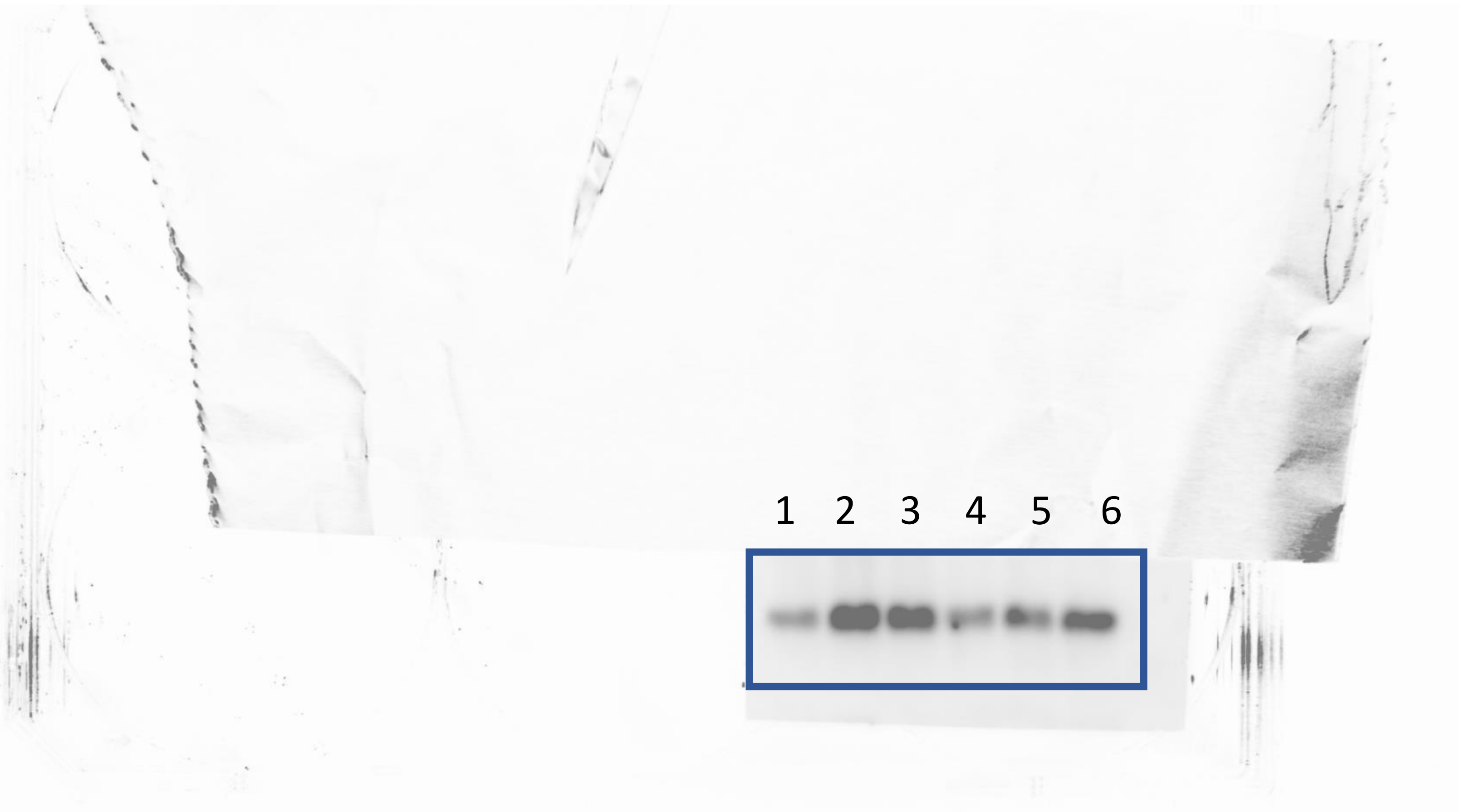

Fig.2.D WB3 p-CDC2 (showing overlay)

Lane no.	1	2	3	4	5	6
Sample (U937 48 h)	ctrl	AIC	Bq	AraC 10	AraC 100	AraC 1000

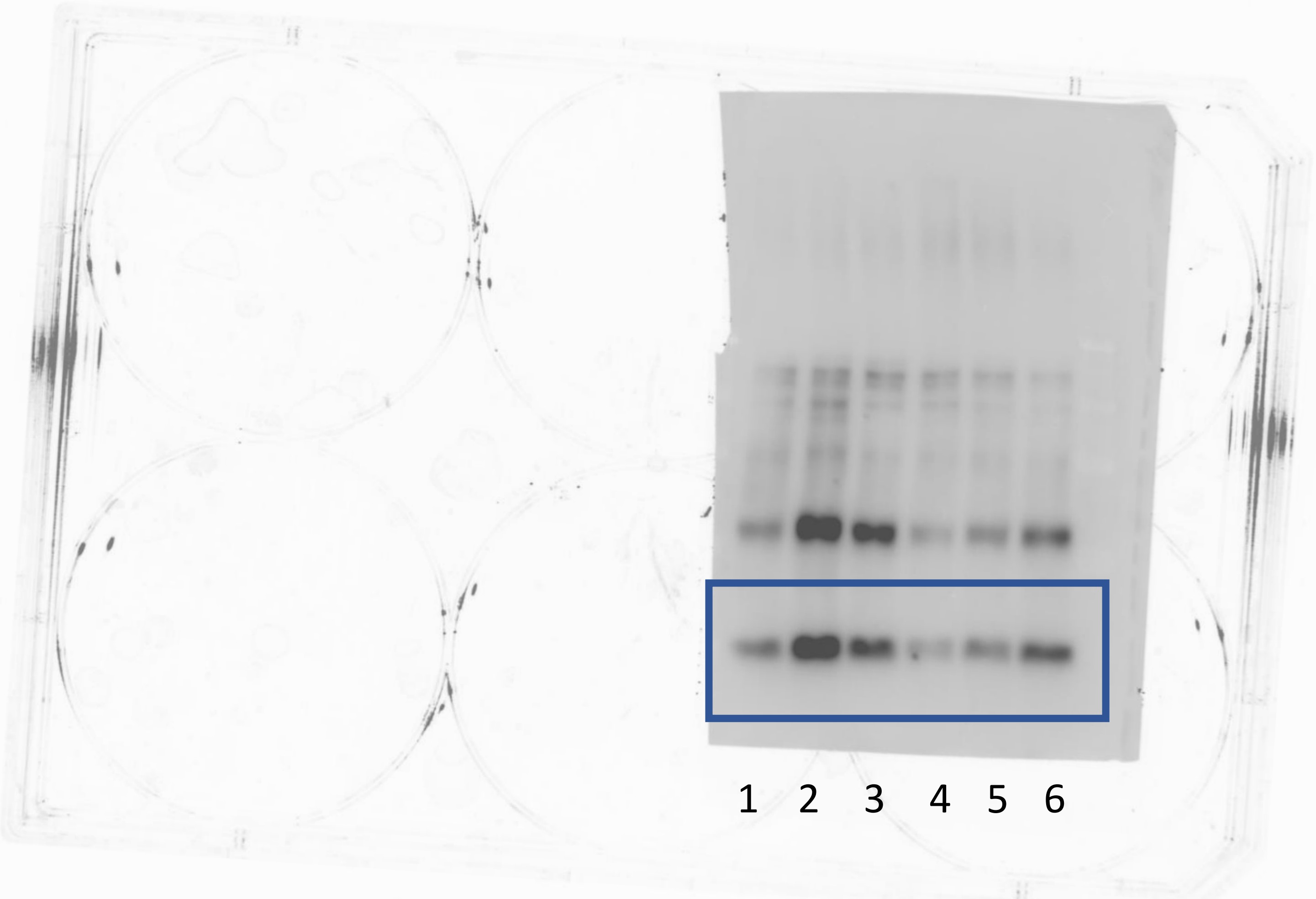

Fig.2.D WB3 p-CDC2 (showing overlay of the whole membrane)

Lane no.	1	2	3	4	5	6
Sample (U937 48 h)	ctrl	AIC	Bq	AraC 10	AraC 100	AraC 1000

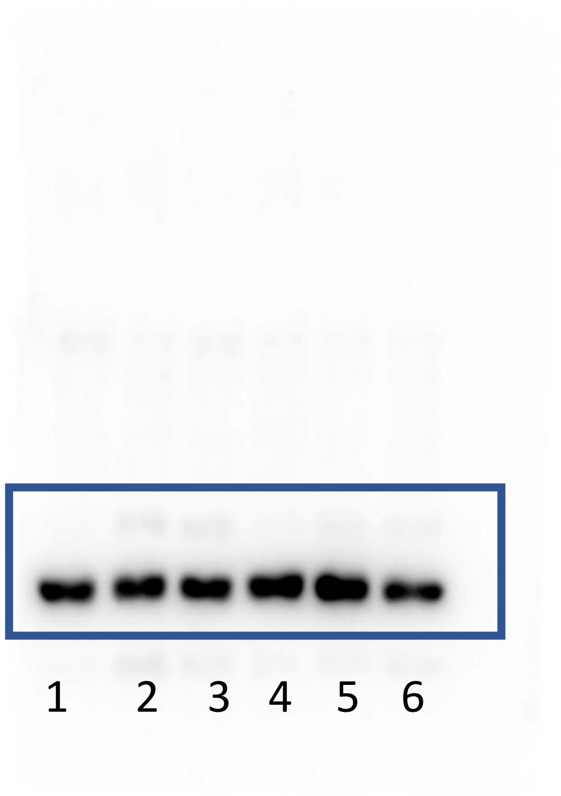

Fig.2.D WB3 beta-actin

Lane no.	1	2	3	4	5	6
Sample (U937 48 h)	ctrl	AIC	Bq	AraC 10	AraC 100	AraC 1000

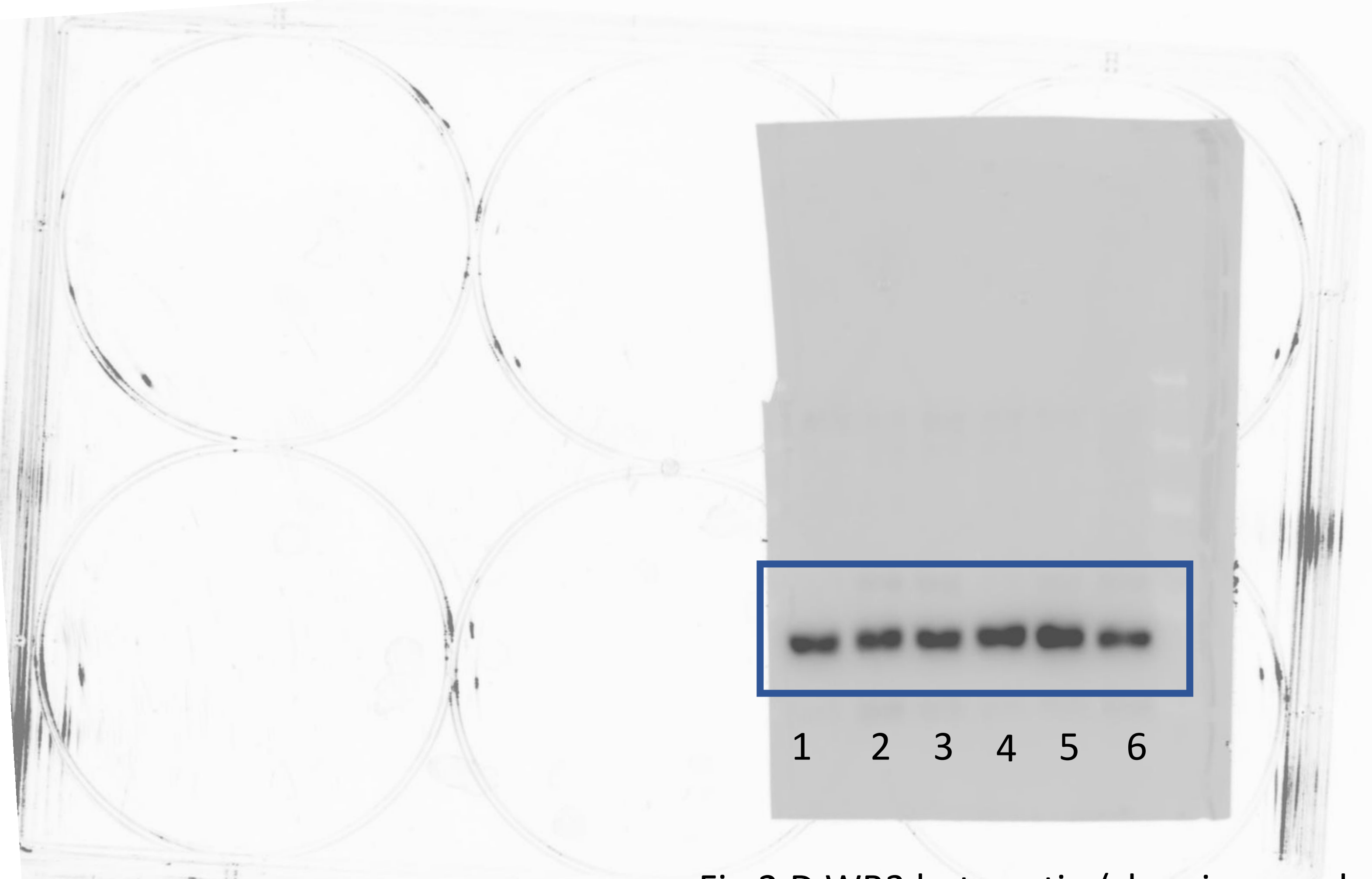

Fig.2.D WB3 beta-actin (showing overlay)

Lane no.	1	2	3	4	5	6
Sample (U937 48 h)	ctrl	AIC	Bq	AraC 10	AraC 100	AraC 1000

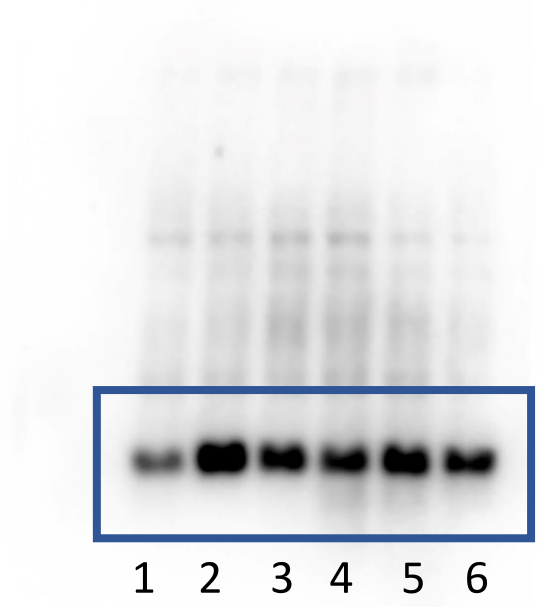

Fig.2.D WB4 CDC2

Lane no.	1	2	3	4	5	6
Sample (U937 48 h)	ctrl	AIC	Bq	AraC 10	AraC 100	AraC 1000

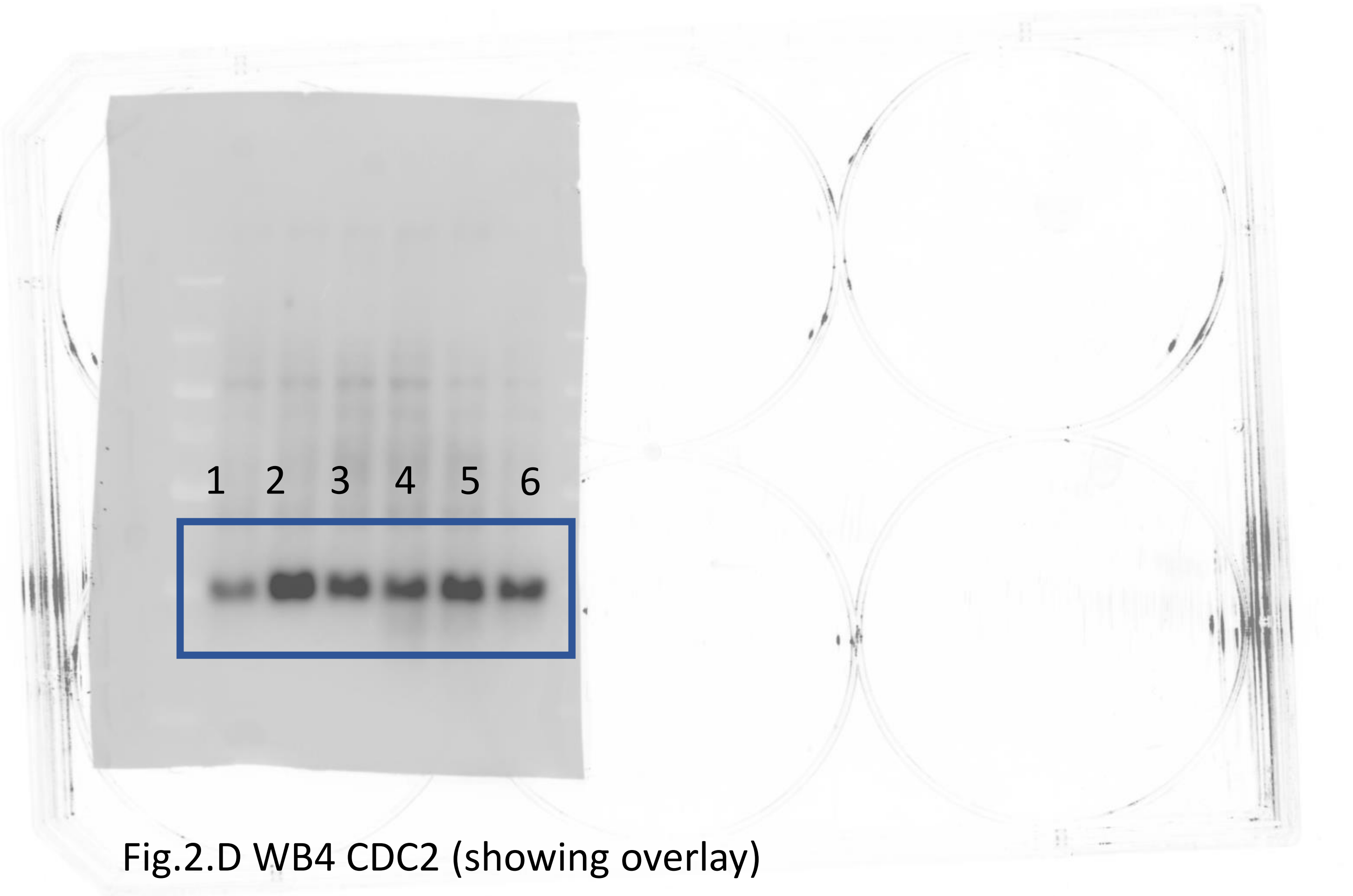

Fig.2.D WB4 CDC2 (showing overlay)

Lane no.	1	2	3	4	5	6
Sample (U937 48 h)	ctrl	AIC	Bq	AraC 10	AraC 100	AraC 1000

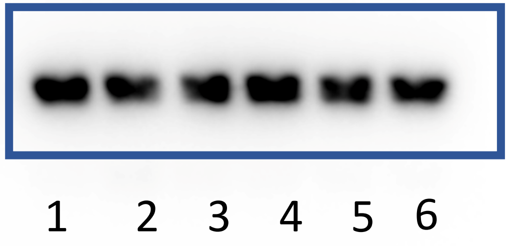

Fig.2.D WB4 beta-actin

Lane no.	1	2	3	4	5	6
Sample (U937 48 h)	ctrl	AIC	Bq	AraC 10	AraC 100	AraC 1000

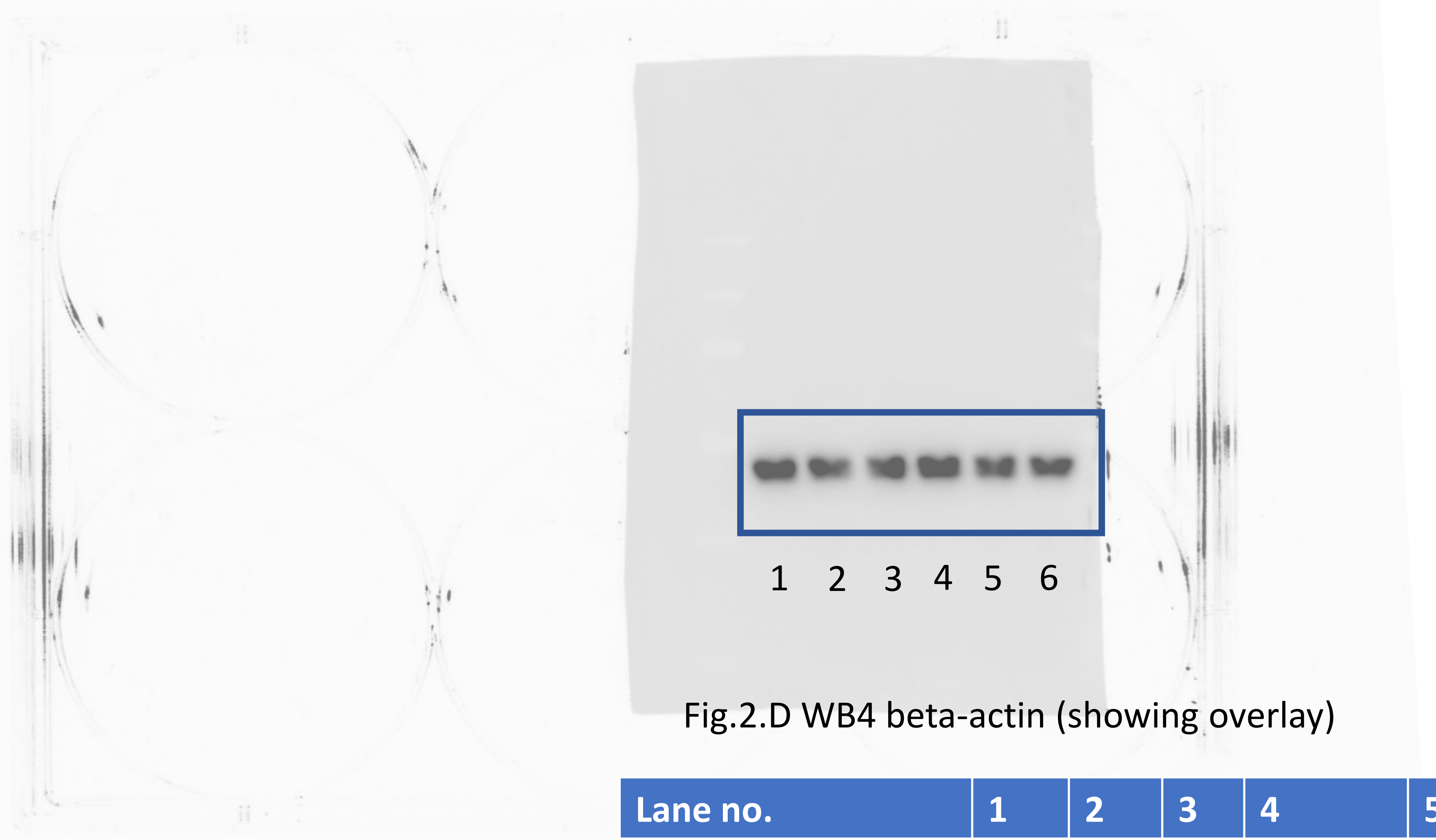

Fig.2.D WB4 beta-actin (showing overlay)

**Lane no.**	**1**	**2**	**3**	**4**	**5**	**6**
Sample (U937 48 h)	ctrl	AIC	Bq	AraC 10	AraC 100	AraC 1000

For Figure 2.

U937 72 h

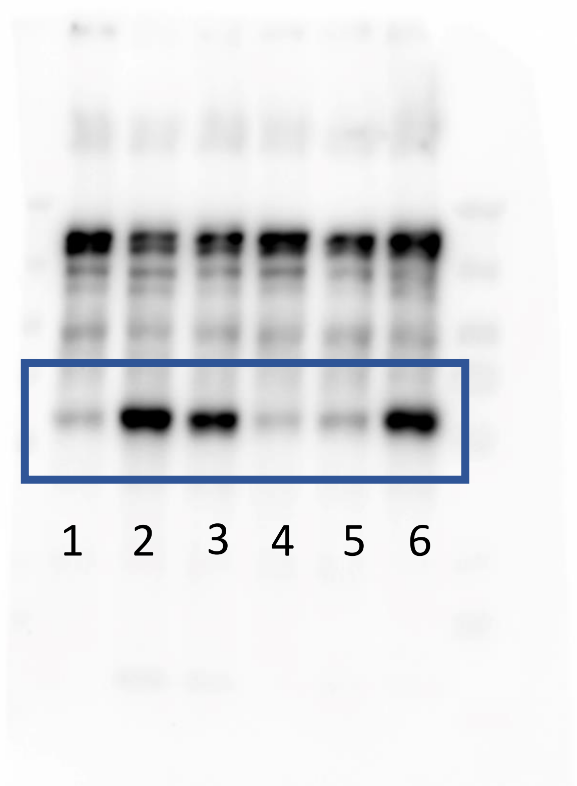

Fig.2.D WB5 p-Chk1

Lane no.	1	2	3	4	5	6
Sample (U937 72 h)	ctrl	AIC	Bq	AraC 10	AraC 100	AraC 1000

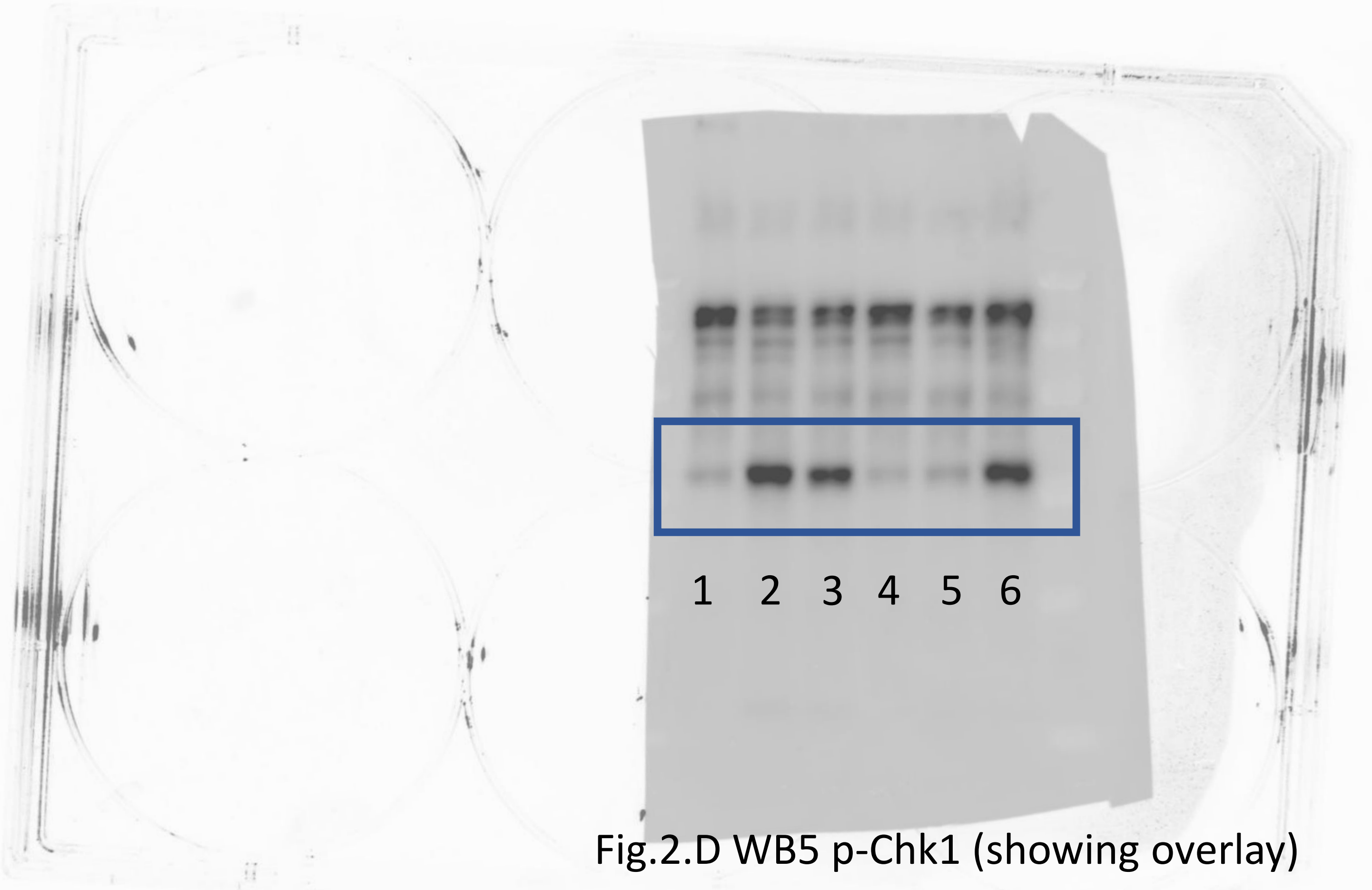

Fig.2.D WB5 p-Chk1 (showing overlay)

Lane no.	1	2	3	4	5	6
Sample (U937 72 h)	ctrl	AIC	Bq	AraC 10	AraC 100	AraC 1000

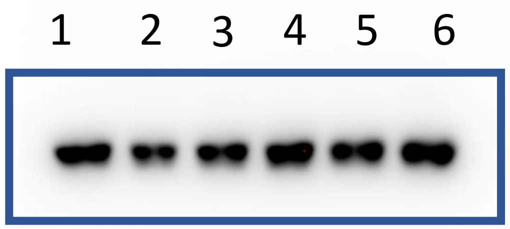

Fig.2.D WB5 beta-actin

Lane no.	1	2	3	4	5	6
Sample (U937 72 h)	ctrl	AIC	Bq	AraC 10	AraC 100	AraC 1000

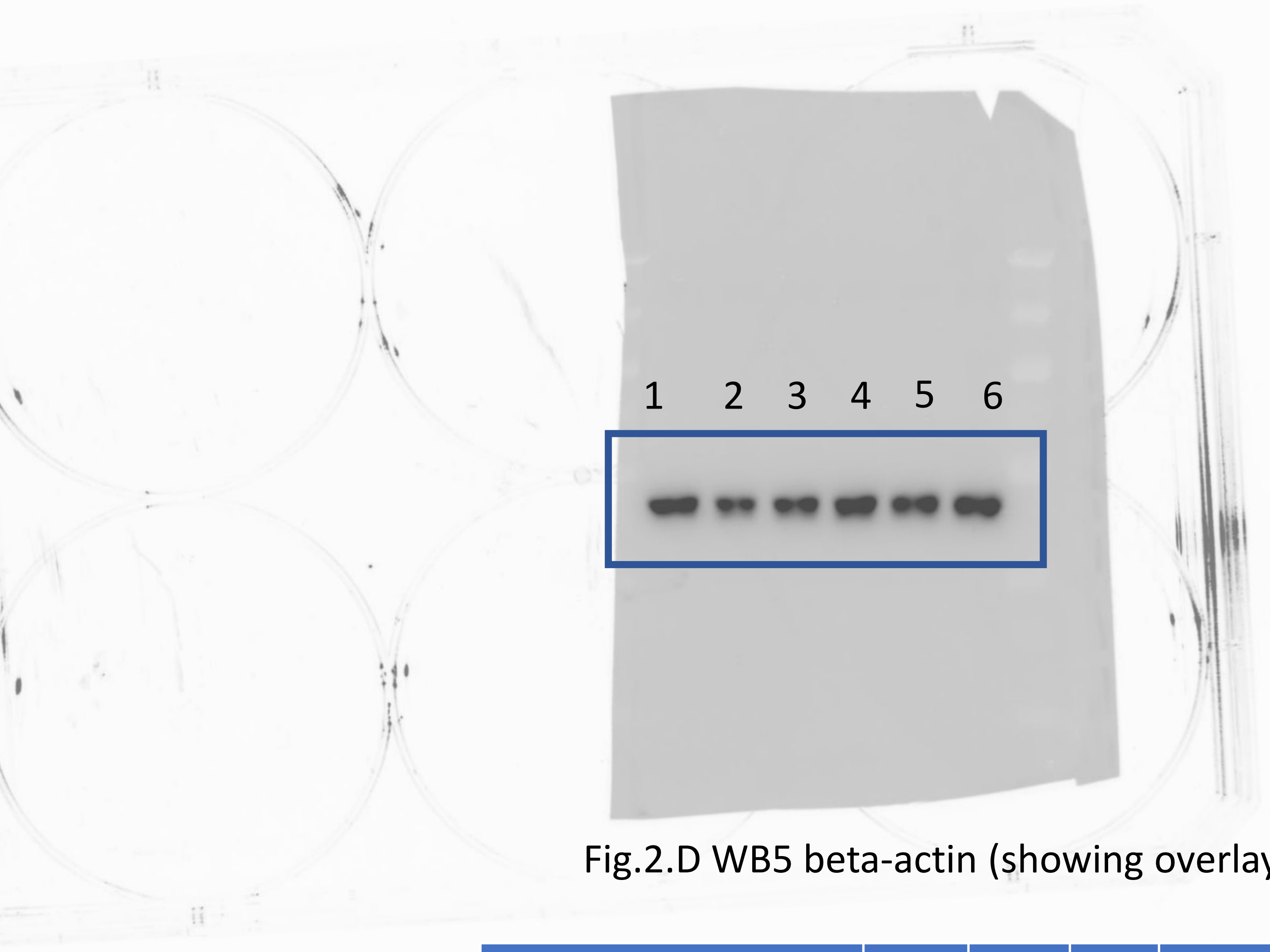

Fig.2.D WB5 beta-actin (showing overlay)

Lane no.	1	2	3	4	5	6
Sample (U937 72 h)	ctrl	AIC	Bq	AraC 10	AraC 100	AraC 1000

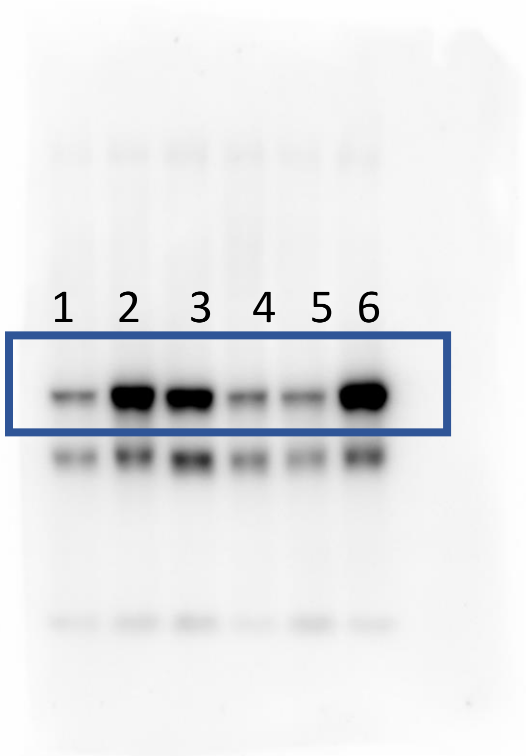

Fig.2.D WB6 Chk1

Lane no.	1	2	3	4	5	6
Sample (U937 72 h)	ctrl	AIC	Bq	AraC 10	AraC 100	AraC 1000

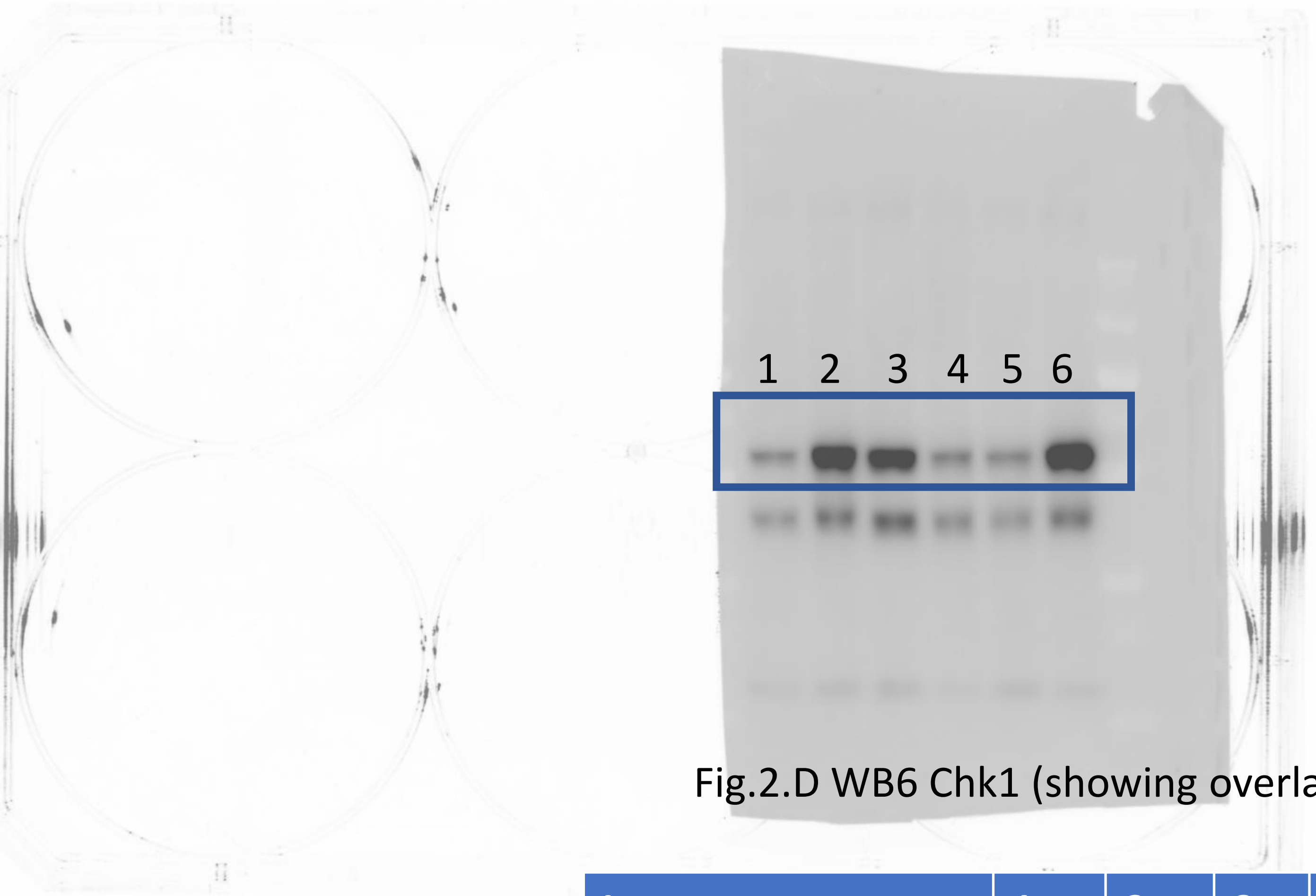

Fig.2.D WB6 Chk1 (showing overlay)

Lane no.	1	2	3	4	5	6
Sample (U937 72 h)	ctrl	AIC	Bq	AraC 10	AraC 100	AraC 1000

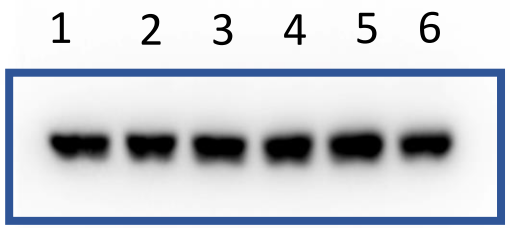

Fig.2.D WB6 beta-actin

Lane no.	1	2	3	4	5	6
Sample (U937 72 h)	ctrl	AIC	Bq	AraC 10	AraC 100	AraC 1000

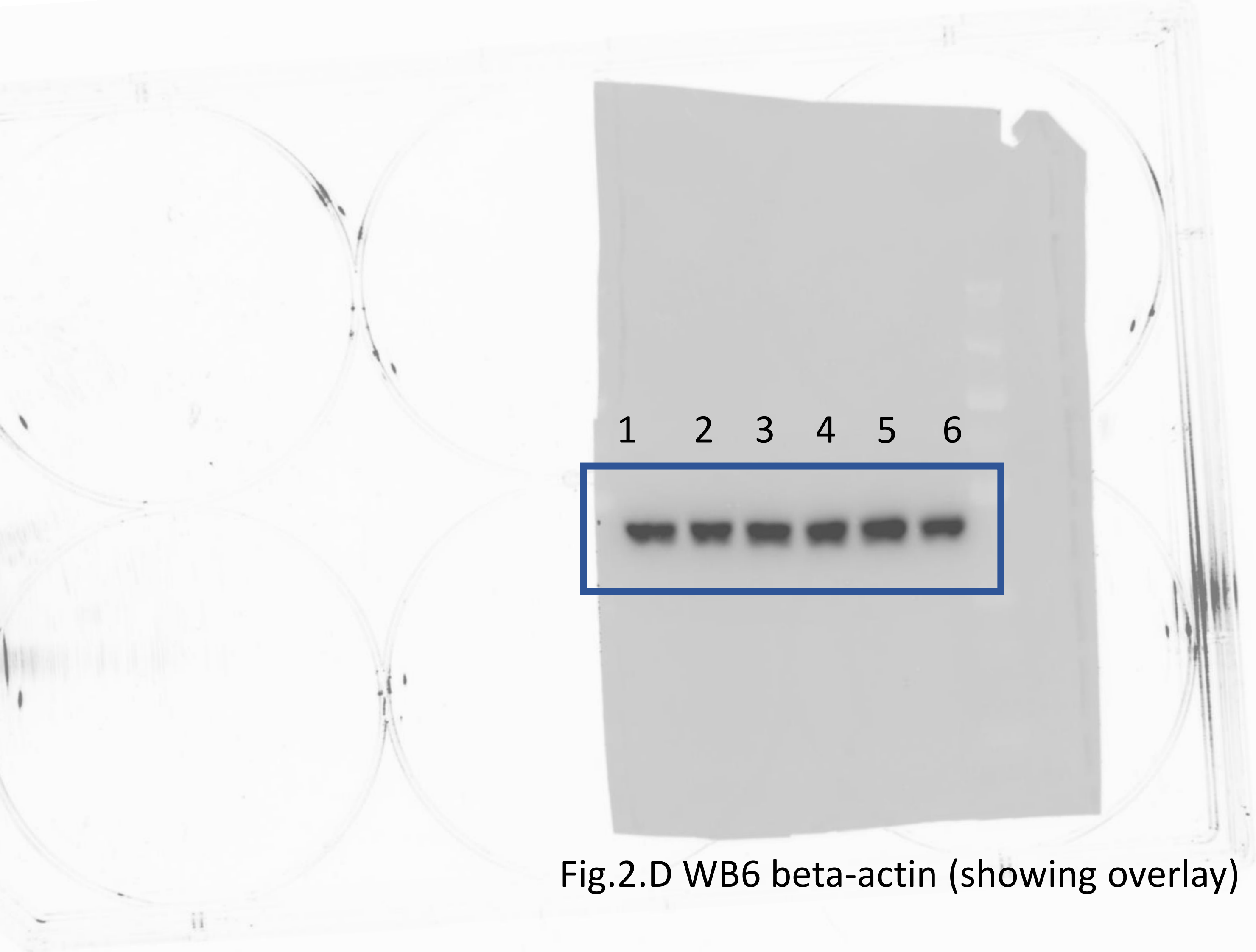

Fig.2.D WB6 beta-actin (showing overlay)

Lane no.	1	2	3	4	5	6
Sample (U937 72 h)	ctrl	AIC	Bq	AraC 10	AraC 100	AraC 1000

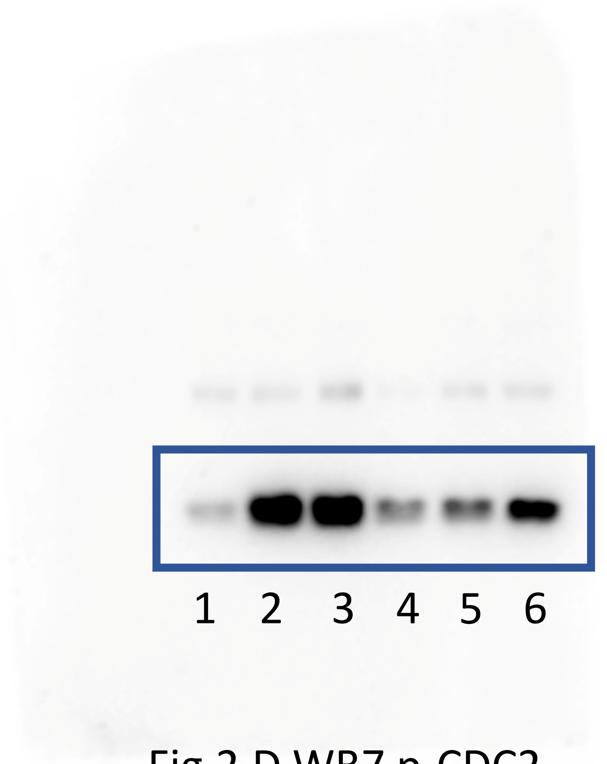

Fig.2.D WB7 p-CDC2

Lane no.	1	2	3	4	5	6
Sample (U937 72 h)	ctrl	AIC	Bq	AraC 10	AraC 100	AraC 1000

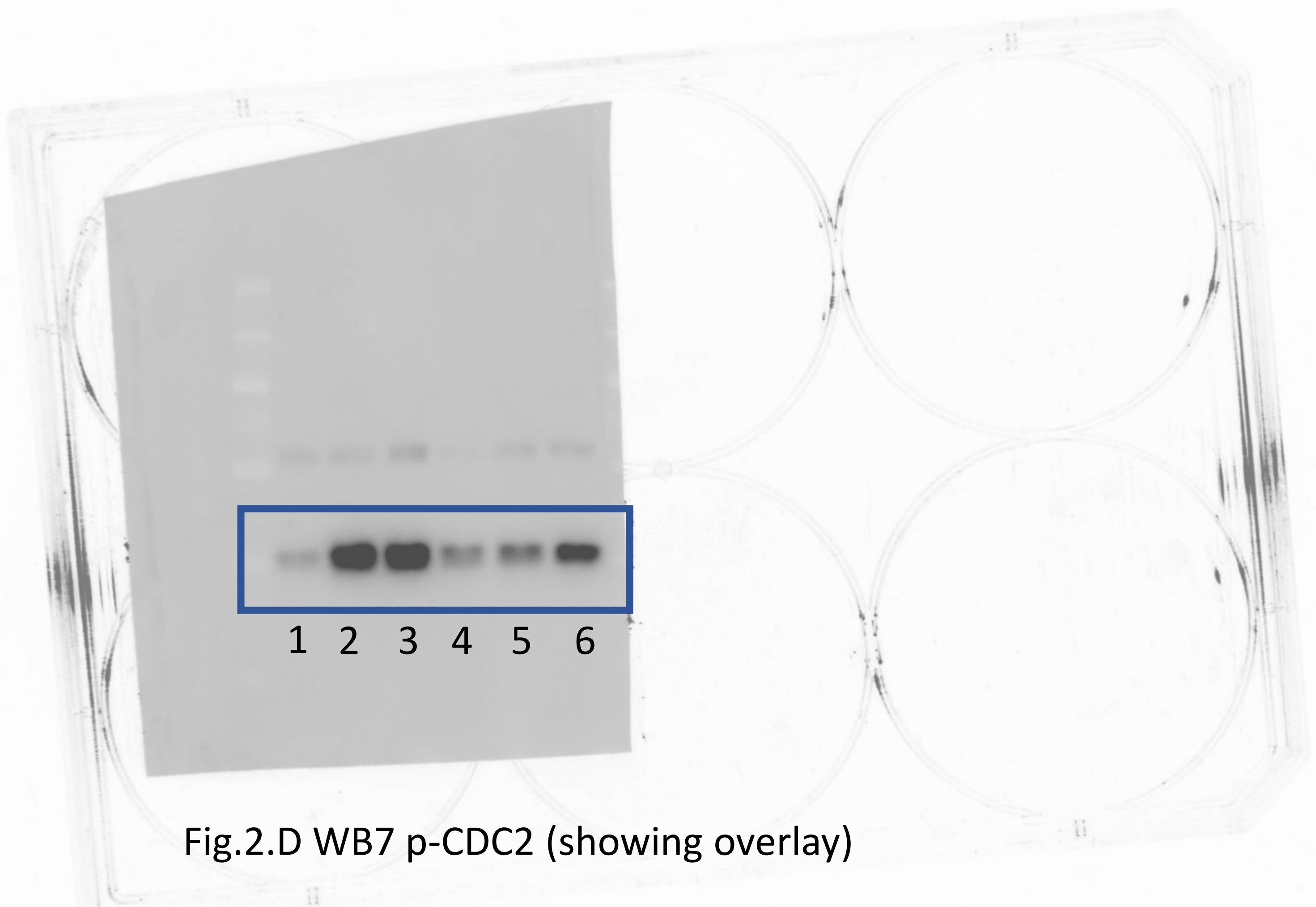

Fig.2.D WB7 p-CDC2 (showing overlay)

Lane no.	1	2	3	4	5	6
Sample (U937 72 h)	ctrl	AIC	Bq	AraC 10	AraC 100	AraC 1000

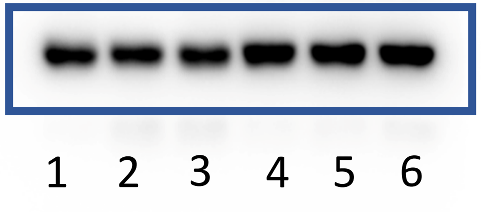

Fig.2.D WB7 beta-actin

Lane no.	1	2	3	4	5	6
Sample (U937 72 h)	ctrl	AIC	Bq	AraC 10	AraC 100	AraC 1000

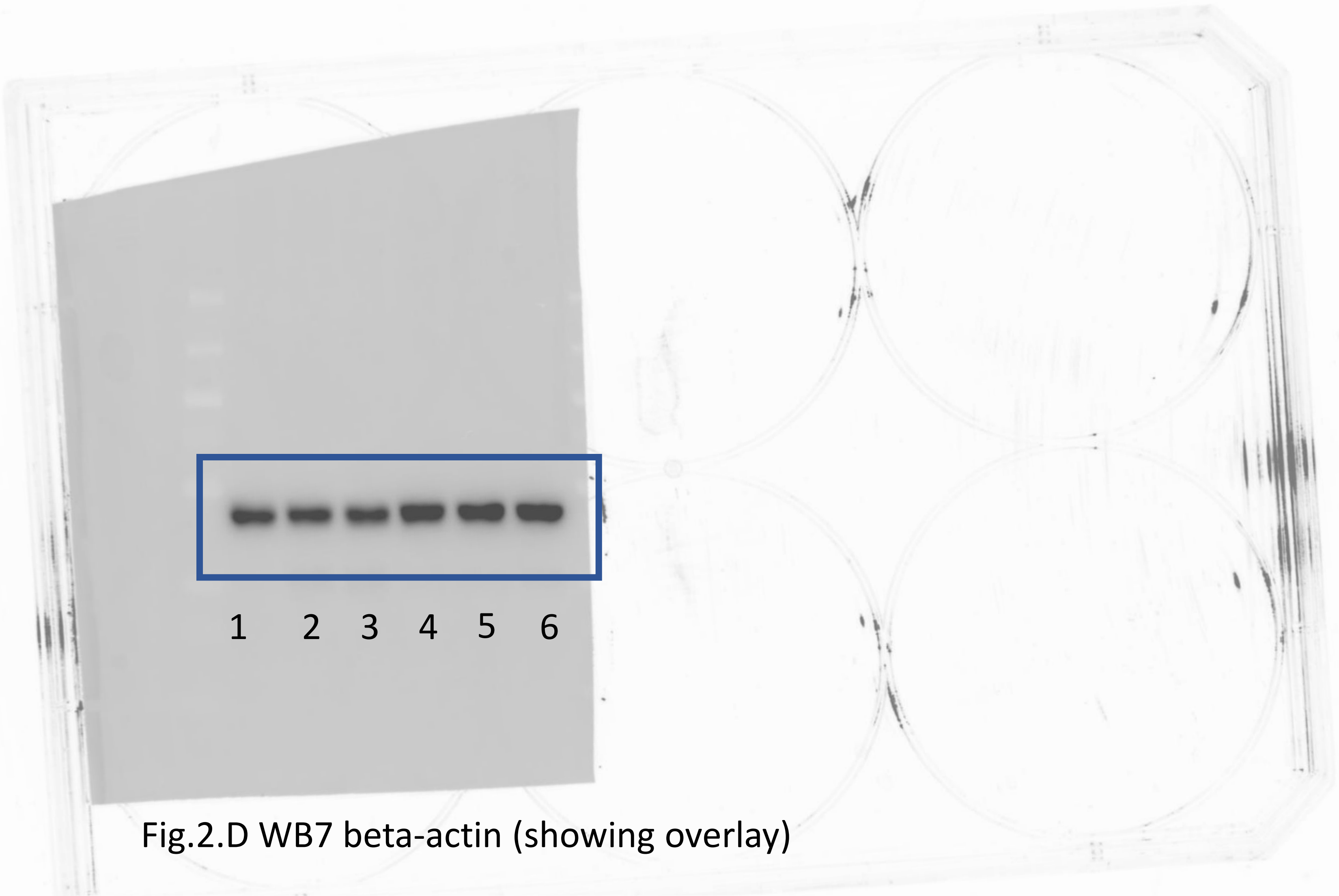

Fig.2.D WB7 beta-actin (showing overlay)

Lane no.	1	2	3	4	5	6
Sample (U937 72 h)	ctrl	AIC	Bq	AraC 10	AraC 100	AraC 1000

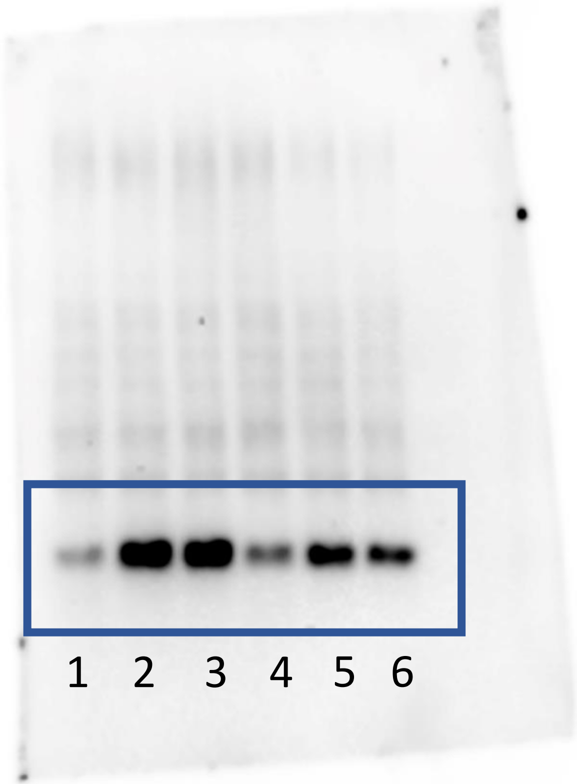

Fig.2.D WB8 CDC2

Lane no.	1	2	3	4	5	6
Sample (U937 72 h)	ctrl	AIC	Bq	AraC 10	AraC 100	AraC 1000

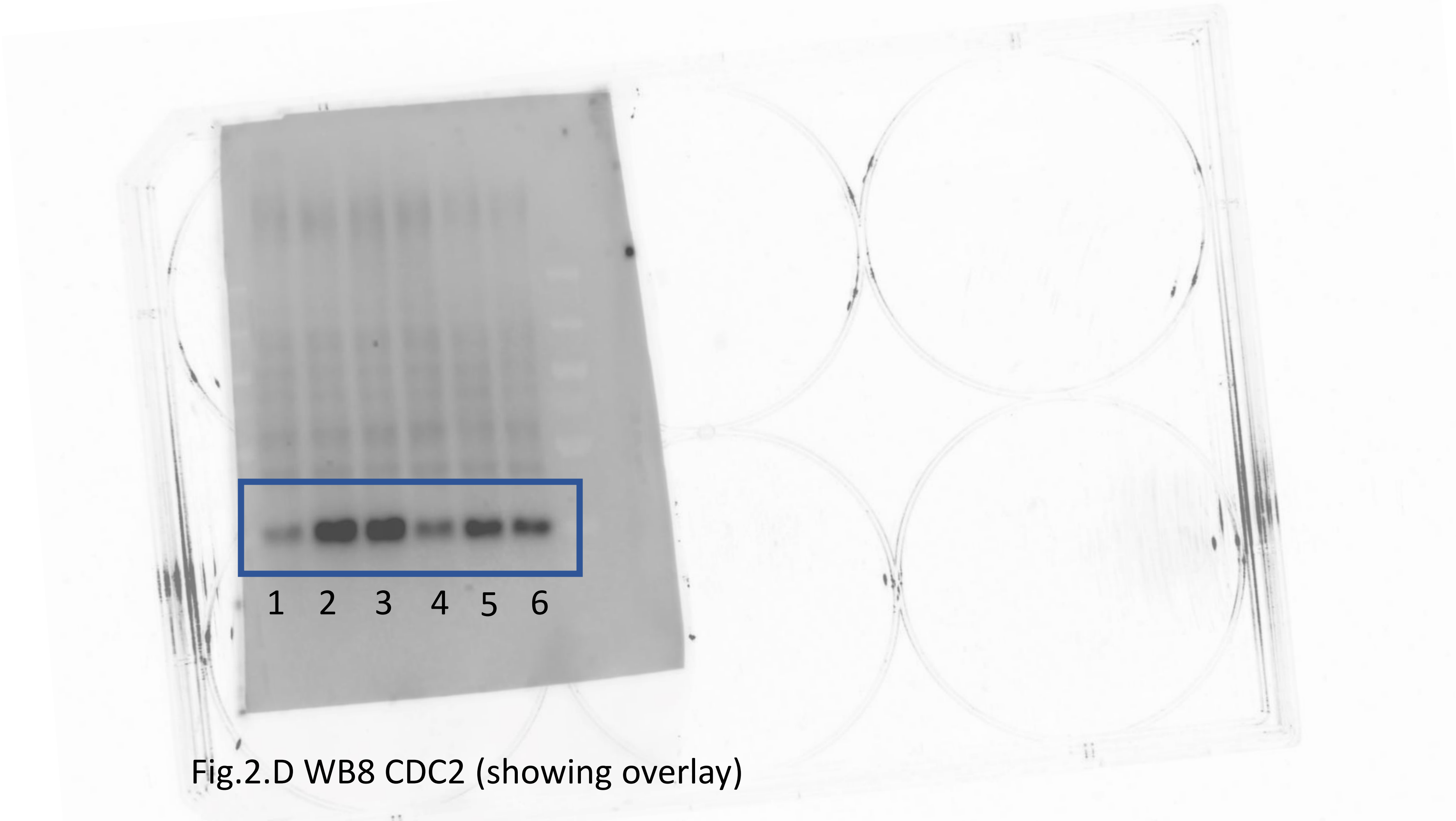

Fig.2.D WB8 CDC2 (showing overlay)

Lane no.	1	2	3	4	5	6
Sample (U937 72 h)	ctrl	AIC	Bq	AraC 10	AraC 100	AraC 1000

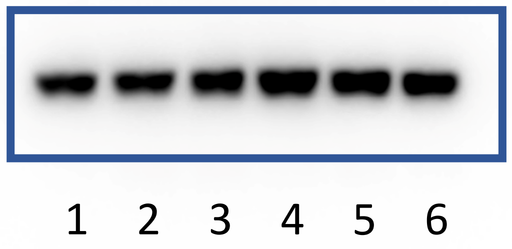

Fig.2.D WB8 beta-actin

Lane no.	1	2	3	4	5	6
Sample (U937 72 h)	ctrl	AIC	Bq	AraC 10	AraC 100	AraC 1000

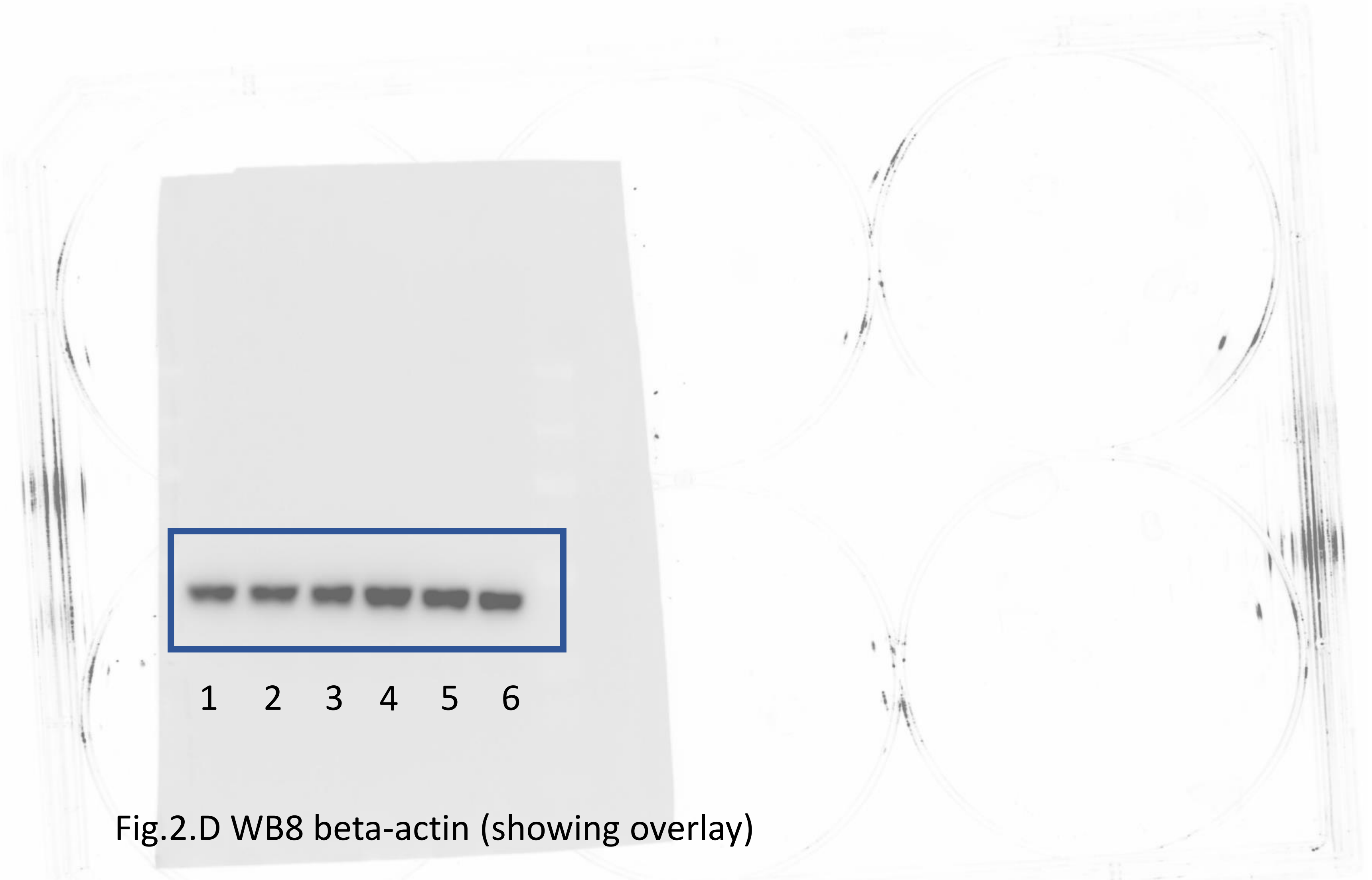

Fig.2.D WB8 beta-actin (showing overlay)

Lane no.	1	2	3	4	5	6
Sample (U937 72 h)	ctrl	AIC	Bq	AraC 10	AraC 100	AraC 1000

For Figure 3.

Torin2

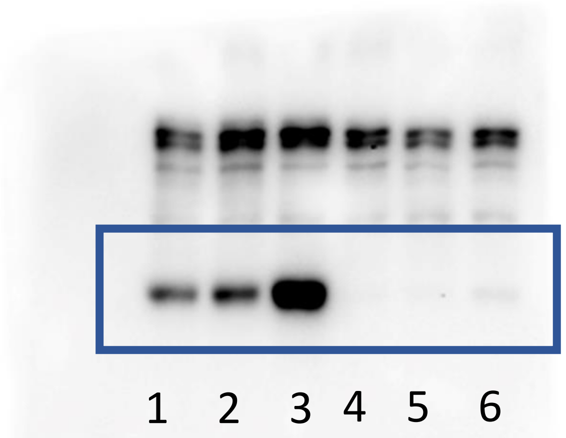

Fig.3.A WB1 p-Chk1

Lane no.	1	2	3	4	5	6
Sample	ctrl			Torin2		
	ctrl	AraC10	AraC100	ctrl	AraC10	AraC100

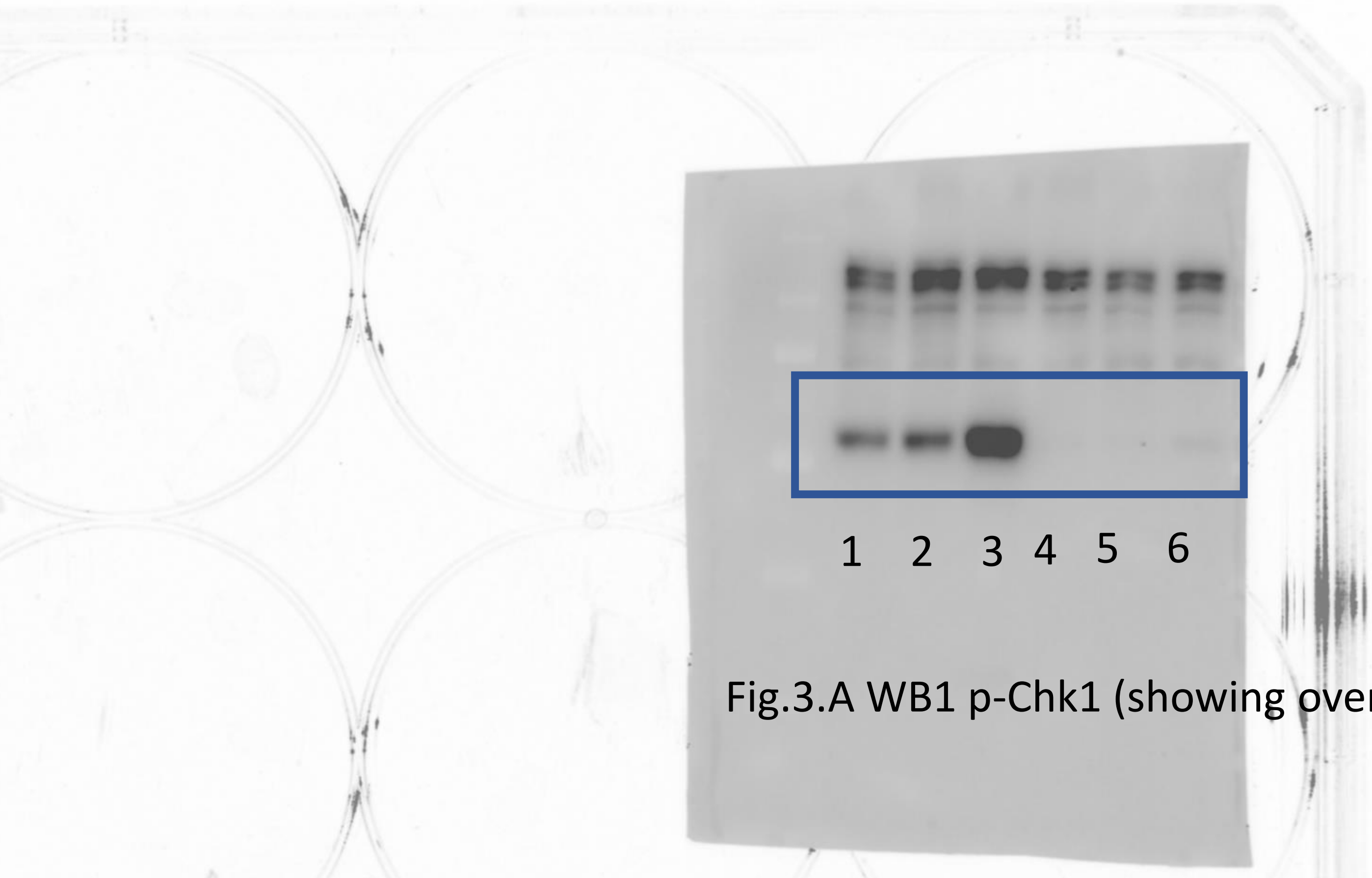

Fig.3.A WB1 p-Chk1 (showing overlay)

Lane no.	1	2	3	4	5	6
Sample	ctrl			Torin2		
	ctrl	AraC10	AraC100	ctrl	AraC10	AraC100

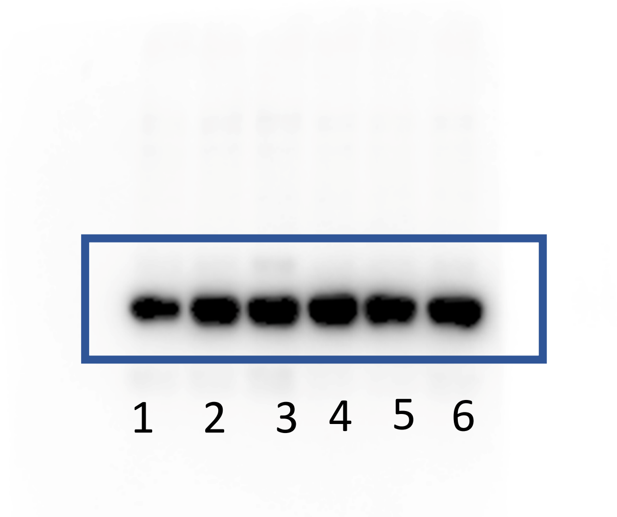

Fig.3.A WB1 beta-actin

Lane no.	1	2	3	4	5	6
Sample	ctrl			Torin2		
	ctrl	AraC10	AraC100	ctrl	AraC10	AraC100

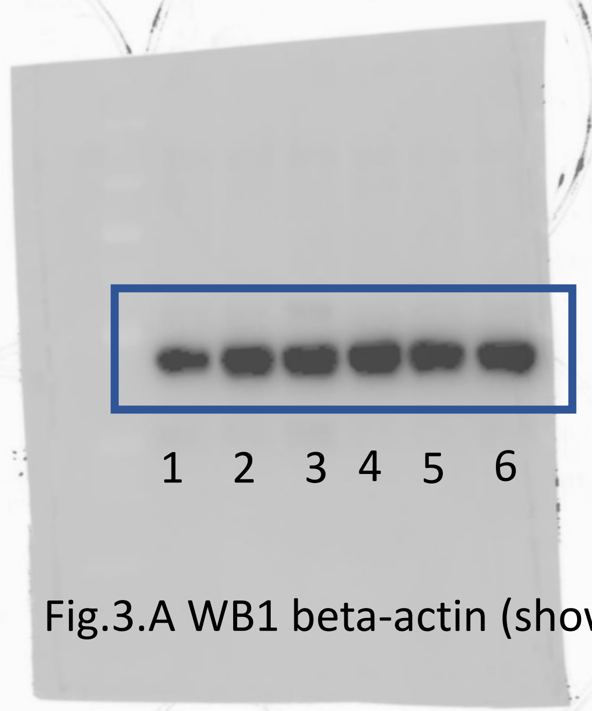

Fig.3.A WB1 beta-actin (showing overlay)

Lane no.	1	2	3	4	5	6
Sample	ctrl			Torin2		
	ctrl	AraC10	AraC100	ctrl	AraC10	AraC100

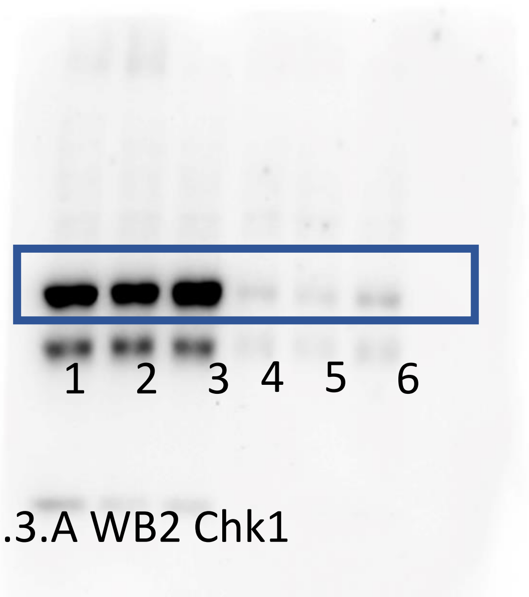

Fig.3.A WB2 Chk1

Lane no.	1	2	3	4	5	6
Sample	ctrl			Torin2		
	ctrl	AraC10	AraC100	ctrl	AraC10	AraC100

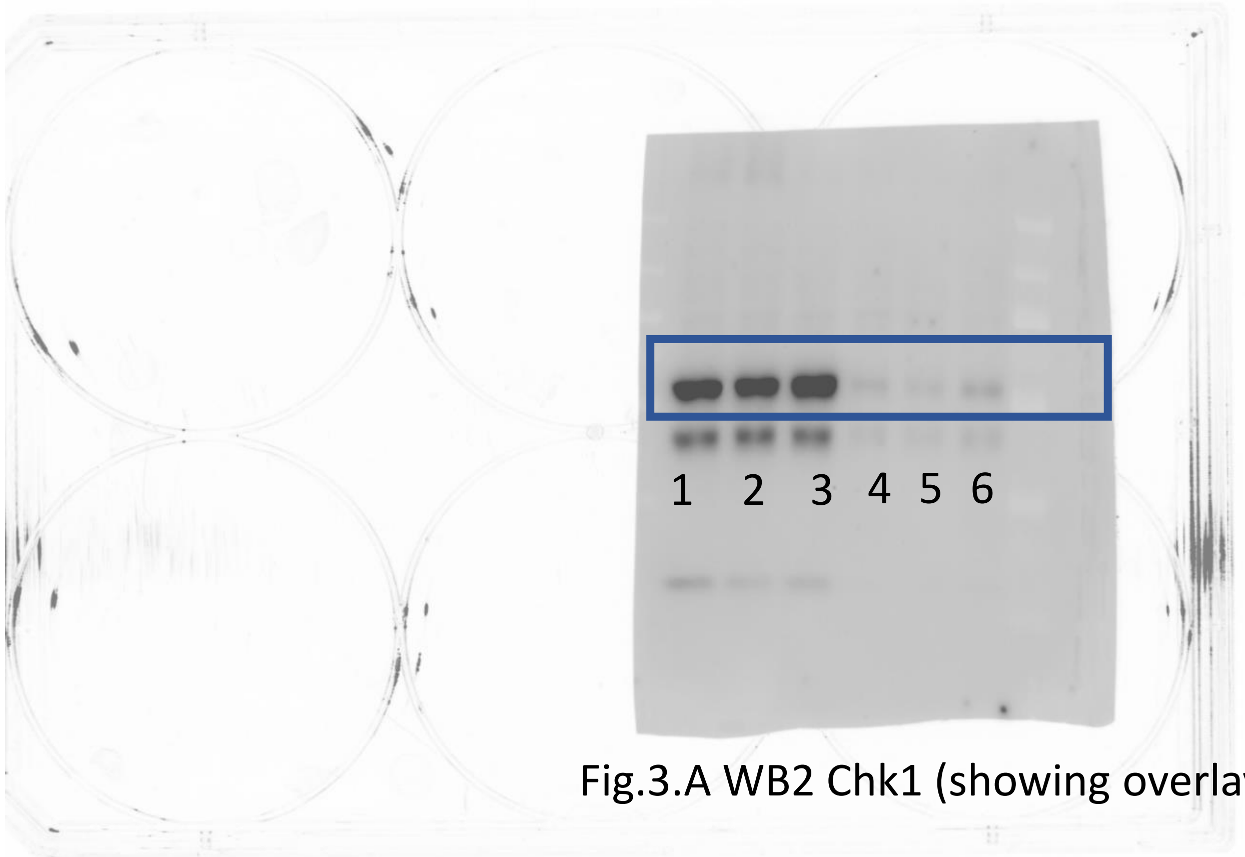

Fig.3.A WB2 Chk1 (showing overlay)

Lane no.	1	2	3	4	5	6
Sample	ctrl			Torin2		
	ctrl	AraC10	AraC100	ctrl	AraC10	AraC100

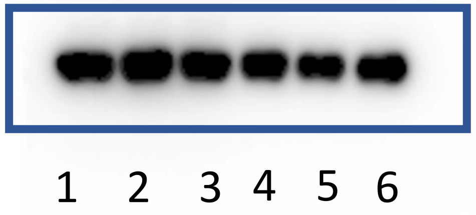

Fig.3.A WB2 beta-actin

Lane no.	1	2	3	4	5	6
Sample	ctrl			Torin2		
	ctrl	AraC10	AraC100	ctrl	AraC10	AraC100

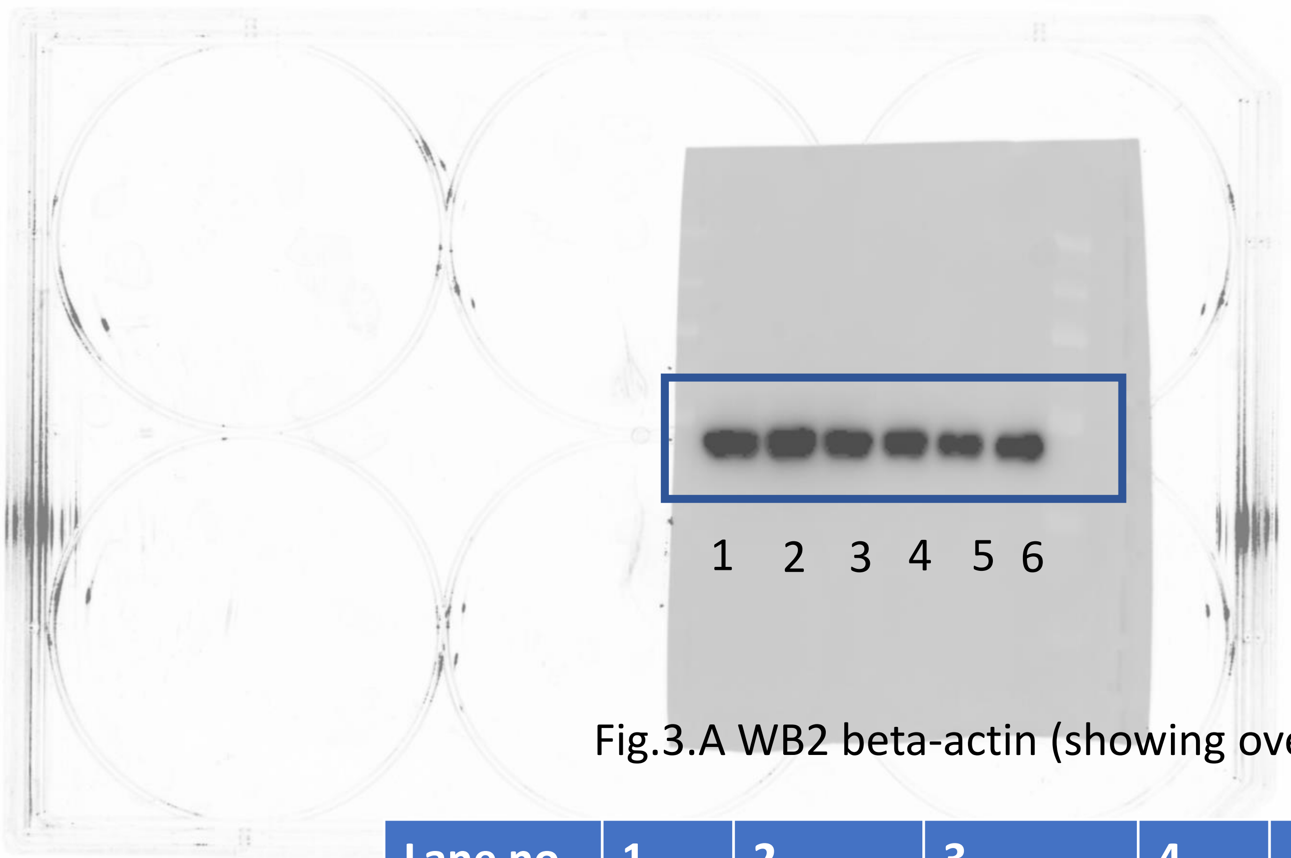

Fig.3.A WB2 beta-actin (showing overlay)

Lane no.	1	2	3	4	5	6
Sample	ctrl			Torin2		
	ctrl	AraC10	AraC100	ctrl	AraC10	AraC100

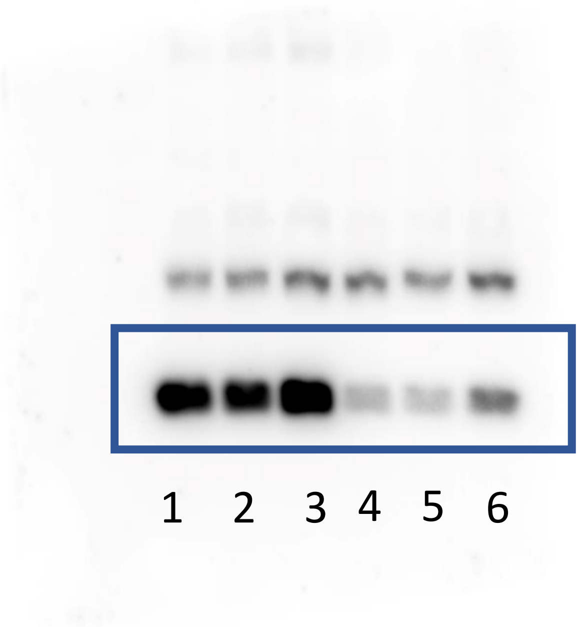

Fig.3.A WB3 p-CDC2

Lane no.	1	2	3	4	5	6
Sample	ctrl			Torin2		
	ctrl	AraC10	AraC100	ctrl	AraC10	AraC100

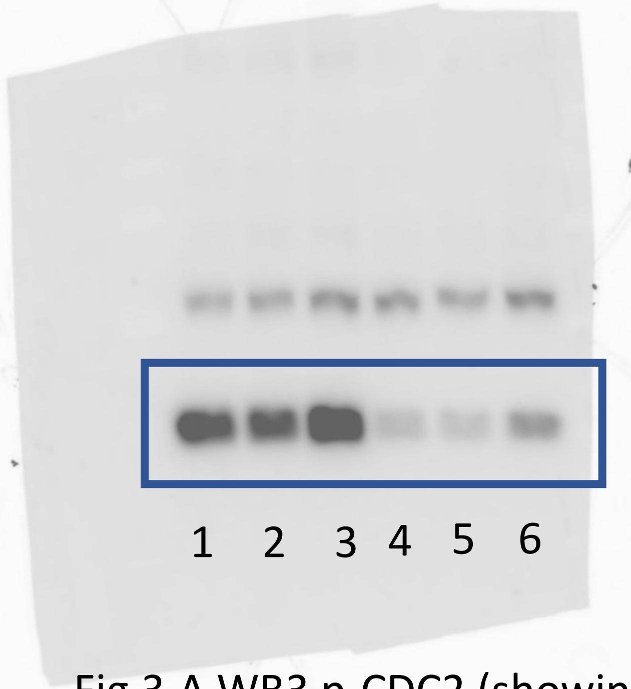

Fig.3.A WB3 p-CDC2 (showing overlay)

Lane no.	1	2	3	4	5	6
Sample	ctrl			Torin2		
	ctrl	AraC10	AraC100	ctrl	AraC10	AraC100

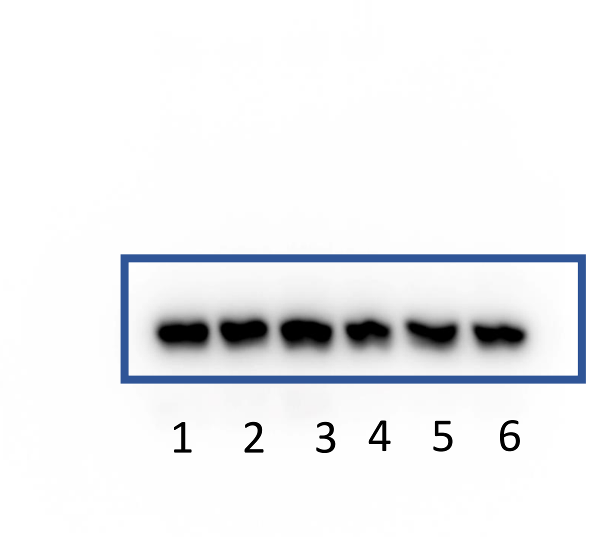

Fig.3.A WB3 beta-actin

Lane no.	1	2	3	4	5	6
Sample	ctrl			Torin2		
	ctrl	AraC10	AraC100	ctrl	AraC10	AraC100

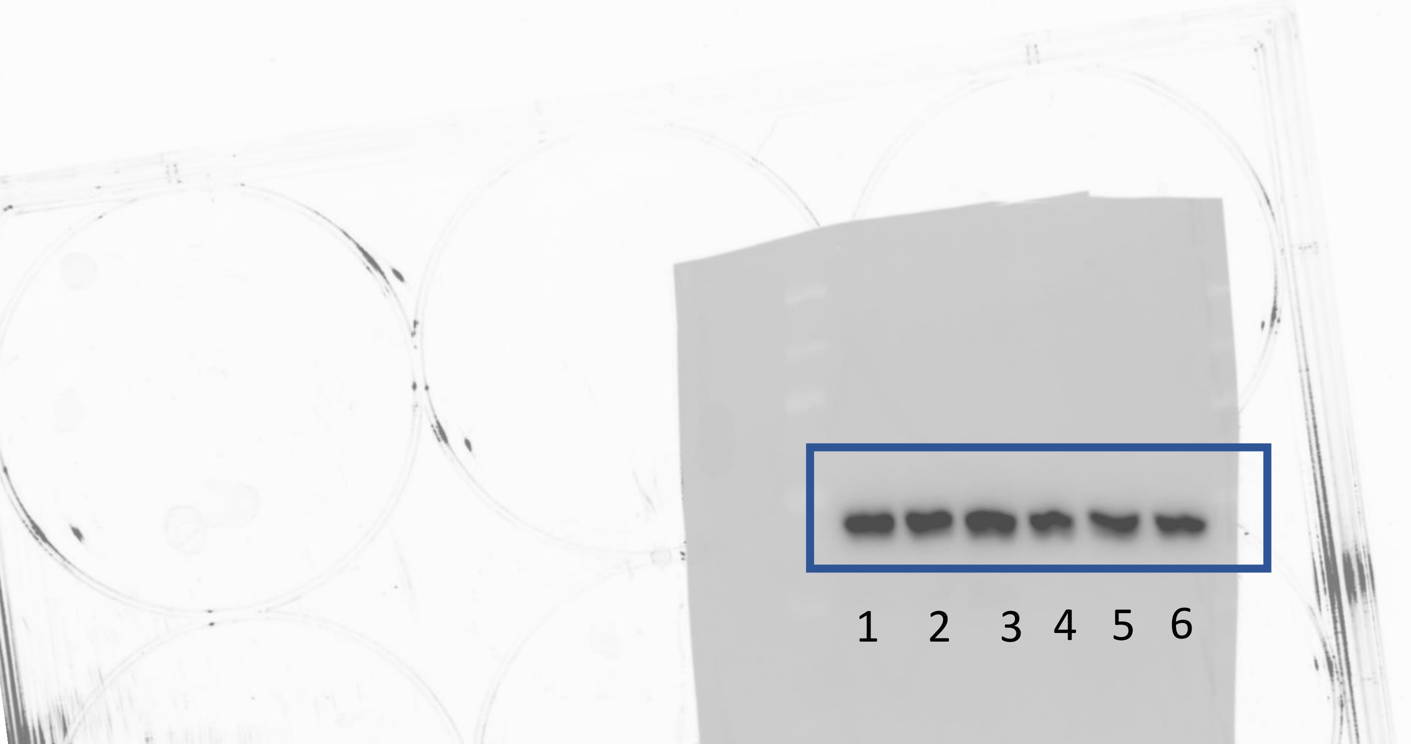

Fig.3.A WB3 beta-actin (showing overlay)

**Lane no.**	**1**	**2**	**3**	**4**	**5**	**6**
Sample	ctrl			Torin2		
	ctrl	AraC10	AraC100	ctrl	AraC10	AraC100

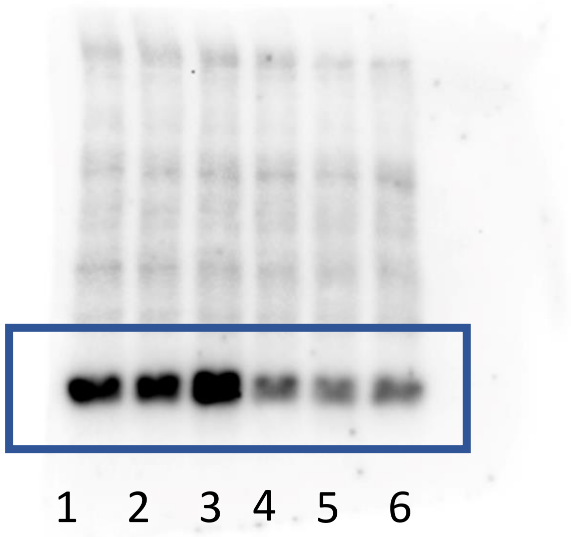

Fig.3.A WB4 CDC2

Lane no.	1	2	3	4	5	6
Sample	ctrl			Torin2		
	ctrl	AraC10	AraC100	ctrl	AraC10	AraC100

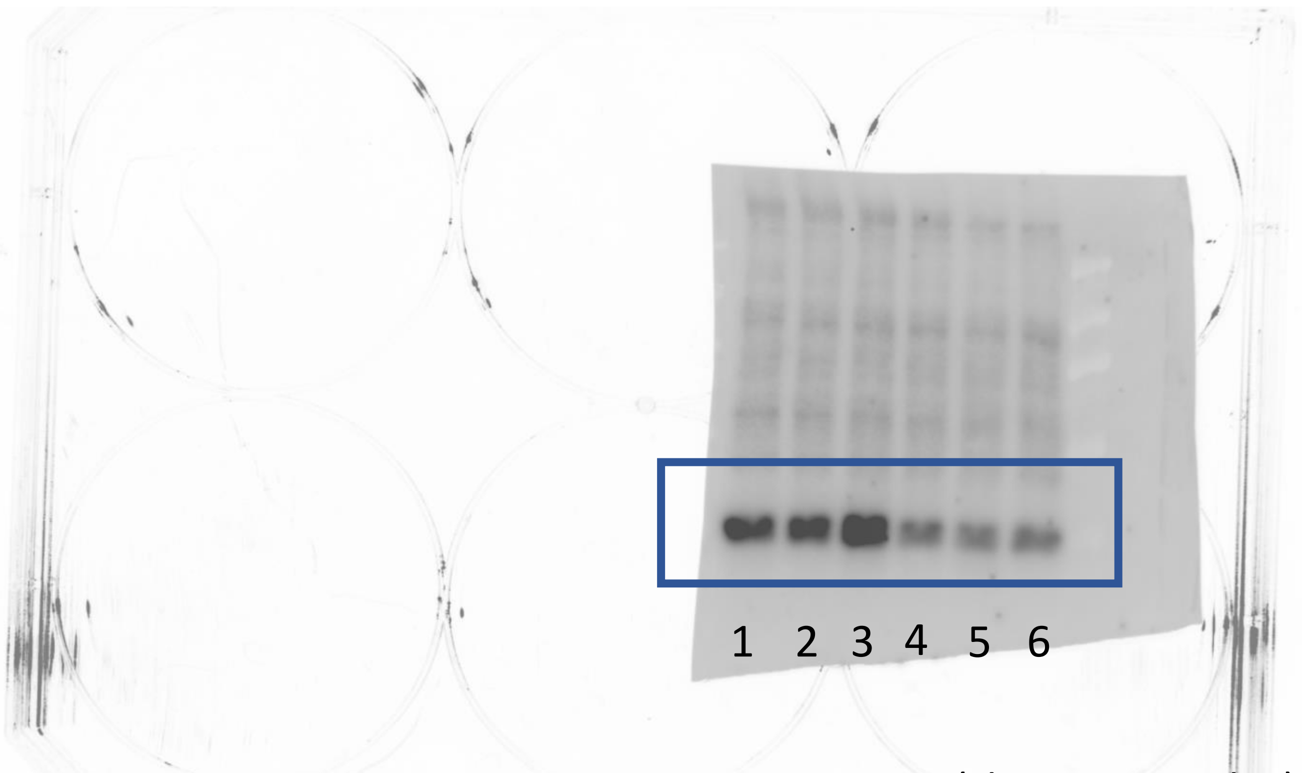

Fig.3.A WB4 CDC2 (showing overlay)

Lane no.	1	2	3	4	5	6
Sample	ctrl			Torin2		
	ctrl	AraC10	AraC100	ctrl	AraC10	AraC100

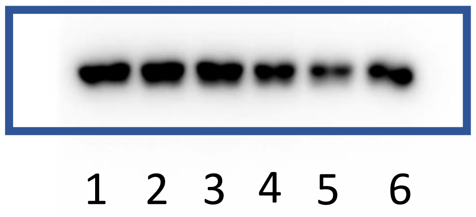

Fig.3.A WB4 beta-actin

Lane no.	1	2	3	4	5	6
Sample	ctrl			Torin2		
	ctrl	AraC10	AraC100	ctrl	AraC10	AraC100

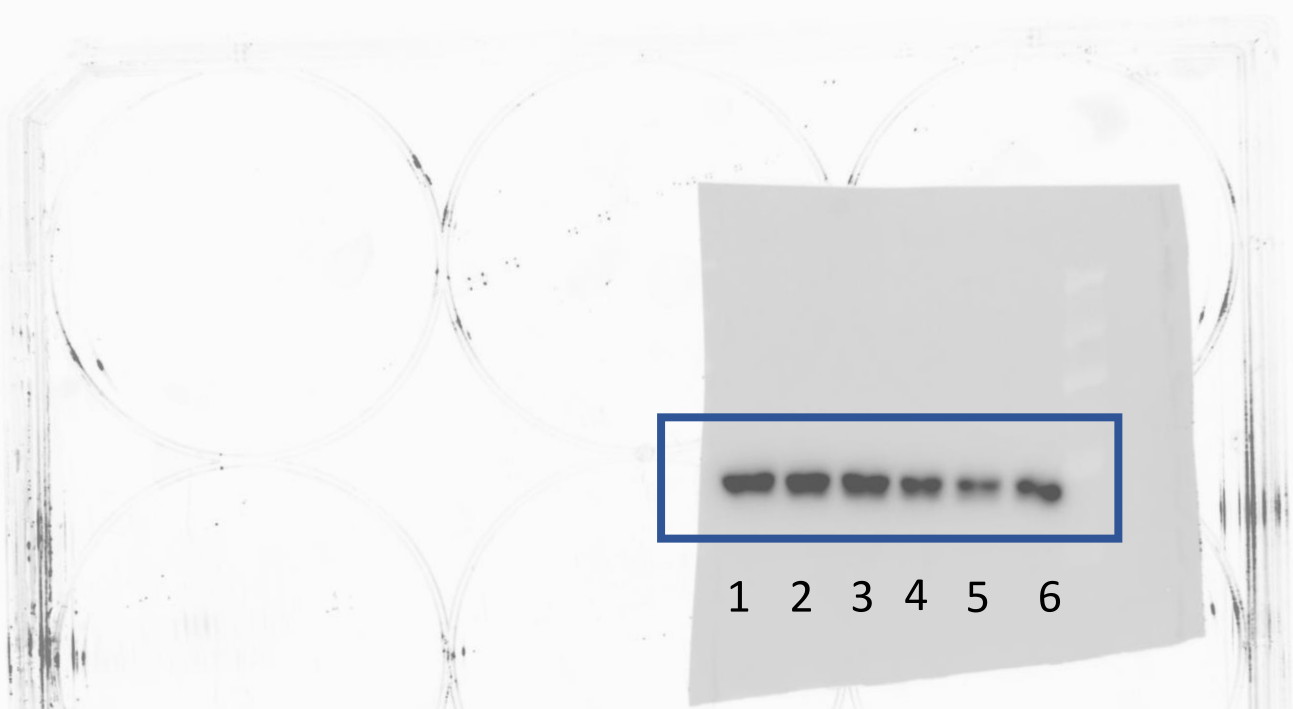

Fig.3.A WB4 beta-actin (showing overlay)

Lane no.	1	2	3	4	5	6
Sample	ctrl			Torin2		
	ctrl	AraC10	AraC100	ctrl	AraC10	AraC100

For Figure 3.

VE-821

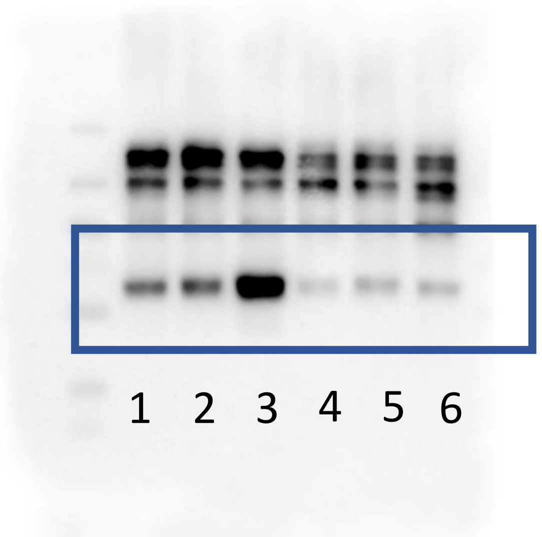

Fig.3.A WB5 p-Chk1

Lane no.	1	2	3	4	5	6
Sample	DMSO			VE-821		
	ctrl	AraC10	AraC100	ctrl	AraC10	AraC100

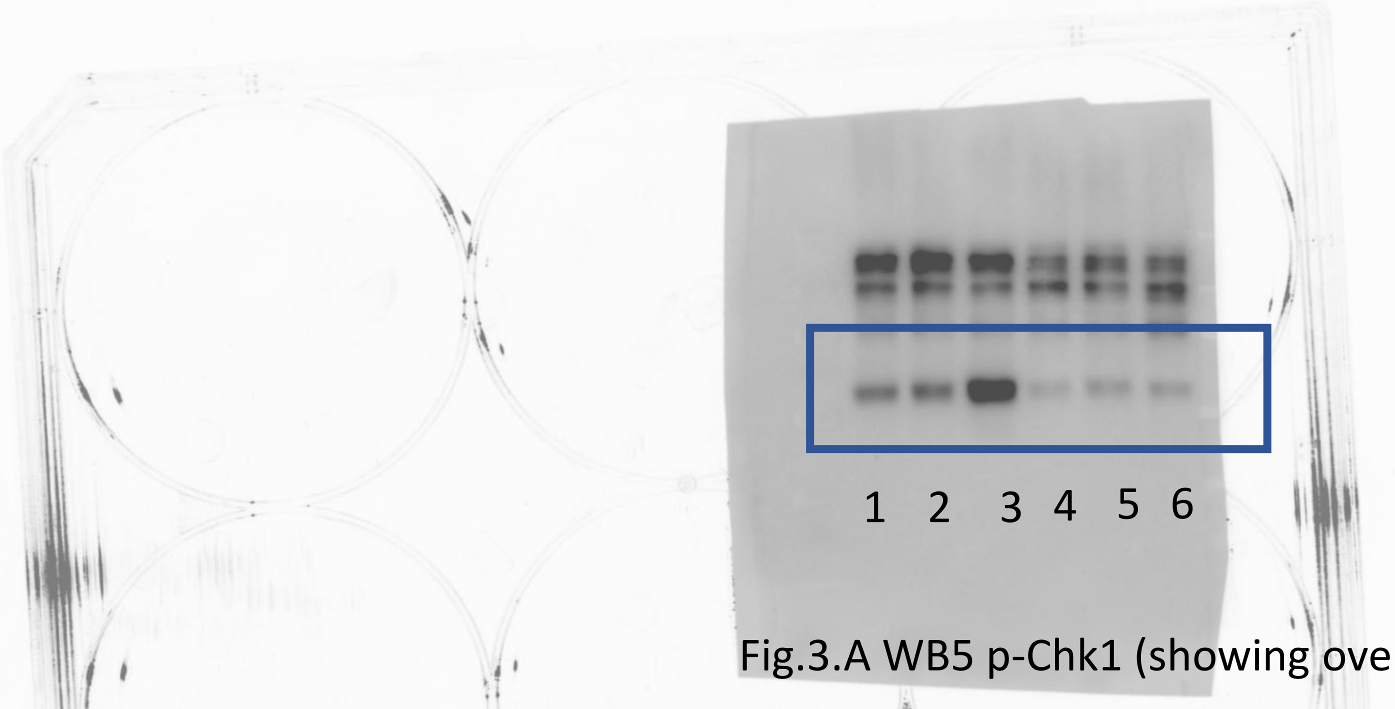

Fig.3.A WB5 p-Chk1 (showing overlay)

Lane no.	1	2	3	4	5	6
Sample	DMSO			VE-821		
	ctrl	AraC10	AraC100	ctrl	AraC10	AraC100

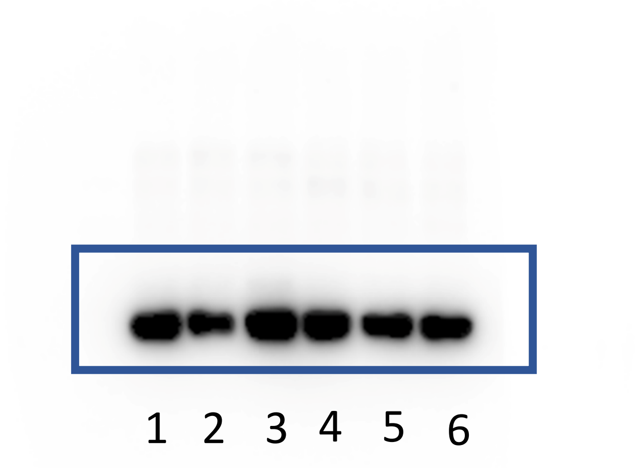

Fig.3.A WB5 beta-actin

Lane no.	1	2	3	4	5	6
Sample	DMSO			VE-821		
	ctrl	AraC10	AraC100	ctrl	AraC10	AraC100

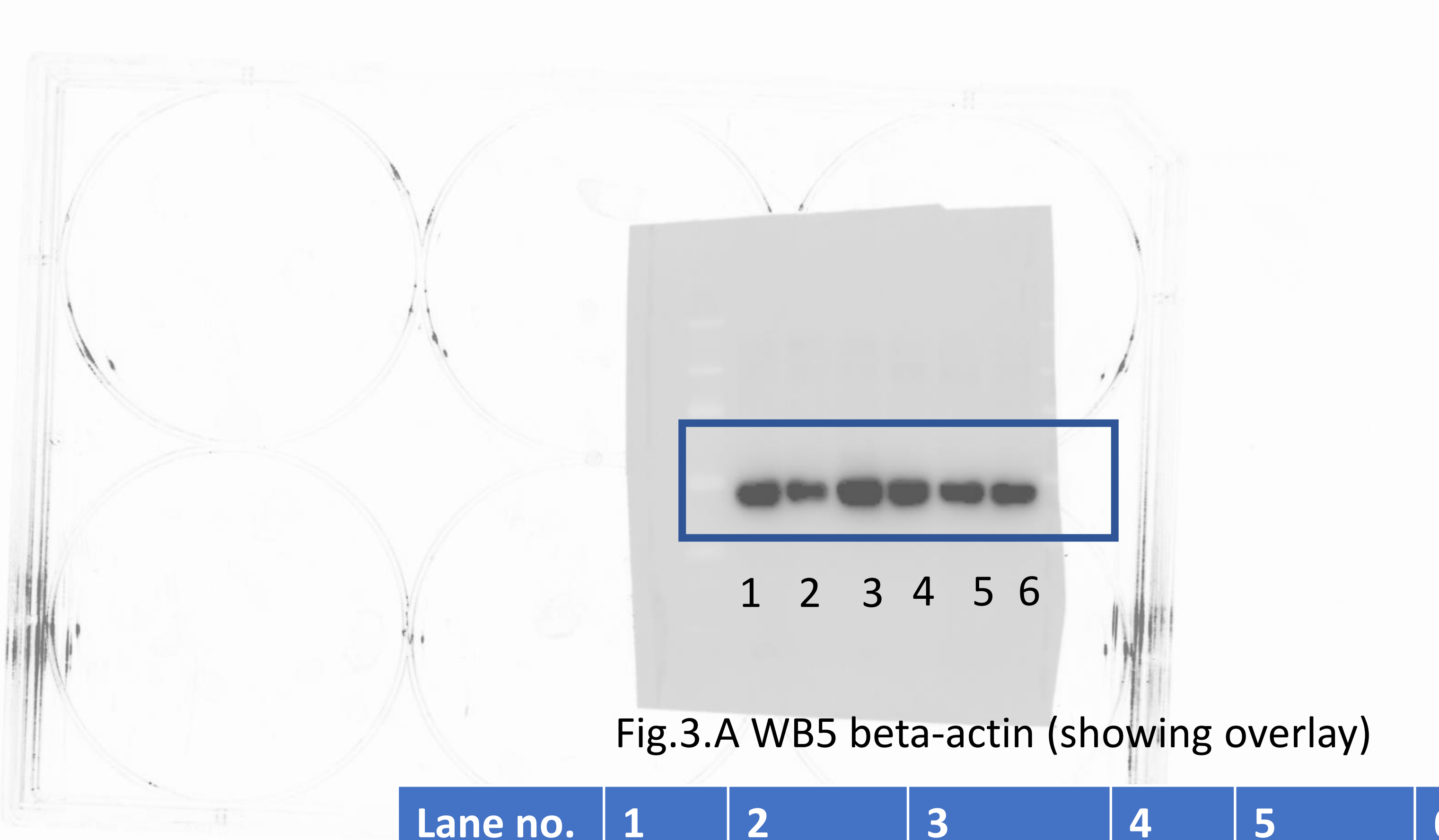

Fig.3.A WB5 beta-actin (showing overlay)

Lane no.	1	2	3	4	5	6
Sample	DMSO			VE-821		
	ctrl	AraC10	AraC100	ctrl	AraC10	AraC100

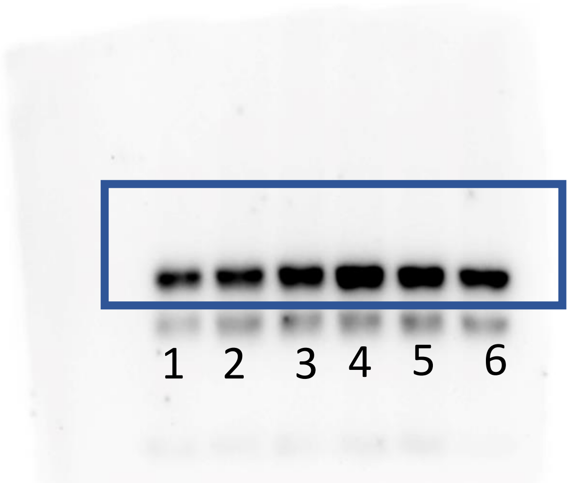

Fig.3.A WB6 Chk1

Lane no.	1	2	3	4	5	6
Sample	DMSO			VE-821		
	ctrl	AraC10	AraC100	ctrl	AraC10	AraC100

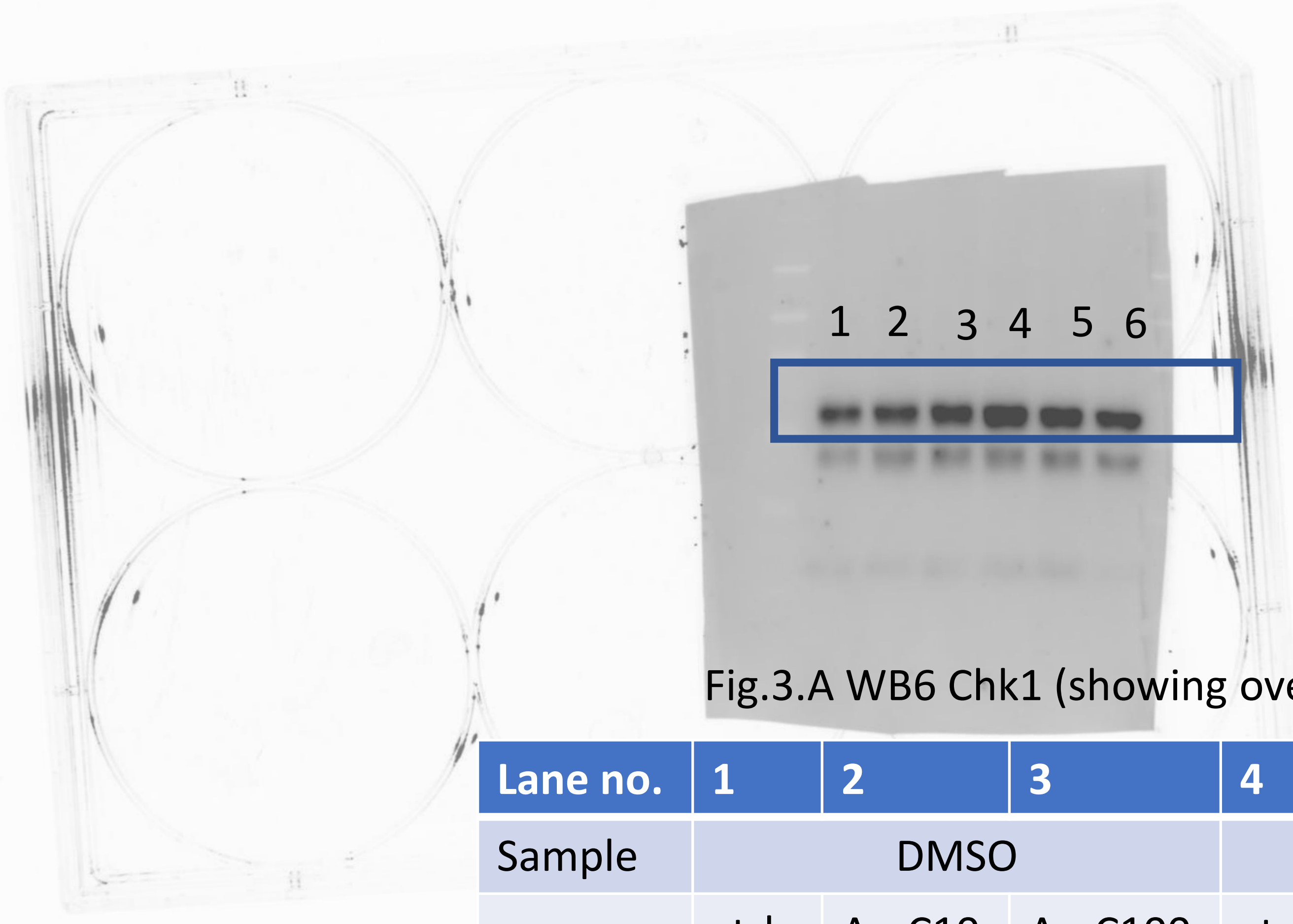

Fig.3.A WB6 Chk1 (showing overlay)

Lane no.	1	2	3	4	5	6
Sample	DMSO			VE-821		
	ctrl	AraC10	AraC100	ctrl	AraC10	AraC100

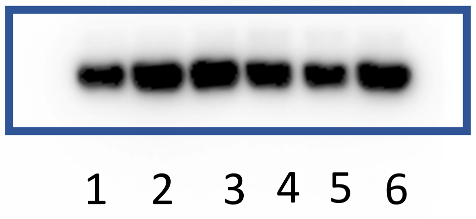

Fig.3.A WB6 beta-actin

Lane no.	1	2	3	4	5	6
Sample	DMSO			VE-821		
	ctrl	AraC10	AraC100	ctrl	AraC10	AraC100

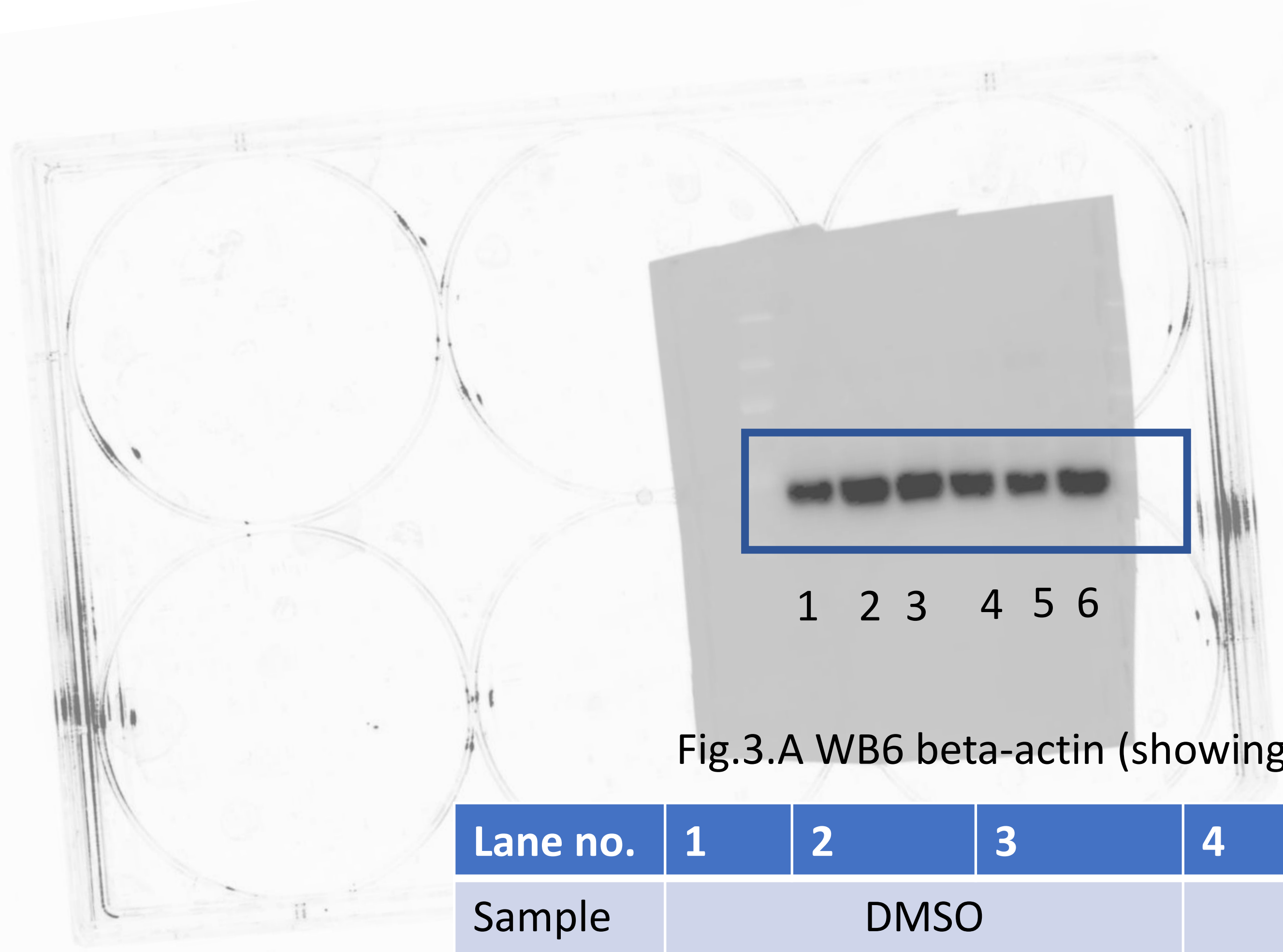

Fig.3.A WB6 beta-actin (showing overlay)

Lane no.	1	2	3	4	5	6
Sample	DMSO			VE-821		
	ctrl	AraC10	AraC100	ctrl	AraC10	AraC100

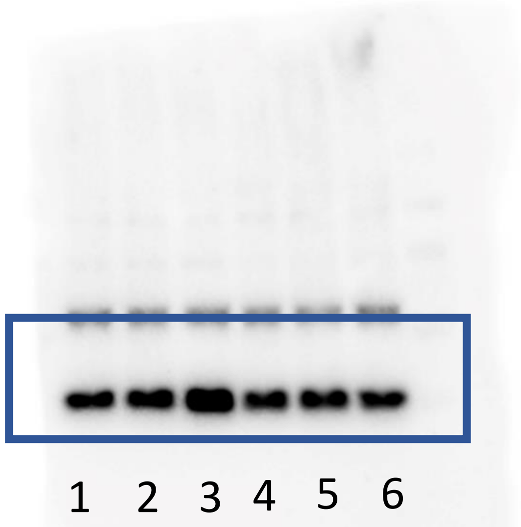

Fig.3.A WB7 p-CDC2

Lane no.	1	2	3	4	5	6
Sample	DMSO			VE-821		
	ctrl	AraC10	AraC100	ctrl	AraC10	AraC100

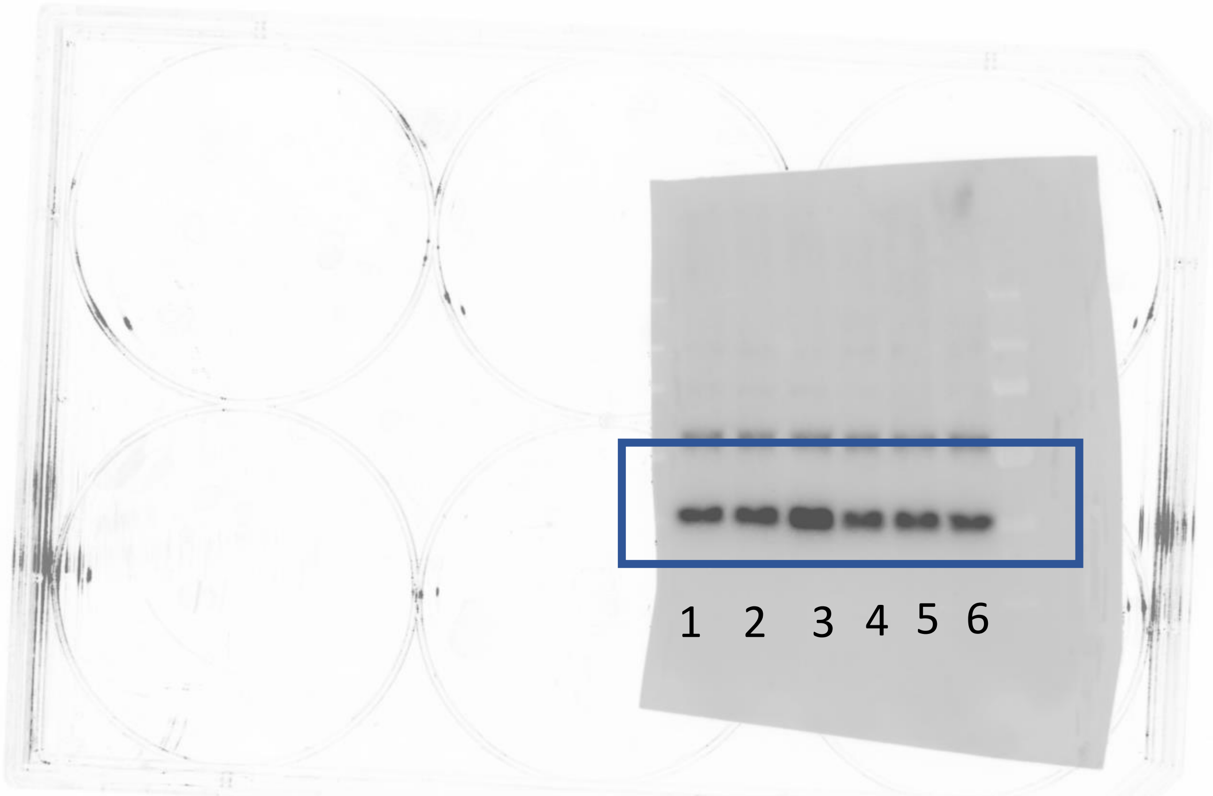

Fig.3.A WB7 p-CDC2 (showing overlay)

Lane no.	1	2	3	4	5	6
Sample	DMSO			VE-821		
	ctrl	AraC10	AraC100	ctrl	AraC10	AraC100

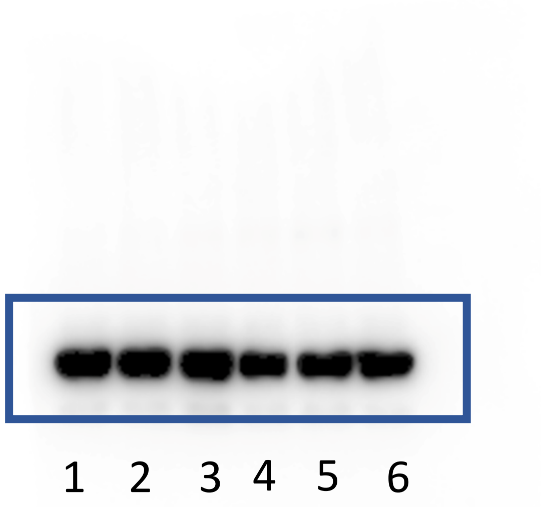

Fig.3.A WB7 beta-actin

Lane no.	1	2	3	4	5	6
Sample	DMSO			VE-821		
	ctrl	AraC10	AraC100	ctrl	AraC10	AraC100

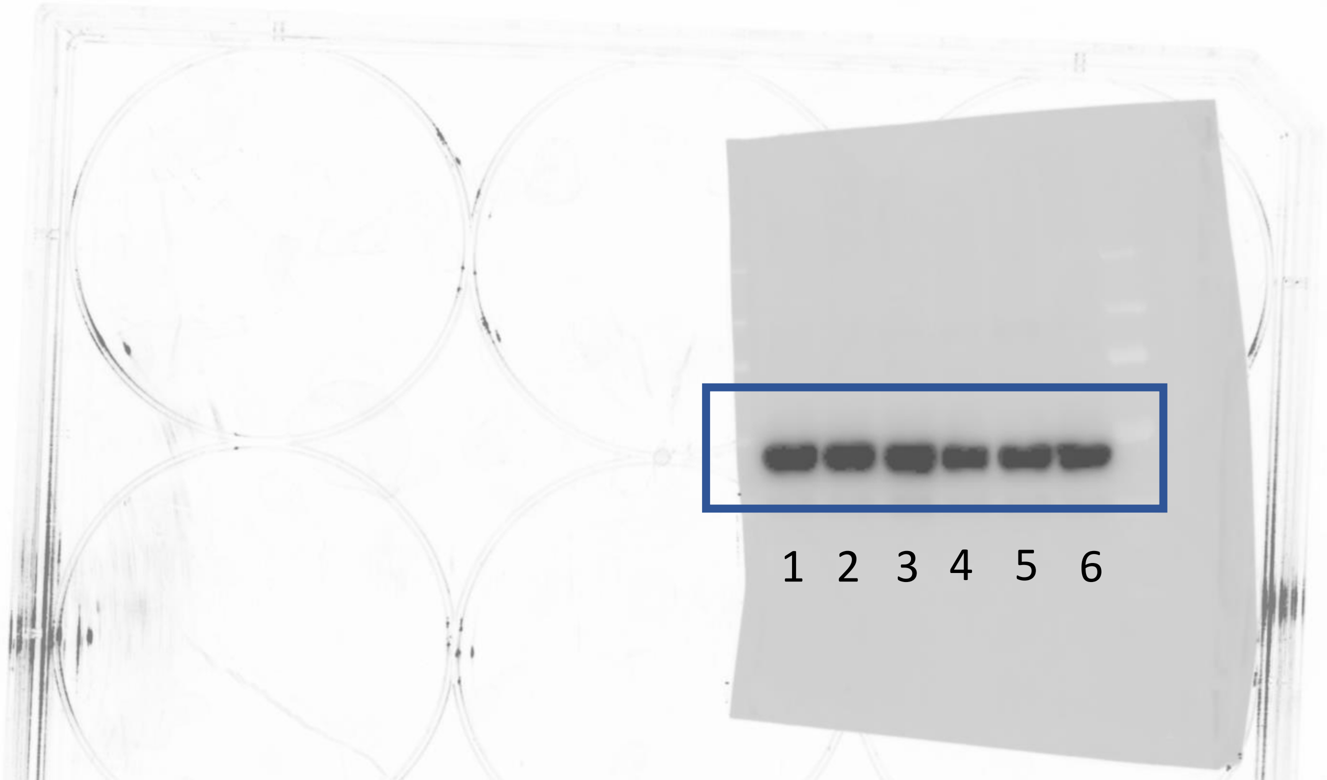

Fig.3.A WB7 beta-actin (showing overlay)

Lane no.	1	2	3	4	5	6
Sample	DMSO			VE-821		
	ctrl	AraC10	AraC100	ctrl	AraC10	AraC100

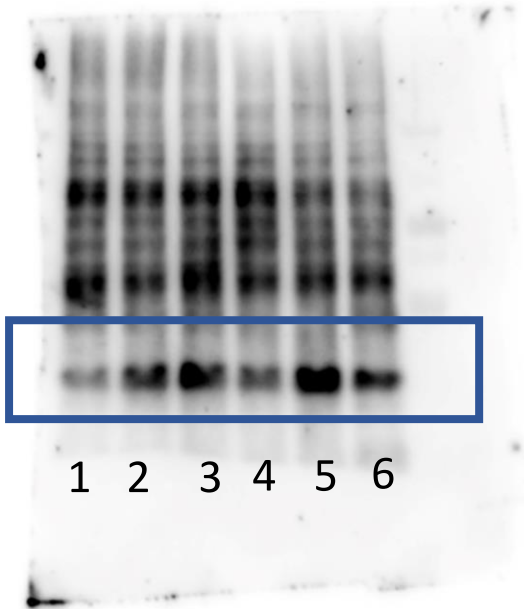

Fig.3.A WB8 CDC2

Lane no.	1	2	3	4	5	6
Sample	DMSO			VE-821		
	ctrl	AraC10	AraC100	ctrl	AraC10	AraC100

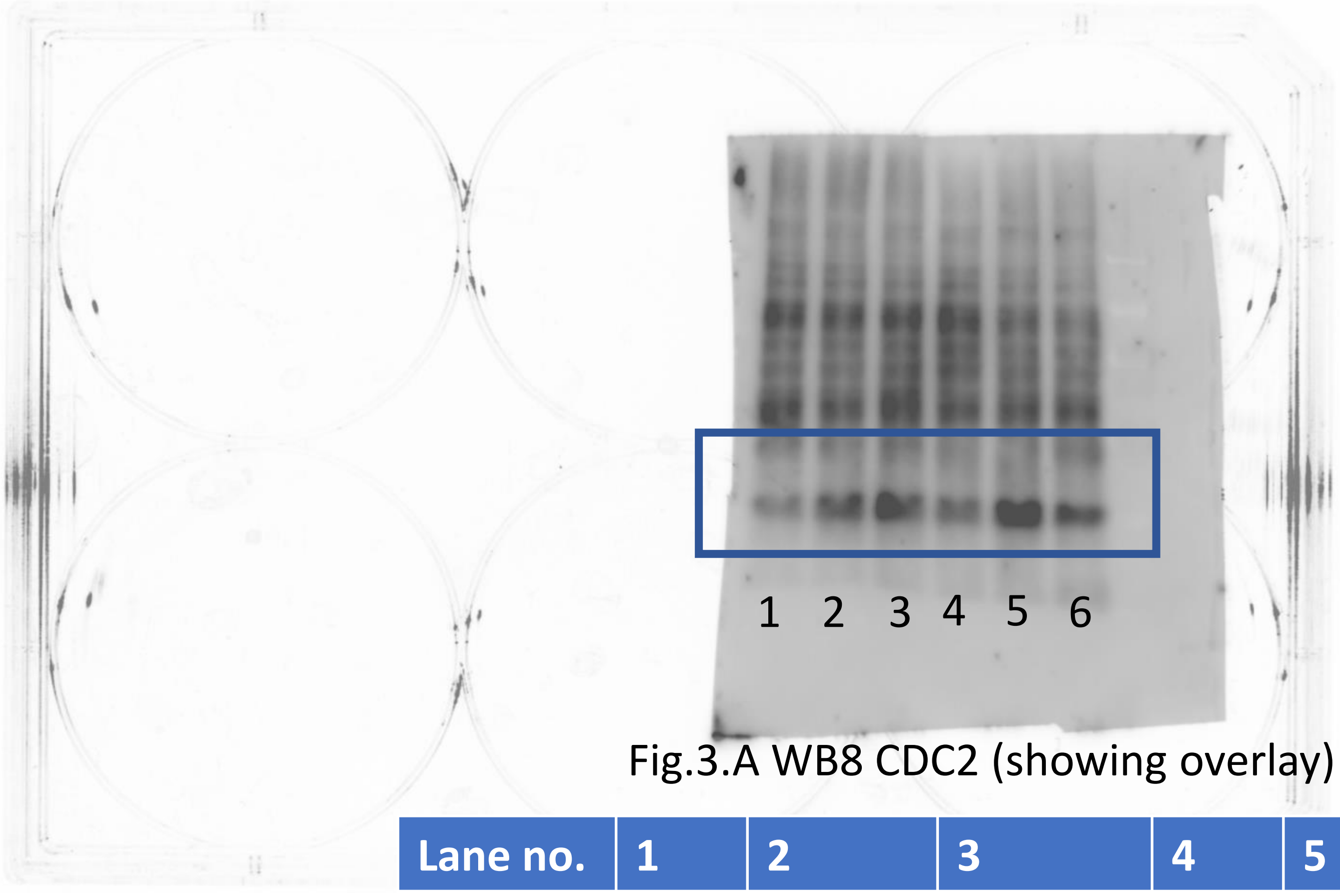

Fig.3.A WB8 CDC2 (showing overlay)

Lane no.	1	2	3	4	5	6
Sample	DMSO			VE-821		
	ctrl	AraC10	AraC100	ctrl	AraC10	AraC100

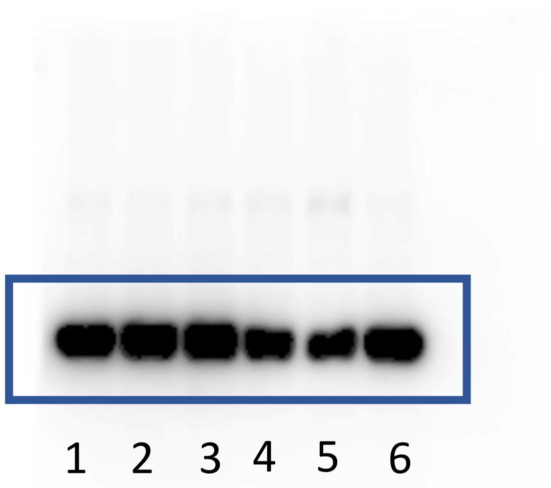

Fig.3.A WB8 beta-actin

Lane no.	1	2	3	4	5	6
Sample	DMSO			VE-821		
	ctrl	AraC10	AraC100	ctrl	AraC10	AraC100

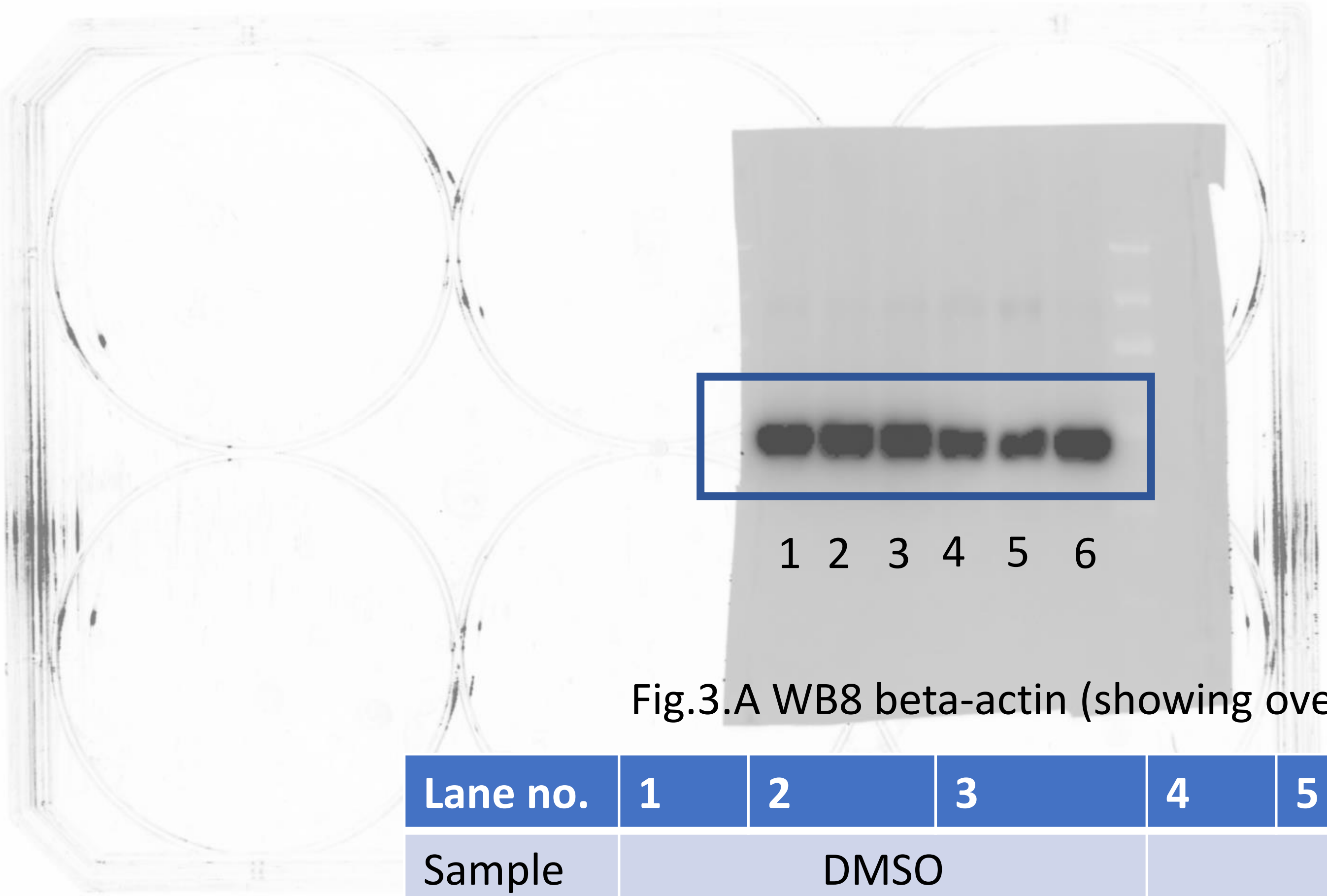

Fig.3.A WB8 beta-actin (showing overlay)

Lane no.	1	2	3	4	5	6
Sample	DMSO			VE-821		
	ctrl	AraC10	AraC100	ctrl	AraC10	AraC100

For Figure 4.

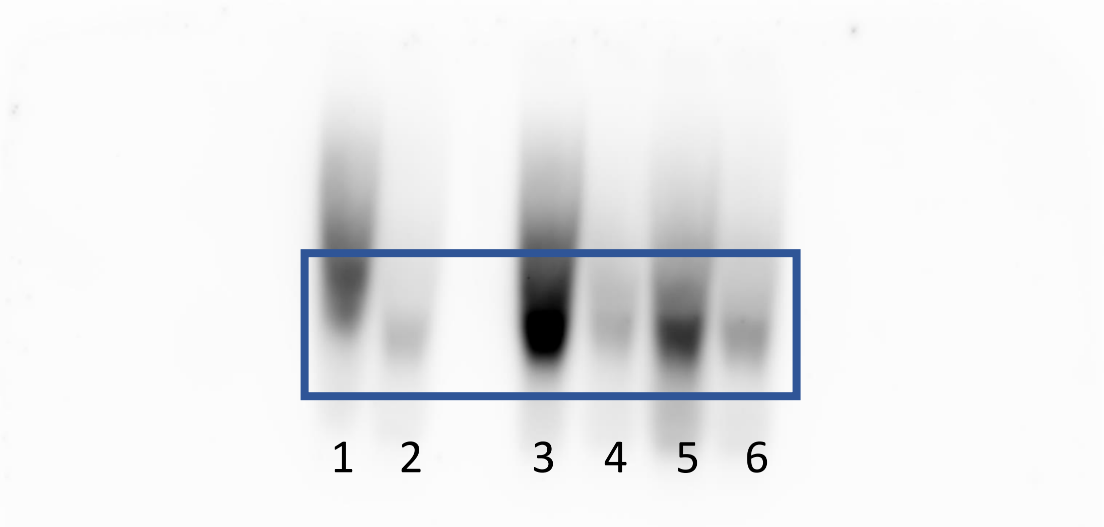

Fig.4.A EXP1 Chk1 (imaging mode: single)

Lane no.	1	2	3	4	5	6
**Sample**	U937 (3 h)		U937 (48 h)			
	ctrl		ctrl		AraC 100	
siRNA Chk1	-	+	-	+	-	+

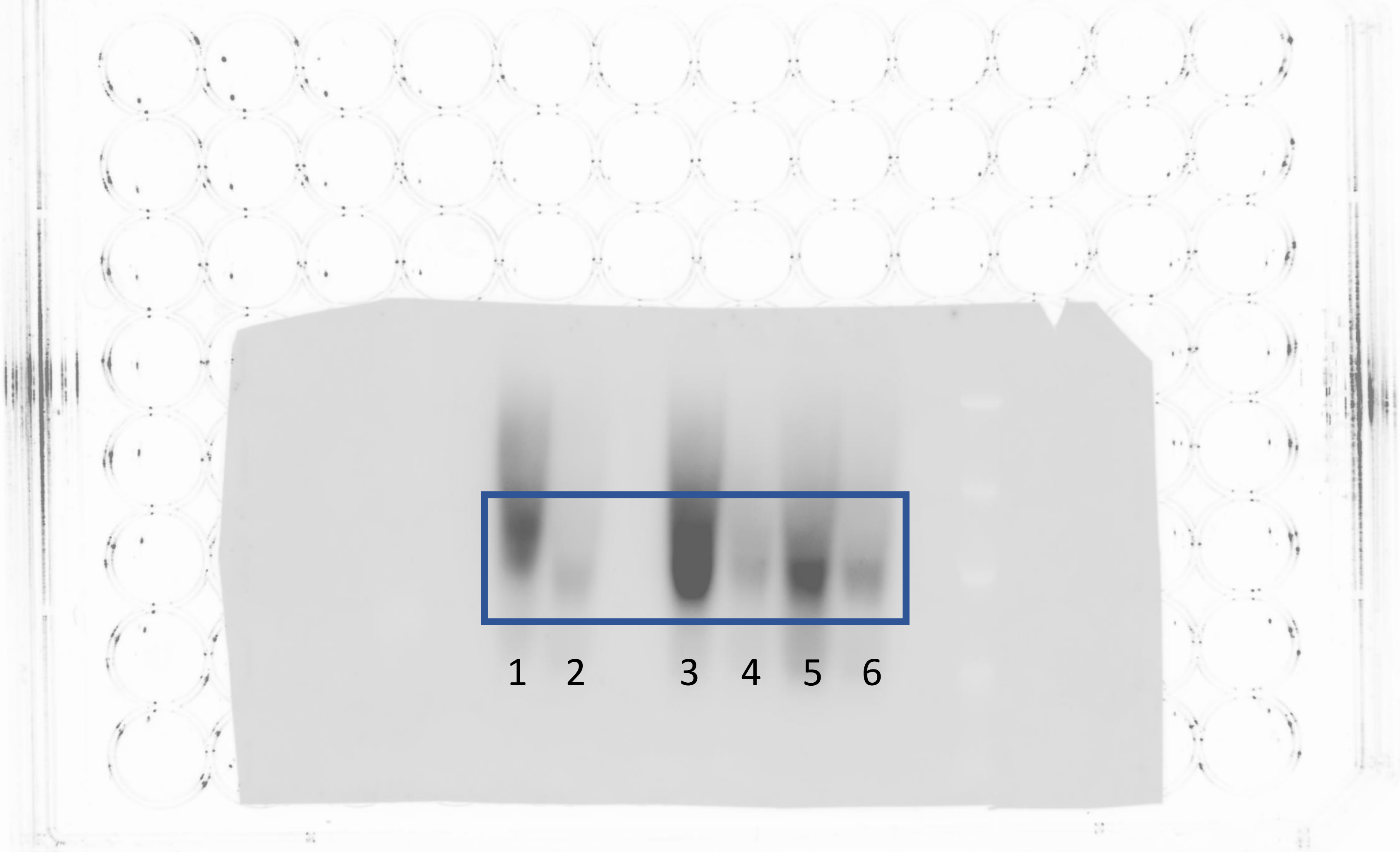

Fig.4.A EXP1 Chk1 (showing overlay)

Lane no.	1	2	3	4	5	6
**Sample**	U937 (3 h)		U937 (48 h)			
	ctrl		ctrl		AraC 100	
siRNA Chk1	-	+	-	+	-	+

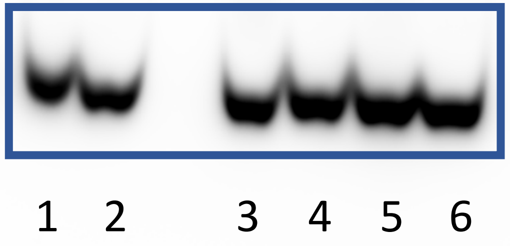

Fig.4.A EXP1 beta-actin

Lane no.	1	2	3	4	5	6
**Sample**	U937 (3 h)		U937 (48 h)			
	ctrl		ctrl		AraC 100	
siRNA Chk1	-	+	-	+	-	+

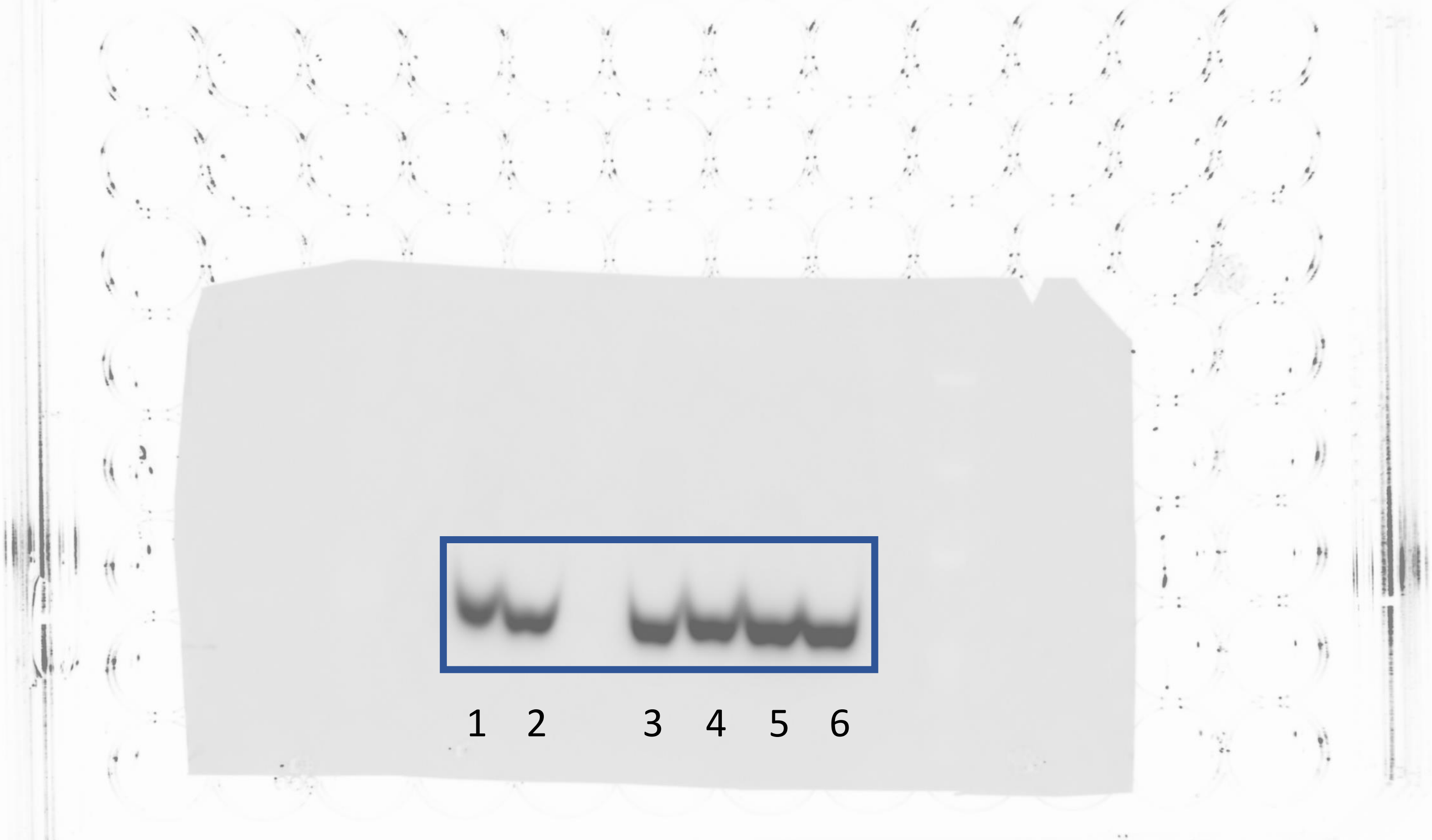

Fig.4.A EXP1 beta-actin (showing overlay)

Lane no.	1	2	3	4	5	6
**Sample**	U937 (3 h)		U937 (48 h)			
	ctrl		ctrl		AraC 100	
siRNA Chk1	-	+	-	+	-	+

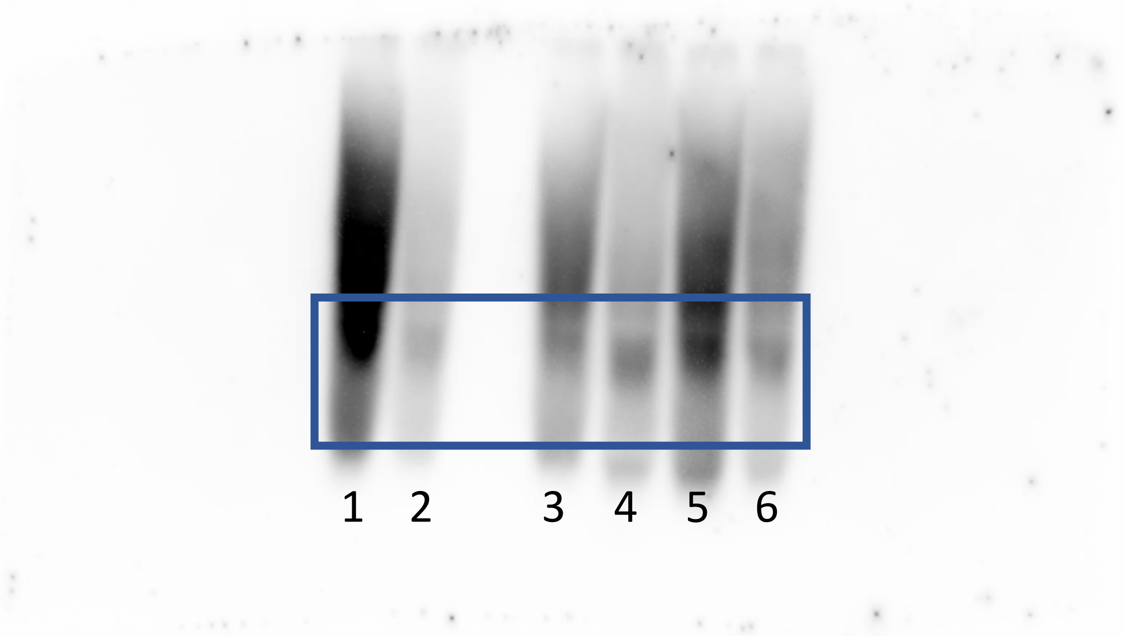

Fig.4.A EXP2 Chk1

Lane no.	1	2	3	4	5	6
**Sample**	U937 (3 h)		U937 (48 h)			
	ctrl		ctrl		AraC 100	
siRNA Chk1	-	+	-	+	-	+

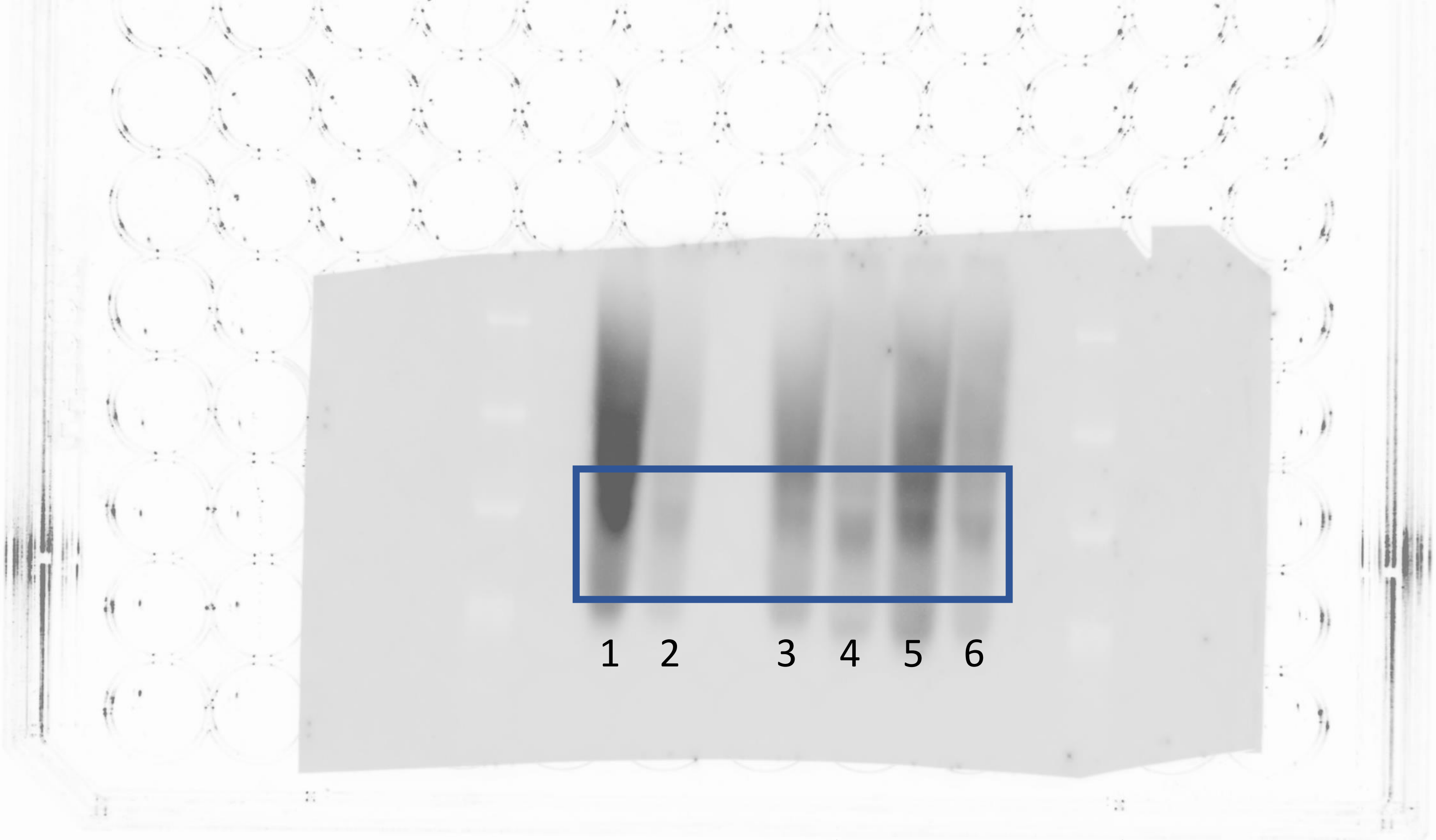

Fig.4.A EXP2 Chk1 (showing overlay)

**Lane no.**	**1**	**2**	**3**	**4**	**5**	**6**
**Sample**	U937 (3 h)		U937 (48 h)			
	ctrl		ctrl		AraC 100	
siRNA Chk1	-	+	-	+	-	+

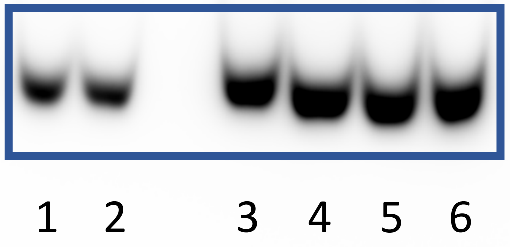

Fig.4.A EXP2 beta-actin

Lane no.	1	2	3	4	5	6
**Sample**	U937 (3 h)		U937 (48 h)			
	ctrl		ctrl		AraC 100	
siRNA Chk1	-	+	-	+	-	+

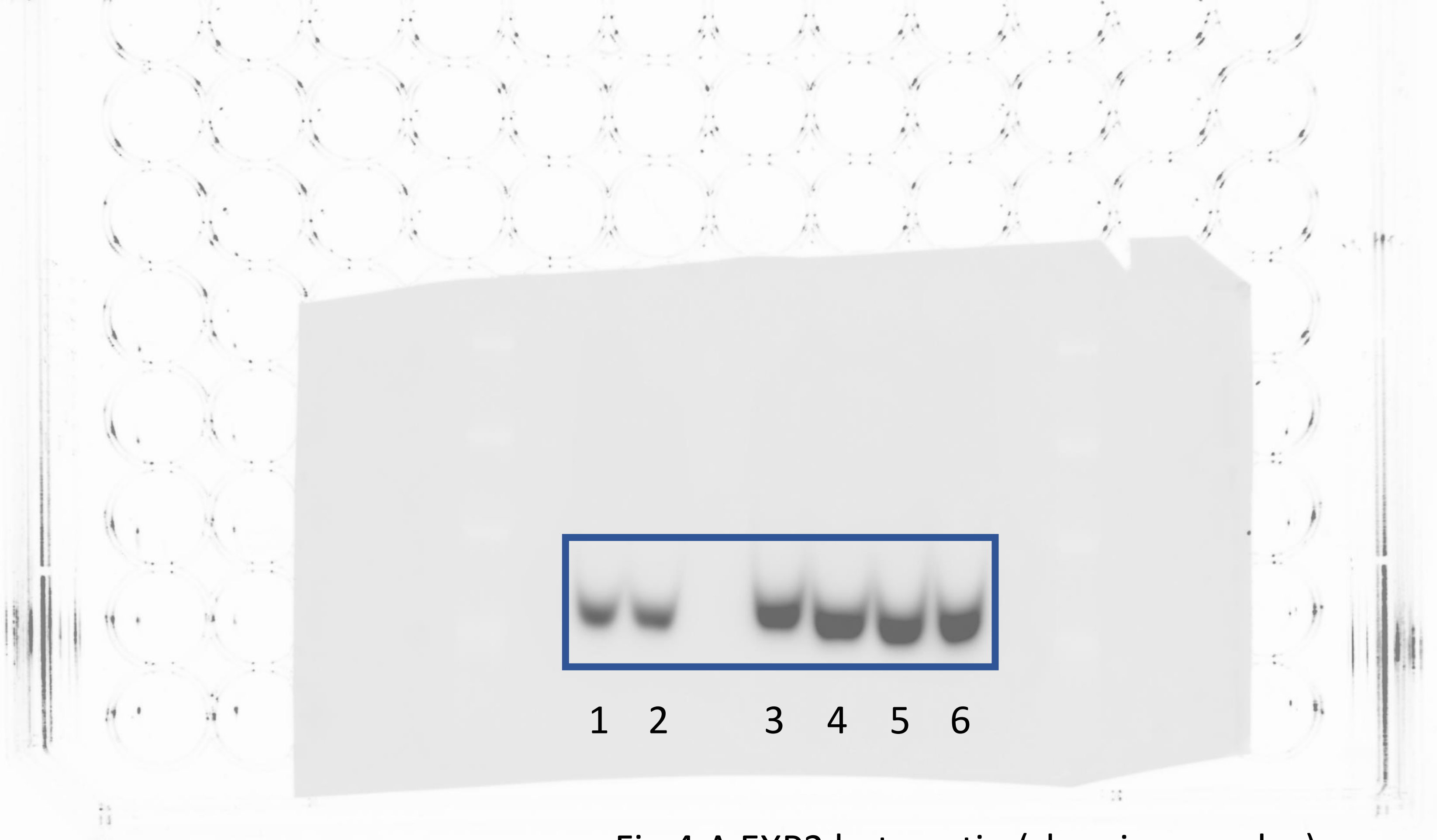

Fig.4.A EXP2 beta-actin (showing overlay)

Lane no.	1	2	3	4	5	6
**Sample**	U937 (3 h)		U937 (48 h)			
	ctrl		ctrl		AraC 100	
siRNA Chk1	-	+	-	+	-	+

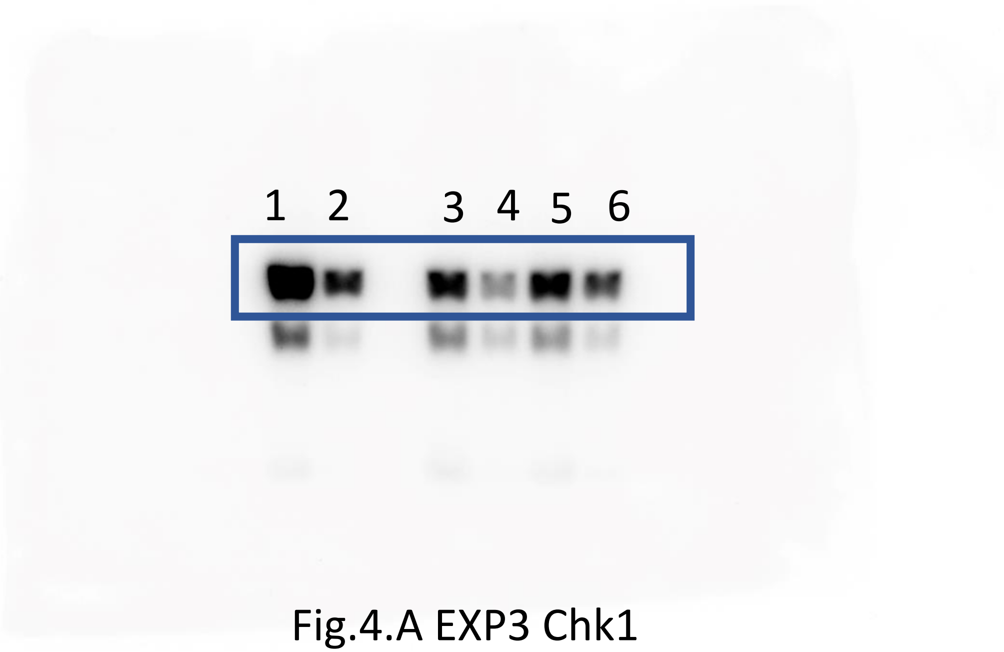

Fig.4.A EXP3 Chk1

Lane no.	1	2	3	4	5	6
**Sample**	U937 (3 h)		U937 (48 h)			
	ctrl		ctrl		AraC 100	
siRNA Chk1	-	+	-	+	-	+

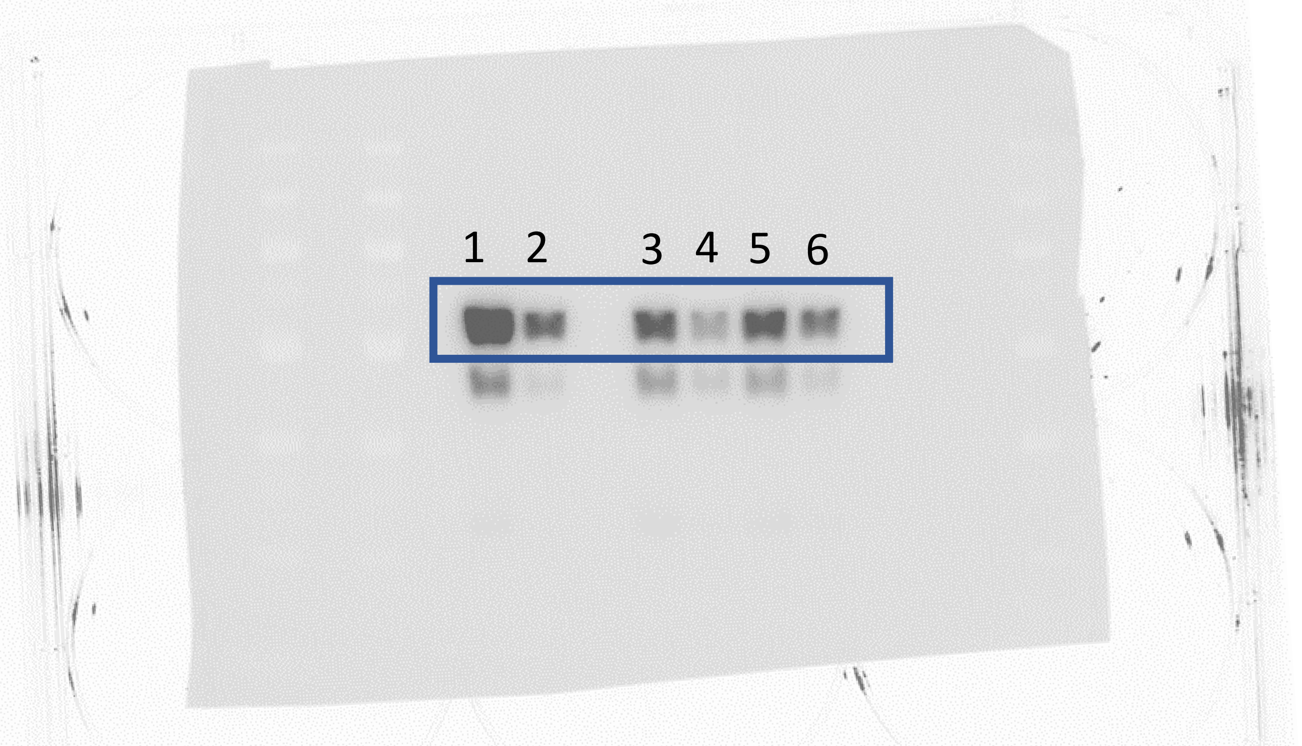

Fig.4.A EXP3 Chk1 (showing overlay)

**Lane no.**	**1**	**2**	**3**	**4**	**5**	**6**
**Sample**	U937 (3 h)		U937 (48 h)			
	ctrl		ctrl		AraC 100	
siRNA Chk1	-	+	-	+	-	+

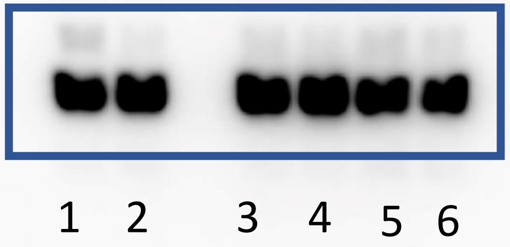

Fig.4.A EXP3 beta-actin (imaging mode: single)

**Lane no.**	**1**	**2**	**3**	**4**	**5**	**6**
**Sample**	U937 (3 h)		U937 (48 h)			
	ctrl		ctrl		AraC 100	
siRNA Chk1	-	+	-	+	-	+

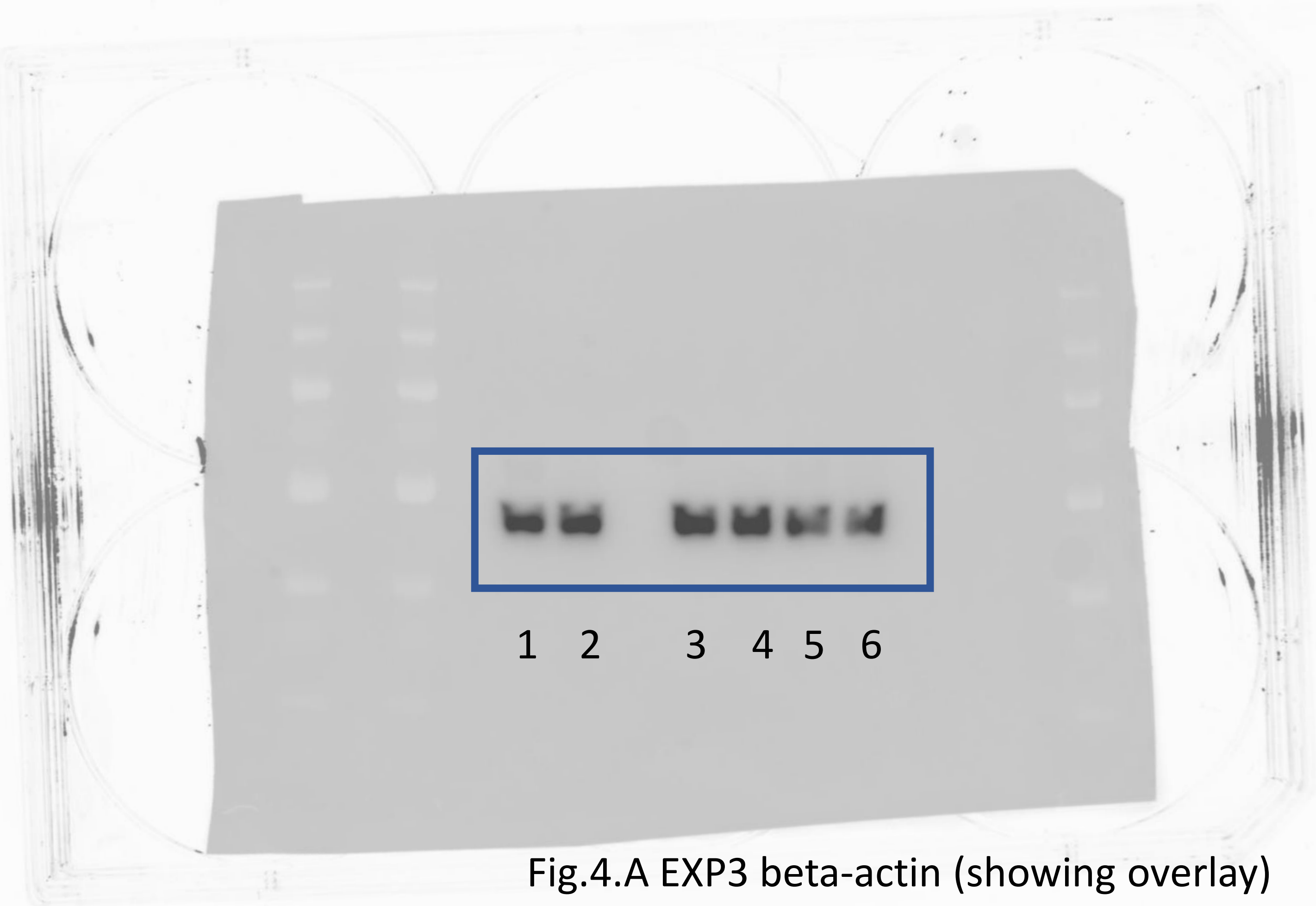

Fig.4.A EXP3 beta-actin (showing overlay)

Lane no.	1	2	3	4	5	6
**Sample**	U937 (3 h)		U937 (48 h)			
	ctrl		ctrl		AraC 100	
siRNA Chk1	-	+	-	+	-	+

For Figure 5.

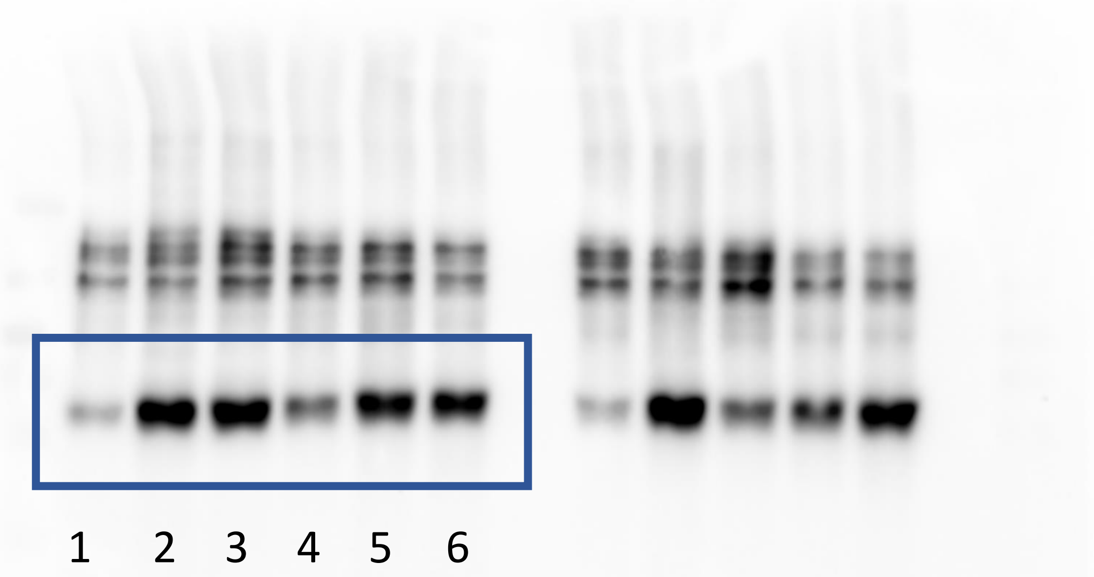

Fig.5.C WB1 pChk1

Lane no.	1	2	3	4	5	6
Sample	ctrl	AIC	Bq	AraC 10	AraC 100	AraC 1000

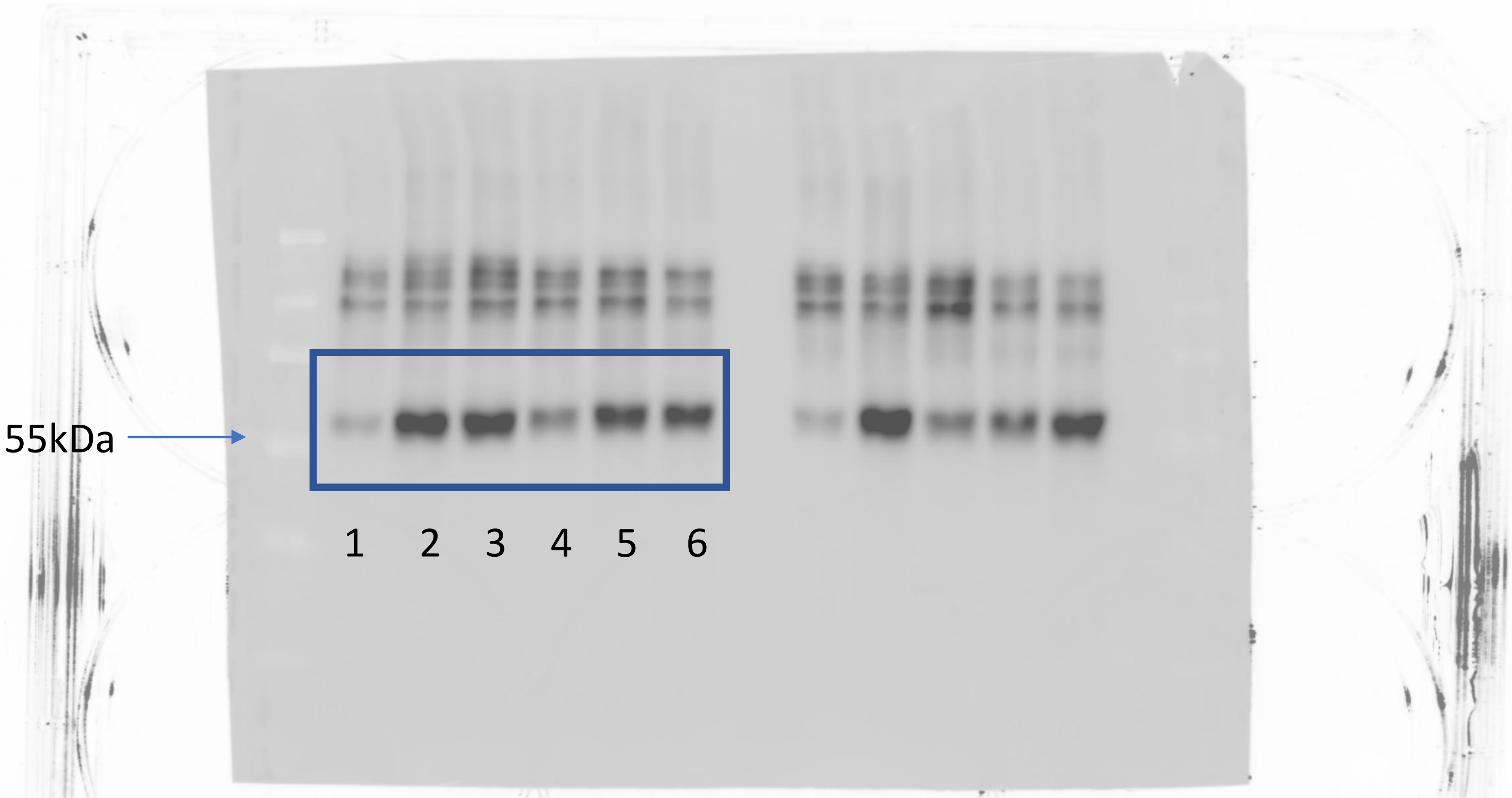

Fig.5.C WB1 pChk1 (showing overlay)

Lane no.	1	2	3	4	5	6
Sample	ctrl	AIC	Bq	AraC 10	AraC 100	AraC 1000

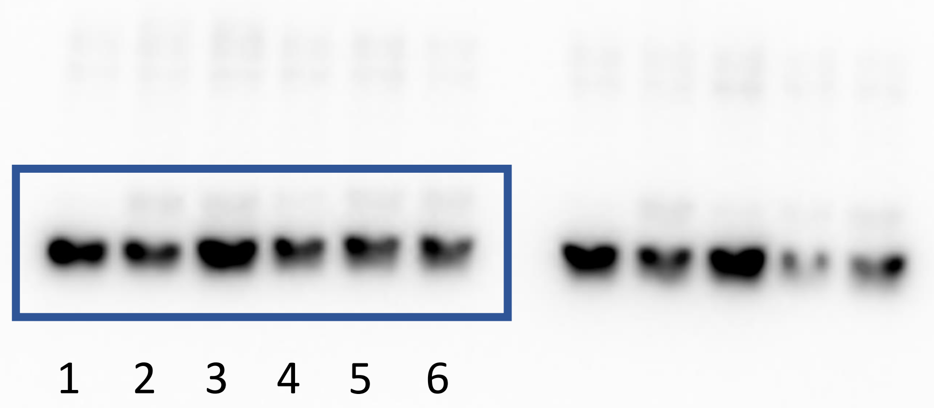

Fig.5.C WB1 beta-actin

Lane no.	1	2	3	4	5	6
Sample	ctrl	AIC	Bq	AraC 10	AraC 100	AraC 1000

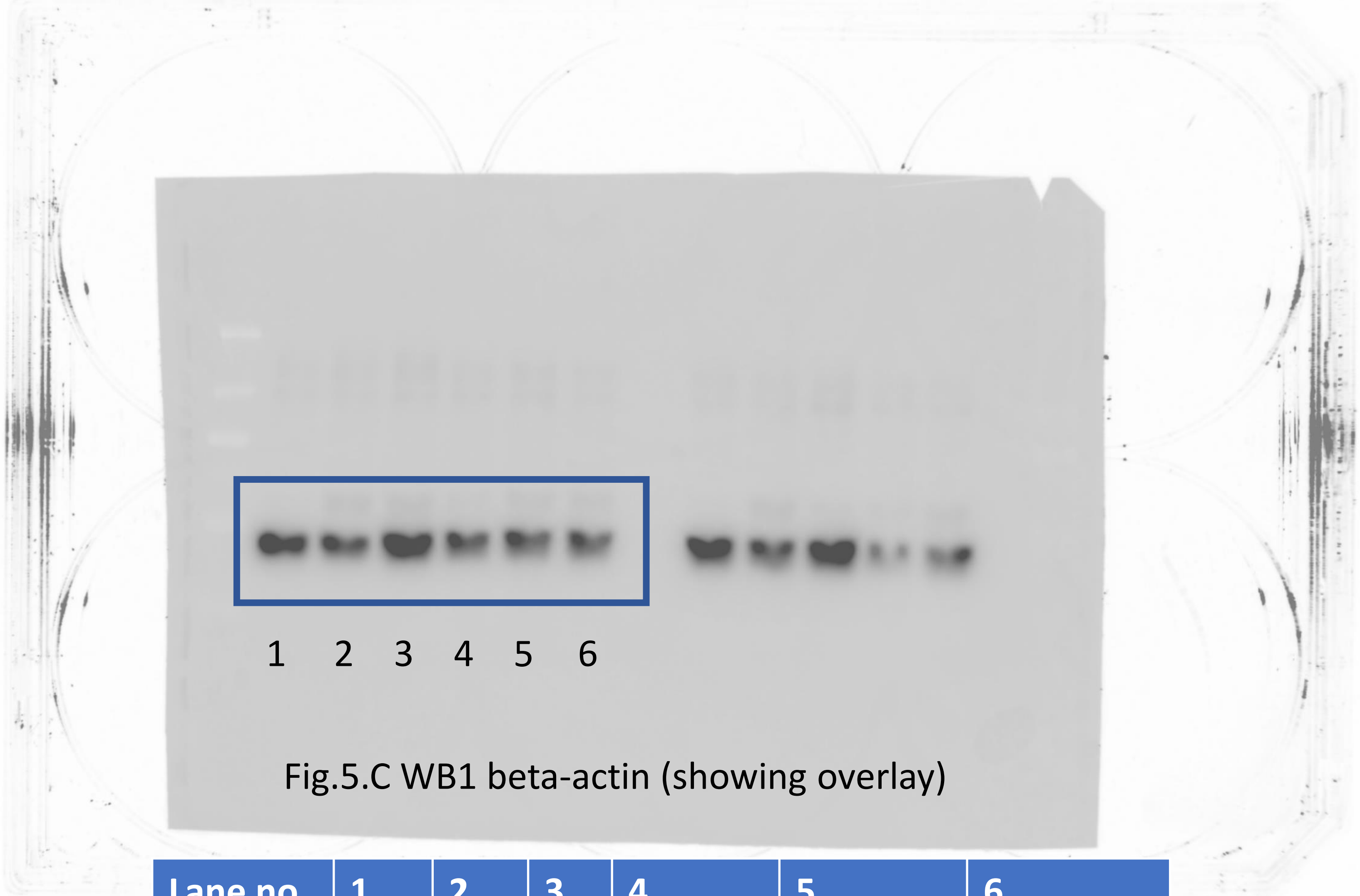

Fig.5.C WB1 beta-actin (showing overlay)

Lane no.	1	2	3	4	5	6
Sample	ctrl	AIC	Bq	AraC 10	AraC 100	AraC 1000

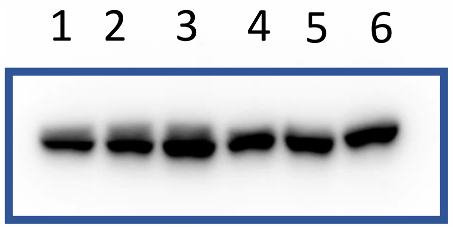

Fig.5.C WB2 Chk1

Lane no.	1	2	3	4	5	6
Sample	ctrl	AIC	Bq	AraC 10	AraC 100	AraC 1000

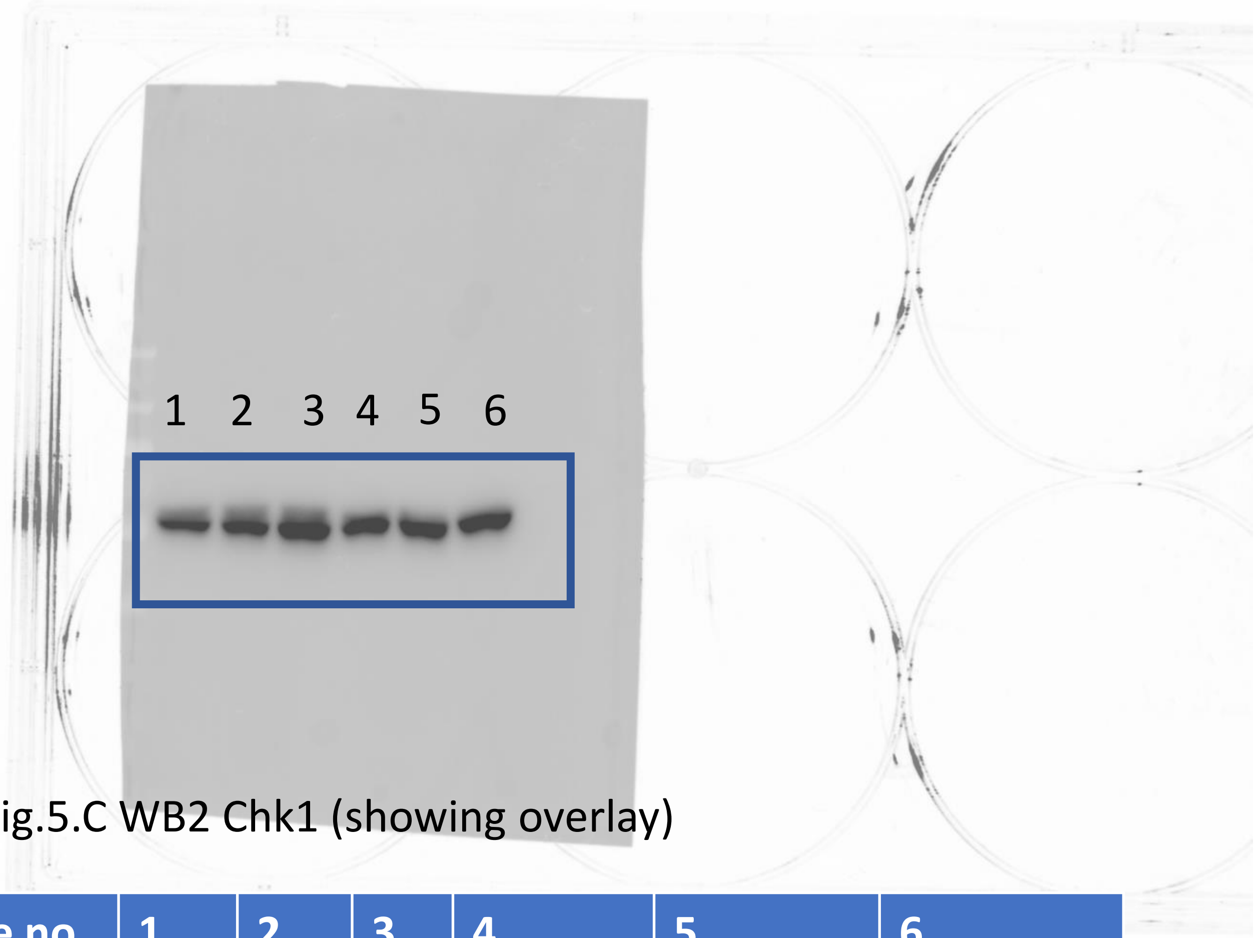

Fig.5.C WB2 Chk1 (showing overlay)

Lane no.	1	2	3	4	5	6
Sample	ctrl	AIC	Bq	AraC 10	AraC 100	AraC 1000

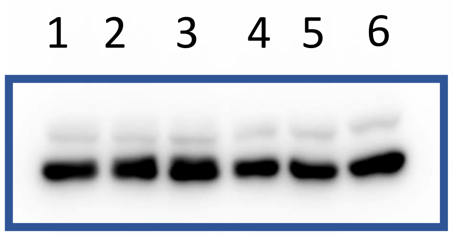

Fig.5.C WB2 beta-actin

Lane no.	1	2	3	4	5	6
Sample	ctrl	AIC	Bq	AraC 10	AraC 100	AraC 1000

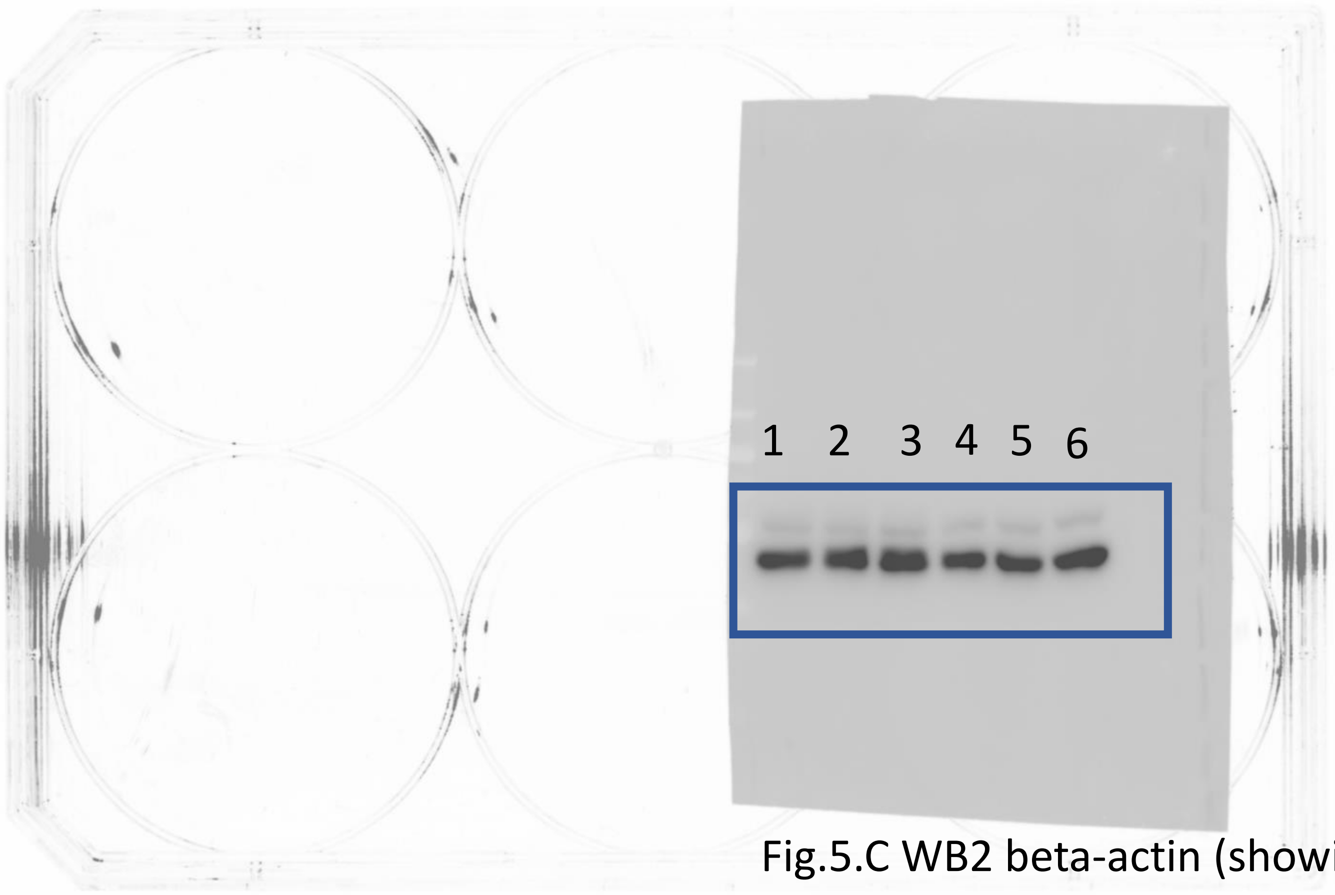

Fig.5.C WB2 beta-actin (showing overlay)

Lane no.	1	2	3	4	5	6
Sample	ctrl	AIC	Bq	AraC 10	AraC 100	AraC 1000

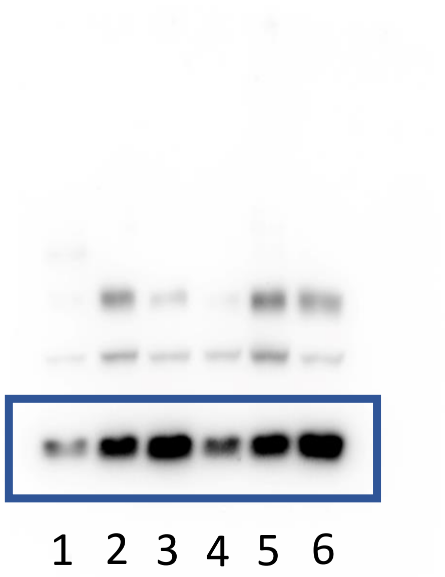

Fig.5.C WB3 pCDC2

Lane no.	1	2	3	4	5	6
Sample	ctrl	AIC	Bq	AraC 10	AraC 100	AraC 1000

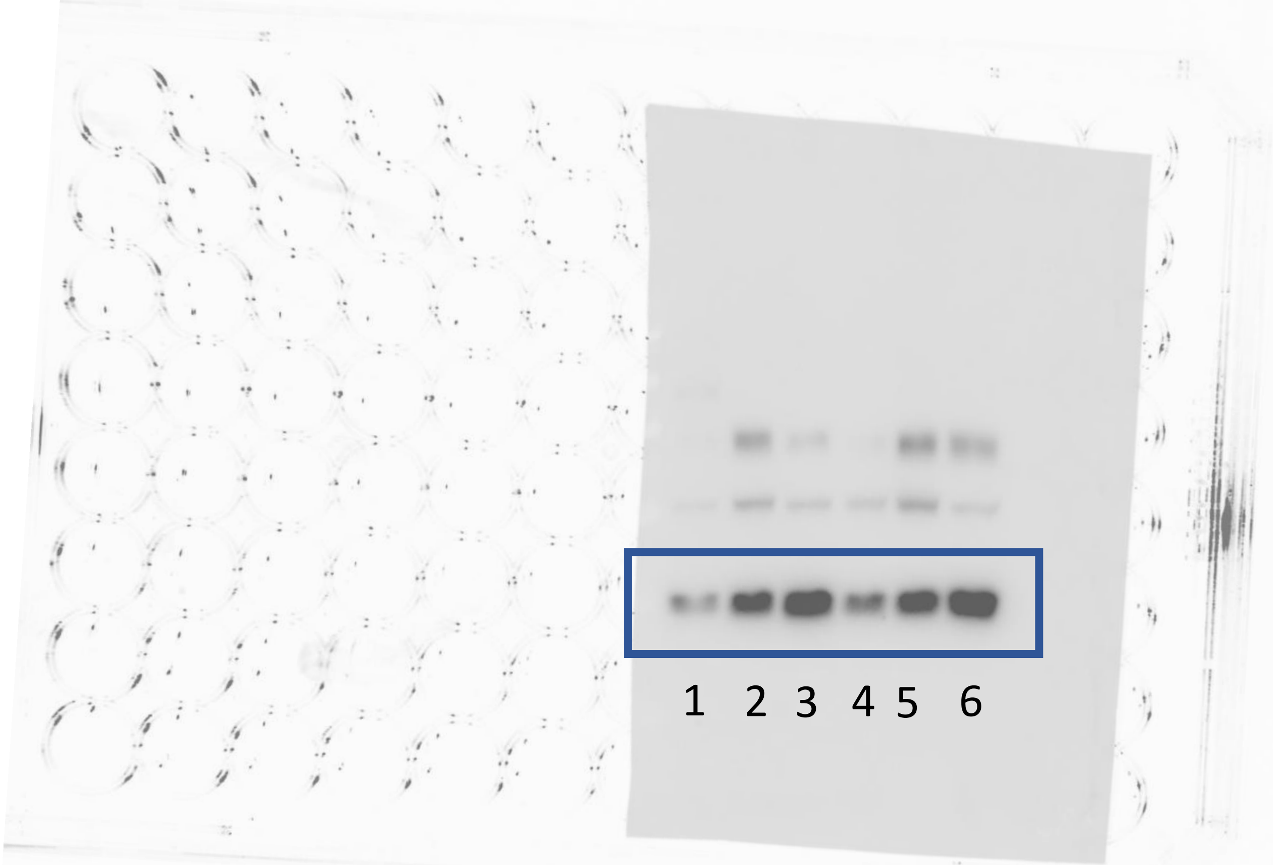

Fig.5.C WB3 pCDC2 (showing overlay)

Lane no.	1	2	3	4	5	6
Sample	ctrl	AIC	Bq	AraC 10	AraC 100	AraC 1000

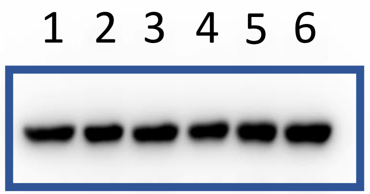

Fig.5.C WB3 beta-actin

Lane no.	1	2	3	4	5	6
Sample	ctrl	AIC	Bq	AraC 10	AraC 100	AraC 1000

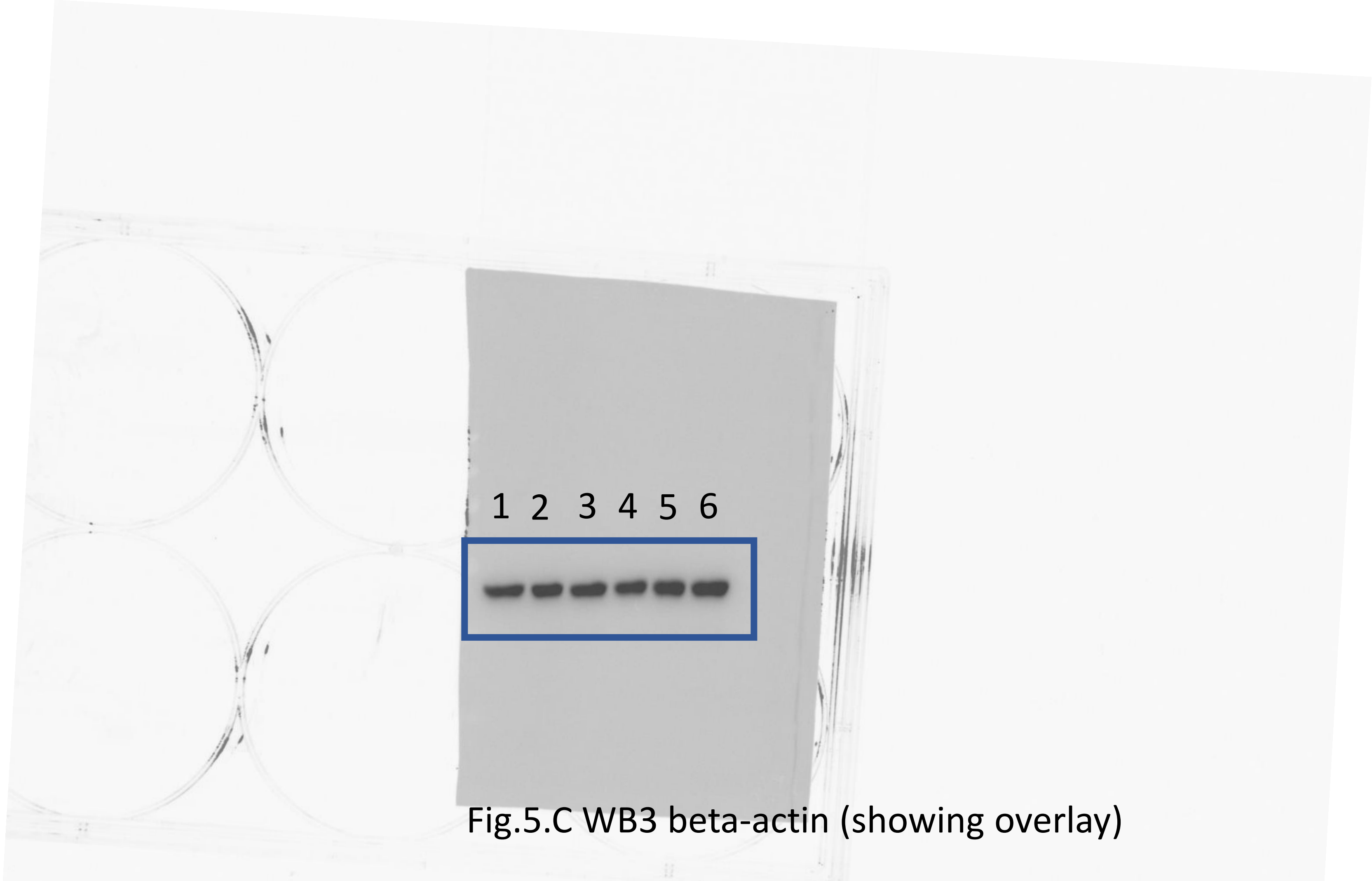

Fig.5.C WB3 beta-actin (showing overlay)

Lane no.	1	2	3	4	5	6
Sample	ctrl	AIC	Bq	AraC 10	AraC 100	AraC 1000

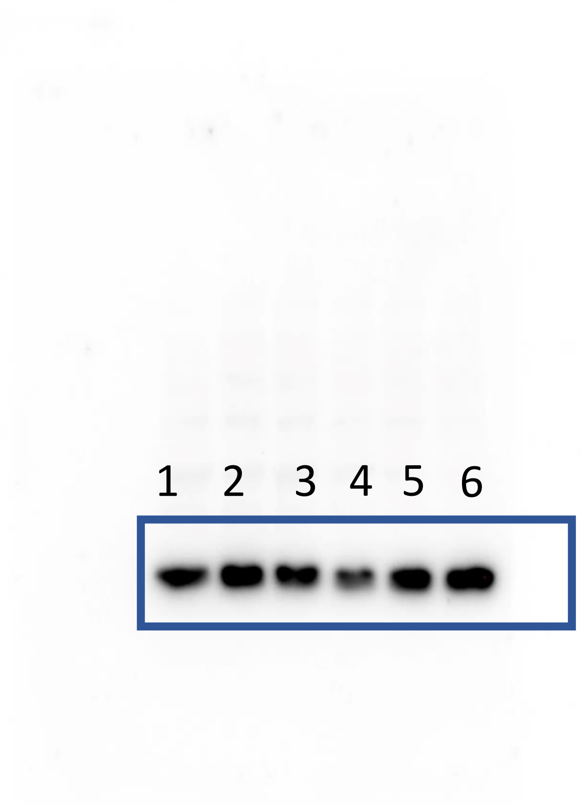

Fig.5.C WB4 CDC2

Lane no.	1	2	3	4	5	6
Sample	ctrl	AIC	Bq	AraC 10	AraC 100	AraC 1000

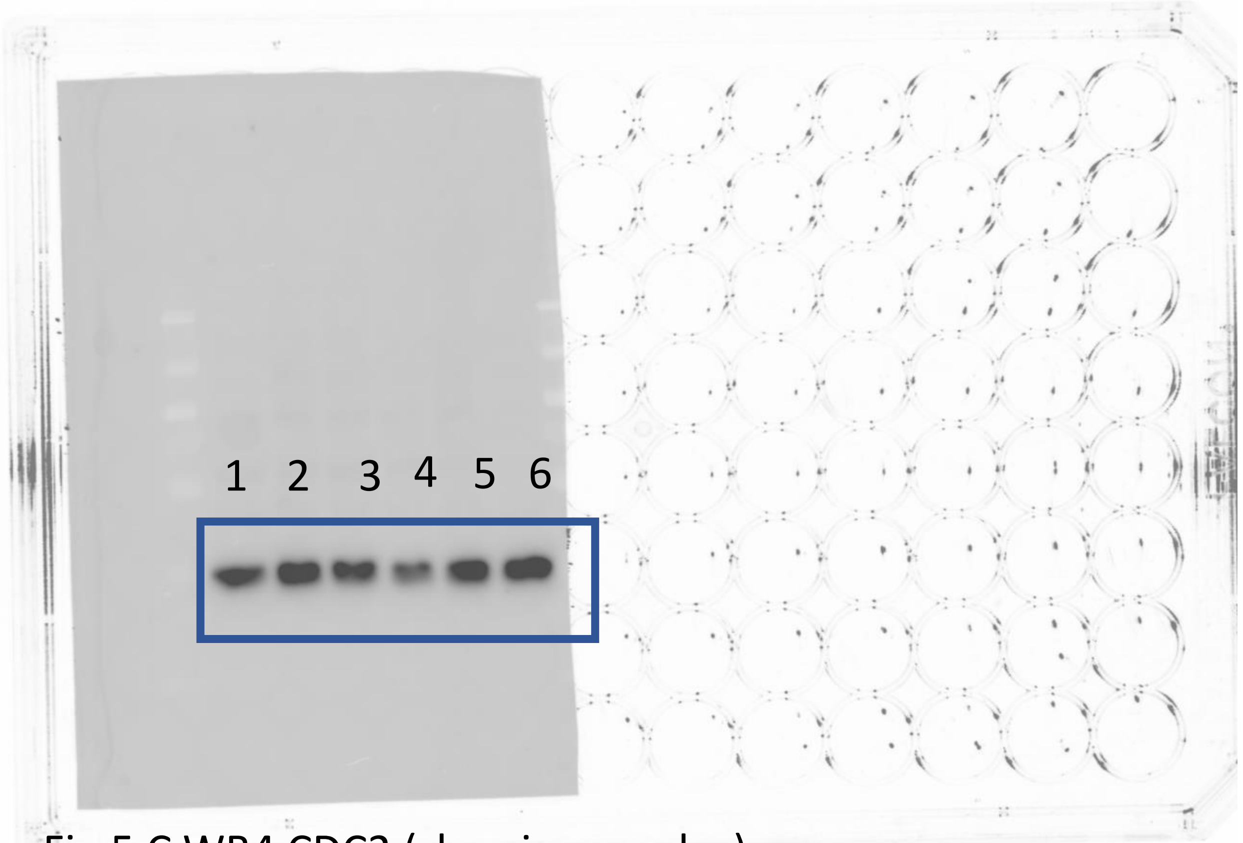

Fig.5.C WB4 CDC2 (showing overlay)

Lane no.	1	2	3	4	5	6
Sample	ctrl	AIC	Bq	AraC 10	AraC 100	AraC 1000

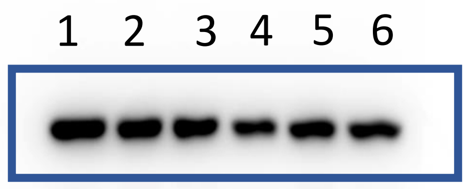

Fig.5.C WB4 beta-actin

Lane no.	1	2	3	4	5	6
Sample	ctrl	AIC	Bq	AraC 10	AraC 100	AraC 1000

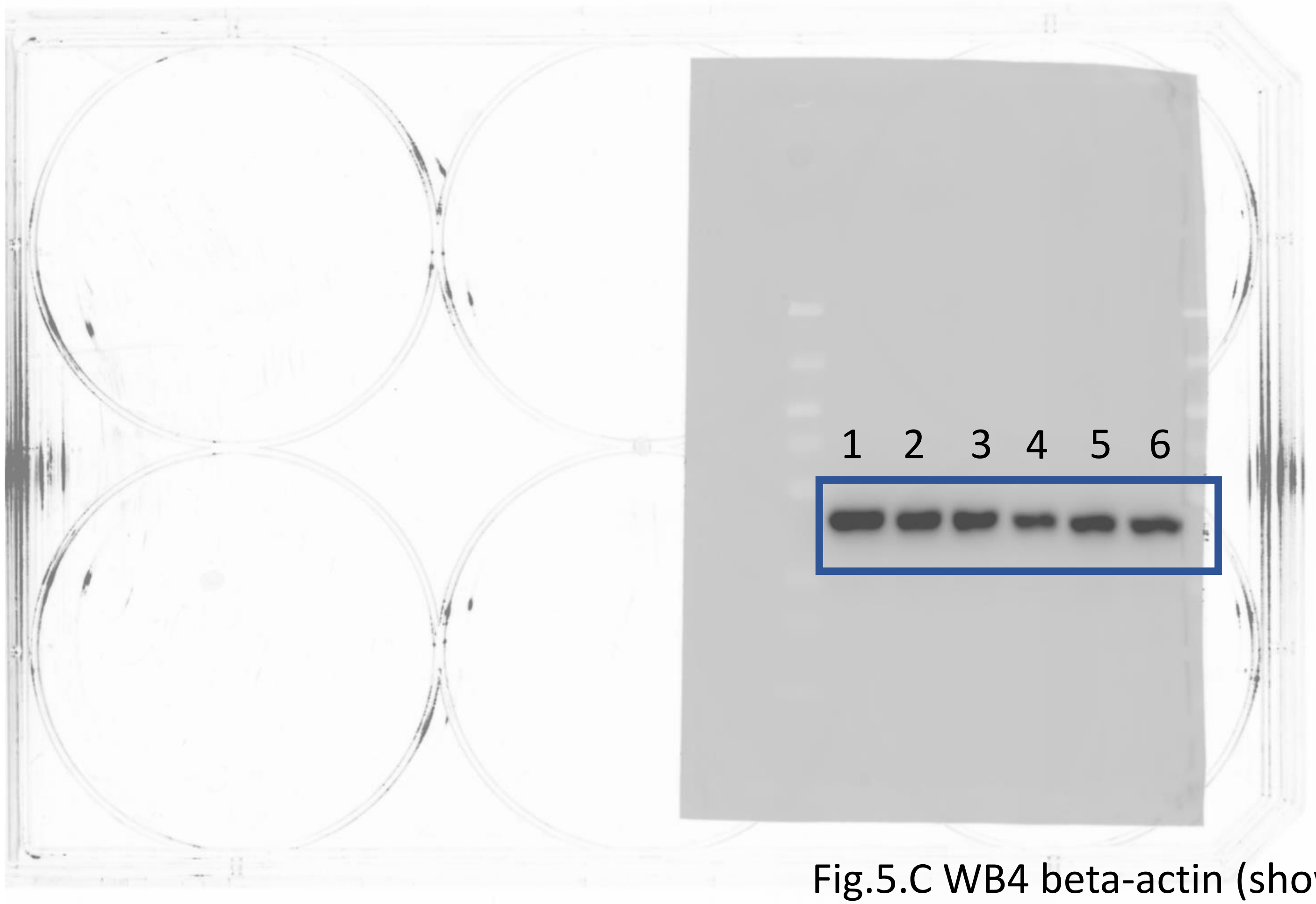

Fig.5.C WB4 beta-actin (showing overlay)

Lane no.	1	2	3	4	5	6
Sample	ctrl	AIC	Bq	AraC 10	AraC 100	AraC 1000
