# Supplementary material for: Cytarabine-induced differentiation of AML cells depends on Chk1 activation and shares the mechanism with inhibitors of DHODH and pyrimidine synthesis
Source: Sci Rep. 2022 Jul 5;12:11344. doi: 10.1038/s41598-022-15520-z (PMC9256737; doi:10.1038/s41598-022-15520-z)
Supplement: Supplementary file 2 — Supplementary Information 2. [file 41598_2022_15520_MOESM2_ESM.pdf]

Cytarabine-induced differentiation of AML cells depends on Chk1 activation and shares the mechanism with inhibitors of DHODH and pyrimidine synthesis

Barbara Tomic, Tomislav Smoljo, Hrvoje Lalic, Vilma Dembitz, Josip Batinic, Drago Batinic, Antonio Bedalov, Dora Visnjic

Supplementary Figure 1

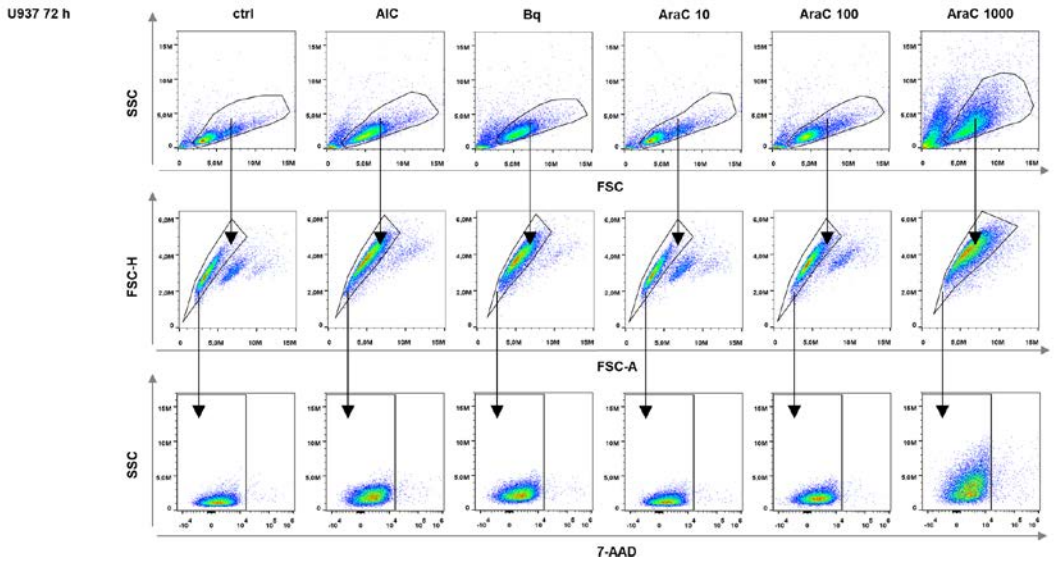

Supplementary Figure 1. Flow cytometry gating strategy. Dot plots of representative samples shown in Figure 1C.

Supplementary Figure 2

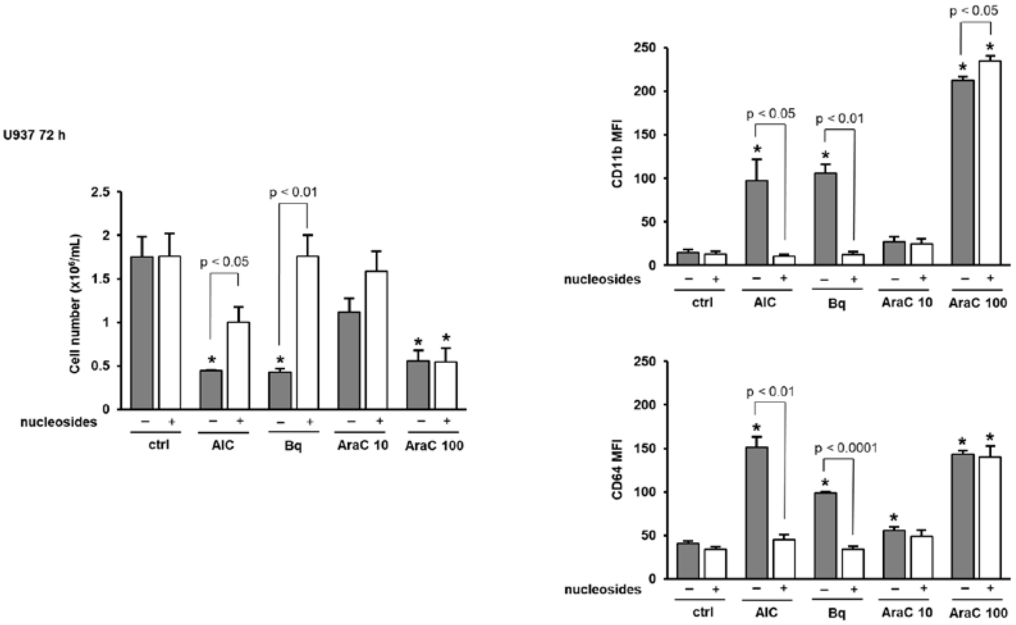

Supplementary Figure 2. Cytarabine induces differentiation that is not abolished by the addition of nucleosides. U937 cells were incubated with AICAr (0.2 mM), brequinar (Bq) (0.5 µM) and AraC (10 and 100 nM). Nucleosides (1x) were added 15 minutes after addition of agents. The number of viable cells was determined by trypan blue exclusion. Mean fluorescence intensity (MFI) of CD11b and CD64 was calculated as described under “Methods” section. Results are mean ± S.E. (error bars) of at least three independent experiments. *, $p < 0.05$ compared with control (ctrl).

Supplementary Figure 3

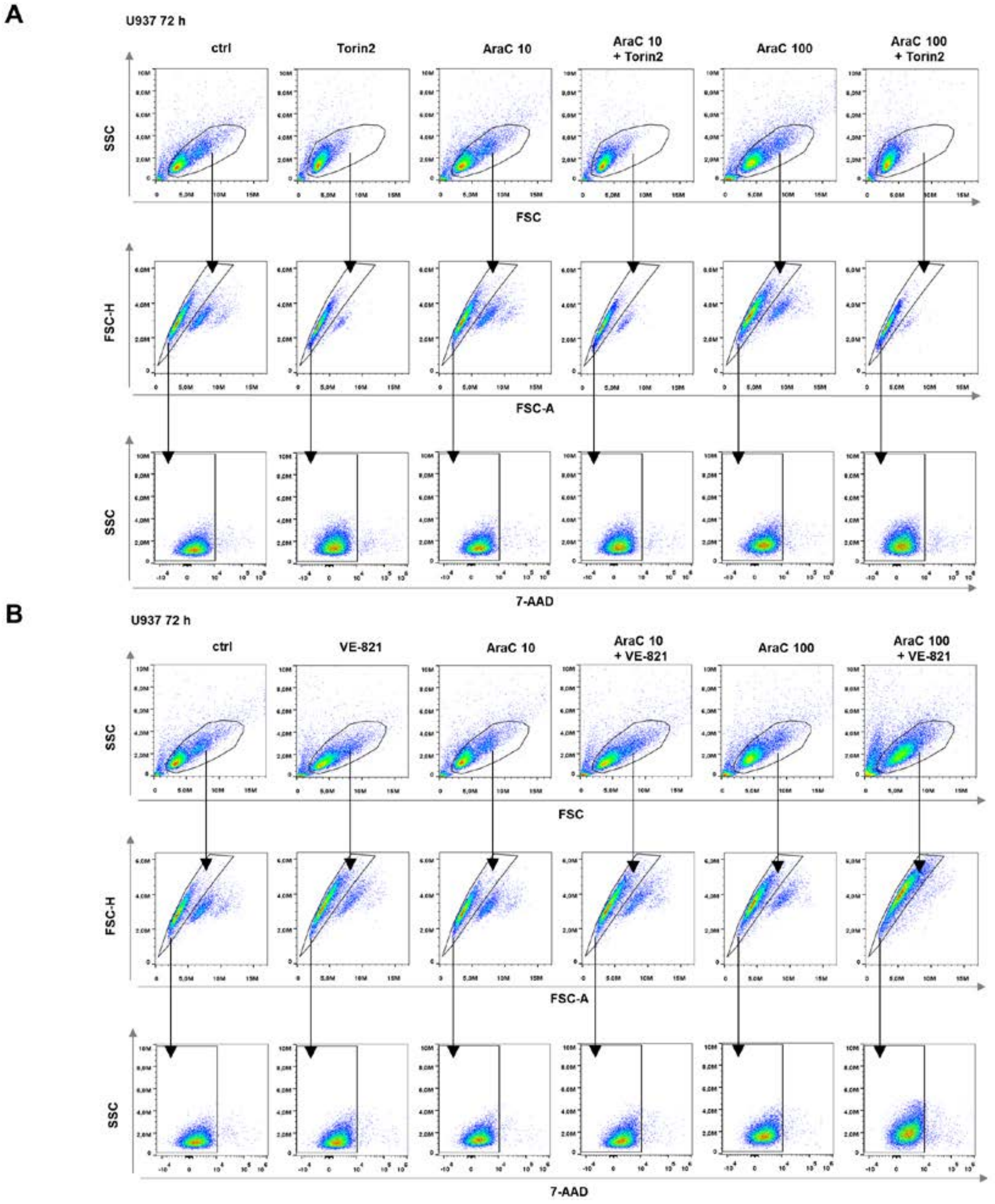

Supplementary Figure 3. Flow cytometry gating strategy. (A) Dot plots of representative samples shown in Figure 3B. (B) Dot plots of representative samples shown in Figure 3C.

Supplementary Figure 4

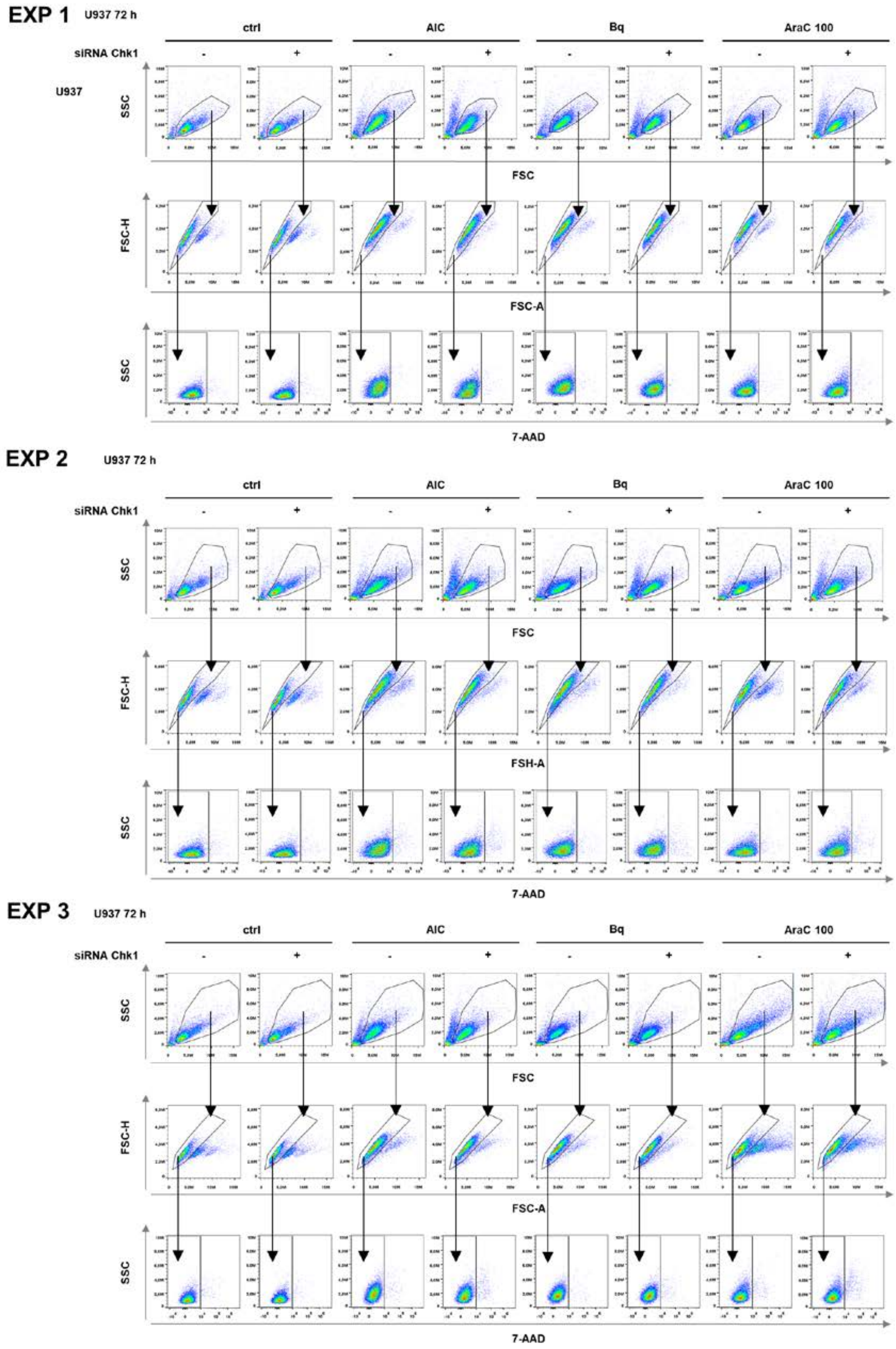

Supplementary Figure 4. Flow cytometry gating strategy. Dot plots of samples shown in Figure 4B.

Supplementary Figure 5

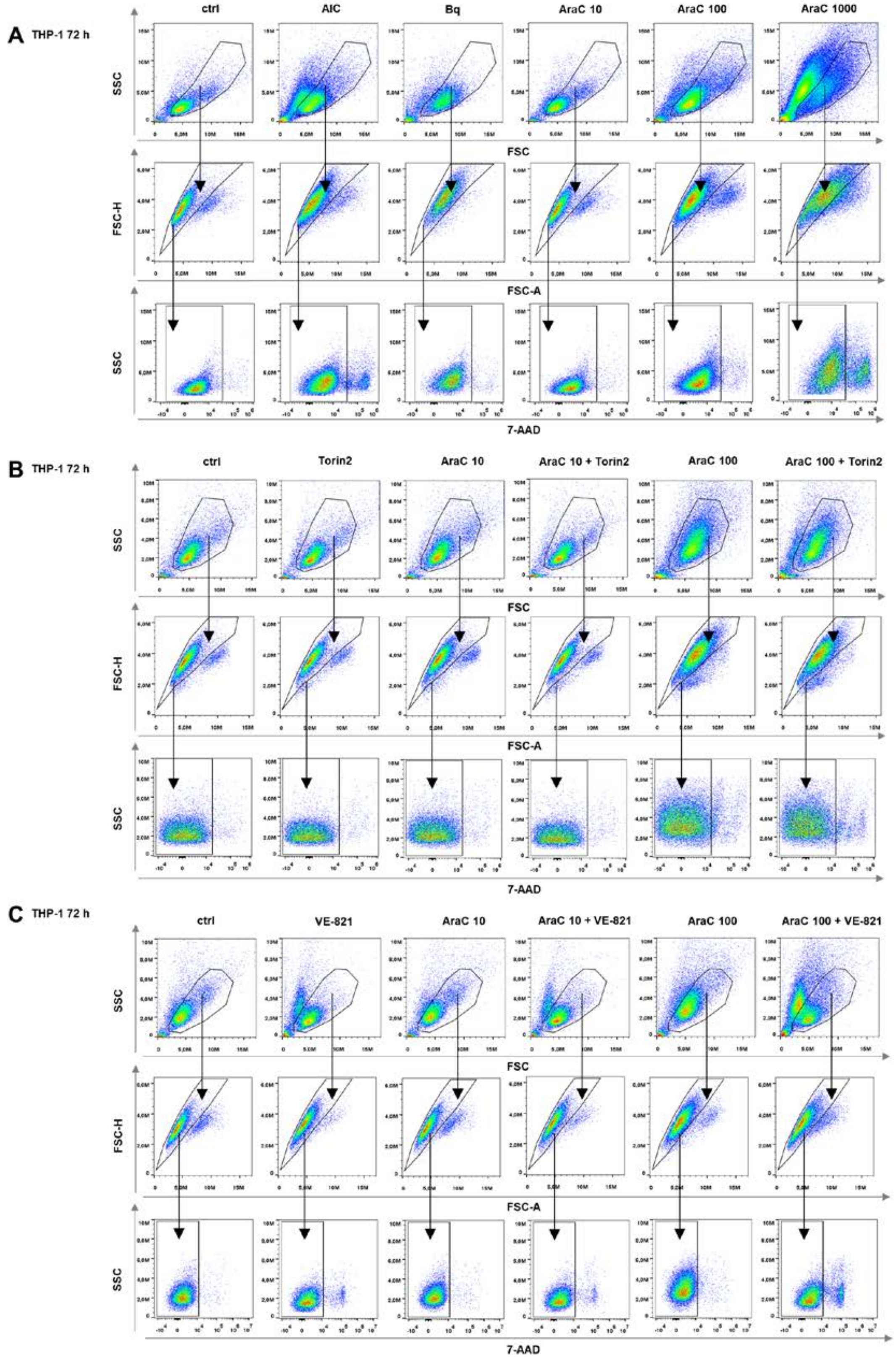

Supplementary Figure 5. Flow cytometry gating strategy. (A) Dot plots of representative samples shown in Figure 5B. (B) Dot plots of representative samples shown in Figure 5D. (C) Dot plots of representative samples shown in Figure 5E.

Supplementary Figure 6

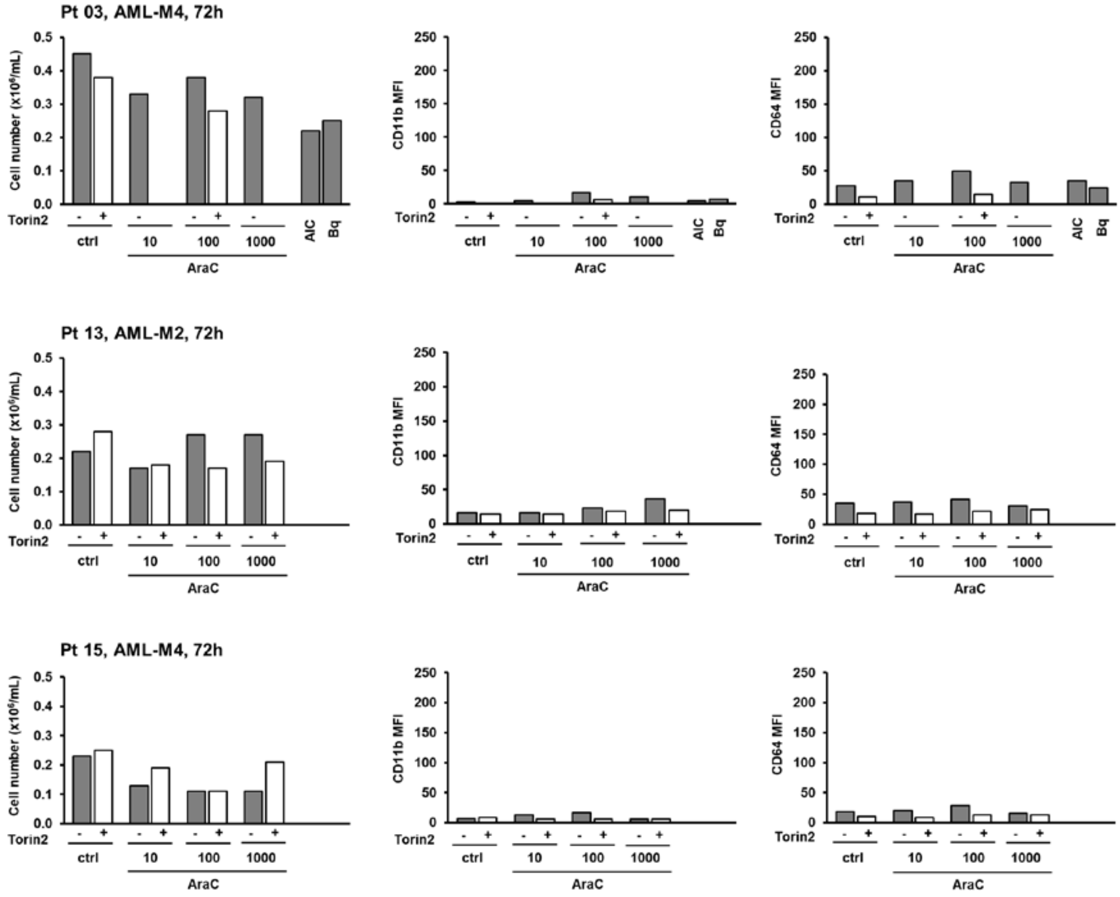

Supplementary Figure 6. Cytarabine had no effects on the expression of differentiation markers in primary AML samples that were unresponsive to pyrimidine synthesis inhibitors. Non-adherent mononuclear cells from bone marrow samples (Pt 03, AML-M4, *FLT3-ITD*, *RUNX1/RUNX1T1*; Pt 13, AML-M2, *RUNX1/RUNX1T1*; Pt 15, AML-M4, *FLT3-ITD*, *RUNX1/RUNX1T1*) were plated at concentration 0.4×10^6/mL in medium supplemented with 50 ng/mL IL-3, IL-6, SCF and FLT3L and incubated with AraC (10, 100, 1000 nM) with or without Torin2 (10 nM) for 72 h. AICAr (AIC) (0.2 mM) and brequinar (Bq) (0.5 µM) treated cells were used as positive controls. The number of viable cells was determined by trypan blue exclusion. Mean fluorescence intensity (MFI) of CD11b and CD64 was calculated as described under "Methods" section.

Supplementary Table 1. Patient characteristics

Patients	WBC	Blasts in BM (%)	Blasts in PB (%)	Cytology (AML subtype)	Immunophenotype (% total cells)	Cytogenetics	Molecular markers
**Responders**							
7	30.5	34	57	FAB M4	myeloblastic (36%), monocytic (30%)	46, XX	FLT3-ITD, NPM1
14	25.8	20	40	FAB M2	immature myeloid (32%), myeloblastic (25%)	46, XY	negative: FLT3, NPM1, BCR-ABL
**Non-responders**							
3	13.3	50	51	FAB M4	myeloblast 36%	45, X, -X, t(3;14)(q27;q10), t(8;21)(q22;q22) / 46, XX;	FLT3-ITD, RUNX1/RUNX1T1
13	8.2	28	19	FAB M2	myeloblastic 25%	45, X, -Y, t(8;21)(q22;q22) / 46,XY;	RUNX1/RUNX1T1
15	26.8	30	56	FAB M4	immature myeloid (35%)	45, X, -Y, t(8;21)(q22;q22) / 90, XX, -Y, -Y, t(8;21)(q22;q22)x2 / 46, XY	FLT3-ITD, RUNX1/RUNX1T1

Supplementary Table 2. Reagents and resources used

REAGENT or RESOURCE	SOURCE	IDENTIFIER
**Antibodies**		
IgG1-FITC (clone 679.1Mc7)	Immunotech BeckmanCoulter, Marseille, France	A07795, RRID:AB_2832964
IgG1-APC (MOPC-21)	BD Biosciences, San Jose, CA, USA	555751, RRID:AB_398613
IgG1-PerCP (559425)	BD Biosciences, San Jose, CA, USA	559425, RRID:AB_397240
CD11b-FITC (clone Bear1)	Immunotech BeckmanCoulter, Marseille, France	IM0530, RRID:AB_130987
CD64-FITC (clone 22)	Immunotech BeckmanCoulter, Marseille, France	B49185
CD34-APC (8G12)	BD Biosciences, San Jose, CA, USA	345804, RRID:AB_2686894
CD45-PerCP (2D1)	BD Biosciences, San Jose, CA, USA	345809, RRID:AB_2868830
Chk1 Mouse mAb (2G1D5)	Cell Signaling Technology, Beverly, MA, USA	2360, RRID:AB_2080320
p-Chk1 (S345) Rabbit mAb (133D3)	Cell Signaling Technology, Beverly, MA, USA	2348, RRID:AB_331212
cdc2 Mouse mAb (POH1)	Cell Signaling Technology, Beverly, MA, USA	9116 RRID:RRID:AB_2074795
Phospho-cdc2 (Tyr15) antibody	Cell Signaling Technology, Beverly, MA, USA	9111, RRID:AB_331460
Monoclonal anti-β-actin antibody produced in mouse (clone AC-15)	Sigma, St. Louis, MO, USA	A5441, RRID:AB_476744
Anti-mouse IgG, HRP-linked antibody	Cell Signaling Technology, Beverly, MA, USA	7076, RRID:AB_330924
Anti-rabbit IgG, HRP-linked antibody	Cell Signaling Technology, Beverly, MA, USA	7074, RRID:AB_2099233
**Annexin A5-FITC kit**	Immunotech BeckmanCoulter, Marseille, France	IM3546, RRID:AB_130885
**siRNA transfection**		
ON-TARGETplus SMARTpool Human CHEK1 siRNA	Dharmacon, Lafayette, CO, USA	L-003255-00
ON-TARGETplus Non-targeting Control Pool	Dharmacon, Lafayette, CO, USA	D-001810-10
Neon Transfection System 100 µl kit	Life Technologies, Carlsbad, CA, USA	MPK10025
**Chemicals and peptides**		
Cytarabine	Sigma, St. Louis, MO, USA	C1786
AICAr	Sigma, St. Louis, MO, USA	A9978
Brequinar	Sigma, St. Louis, MO, USA	SML0113
Torin2	Sigma, St. Louis, MO, USA	SML1224
VE821	Sigma, St. Louis, MO, USA	SML1415
Z-VAD-FMK	Adooq Bioscience LLC, CA, USA	A12373

Dimethyl sulfoxide (DMSO)	Honeywell, Muskegon, MI, USA	472301
Phorbol myristate acetate (PMA)	Sigma, St. Louis, MO, USA	P1585
EmbryoMax Nucleosides (100X)	Sigma, St. Louis, MO, USA	ES-008-D
Propidium iodide	Sigma, St. Louis, MO, USA	P4170
Dihydrorhodamine 123 (DHR123)	Thermo Fisher Scientific, Waltham, MA, USA	D632
7-AAD staining solution	Miltenyi Biotec GmbH, Bergisch Gladbach, Germany	130-111-568
Human TruStain FcX™ Fc Receptor Blocking solution	BioLegend, San Diego, CA, USA	422302
NycoPrep 1.077 solution	Axis-Shield PoC AS, Oslo, Norway	1114741
Interleukin-6 human	Sigma, St. Louis, MO, USA	H7416
Interleukin-3 human	Sigma, St. Louis, MO, USA	H7166
FLT3 ligand human	Sigma, St. Louis, MO, USA	H5416
Stem cell factor human	Sigma, St. Louis, MO, USA	H8416
Bovine serum albumin (BSA)	Sigma, St. Louis, MO, USA	A4503
Tween® 20	Sigma, St. Louis, MO, USA	P7949
Acrylamide/bis-acrylamide solution	Sigma, St. Louis, MO, USA	A3574
Igepal CA	Sigma, St. Louis, MO, USA	I3021
Bradford reagent	Sigma, St. Louis, MO, USA	B6916
TGX Stain-Free FastCast Acrylamide Kit, 10%	Bio-Rad Laboratories, Inc., USA	1610183
SuperSignal™ West Pico PLUS Chemiluminescent Substrate	Thermo Fisher Scientific, Waltham, MA, USA	34577
Amersham™ Protran® Western blotting membrane, nitrocellulose	GE Healthcare Life Sciences, Chicago, IL, USA	GE10600007
Microcystin-LR	Enzo, Life Sciences, Farmingdale, NY, USA	ALX-350-012-C100
Phenylmethylsulfonyl fluoride (PMSF)	Sigma, St. Louis, MO, USA	P7626
Cell lysis buffer (10x)	Cell Signaling Technology, Beverly, MA, USA	9803S
ColorBurst™ electrophoresis marker mol wt 8,000-220,000 Da	Sigma, St. Louis, MO, USA	C1992-1VL
PageRuler™ Plus Prestained Protein Ladder, 10 to 250 kDa	Thermo Fisher Scientific, Waltham, MA, USA	26619
Sodium dodecyl sulfate, approx. 95% (SDS)	Sigma, St. Louis, MO, USA	L5750
Glycerol	Sigma, St. Louis, MO, USA	G7893
Bromphenol blue	Sigma, St. Louis, MO, USA	B6131

Trizma® base	Sigma, St. Louis, MO, USA	T1503
Triton X-100	Sigma, St. Louis, MO, USA	X-100
N,N,N′,N′-Tetramethylethylenediamine (TEMED)	Sigma, St. Louis, MO, USA	T9281
Trypan blue stain (0.4%)	Gibco, Life Technologies, Grand Island, NY, USA	15250-061
Sodium azide	Sigma, St. Louis, MO, USA	S8032
Ammonium persulfate	Sigma, St. Louis, MO, USA	A3678
Glycerol	Sigma, St. Louis, MO, USA	G7893
Sodium chloride	Fagron, Krakow, Poland	1002476
Glycine	Sigma, St. Louis, MO, USA	G7126
Sodium hydrogen phosphate dihydrate	Kemika, Zagreb, Croatia	1410207
Sodium dihydrogen phosphate dihydrate	Kemika, Zagreb, Croatia	1406707
RNAse	Sigma, St. Louis, MO, USA	R6513
**Cell culture**		
RPMI Medium 1640	Life Technologies, Grand Island, NY, USA	42401-018
Fetal bovine serum (FBS)	Life Technologies, Grand Island, NY, USA	10270-106
L-Glutamine 200mM	Life Technologies, Grand Island, NY, USA	25030-024
Penicillin/streptomycin	Life Technologies, Grand Island, NY, USA	15070-063

Supplementary Table 3. Published patient samples treated with 1000 nM AraC for 24 h (for GSEA analysis)

Patients	Age	Sex	Blasts in BM (%)	Cytology (AML subtype)	Cytogenetics	Molecular markers
AML1566	70	M	17	FAB M5	-	TET2 p.P363fs, TET2 p.S1284_F1285del, APC p.A1879S, PTPRT p.Y87T, PTPRT p.Y810R
AML2741	52	M	-	FAB M1	Complex	RUNX1 p.D198G, NOTCH1 p.R879G, IDH1 p.R132C
